# An Efficient and Sustainable Synthesis of the Antimalarial Drug Tafenoquine

**DOI:** 10.1021/acssuschemeng.2c05628

**Published:** 2022-12-07

**Authors:** Rahul D. Kavthe, Joseph R. A. Kincaid, Bruce H. Lipshutz

**Affiliations:** Department of Chemistry & Biochemistry, University of California, Santa Barbara, California 93106, United States

**Keywords:** malaria, antimalarial drug, tafenoquine, S_N_Ar reaction, nitro reduction, reductive amination, sustainability

## Abstract

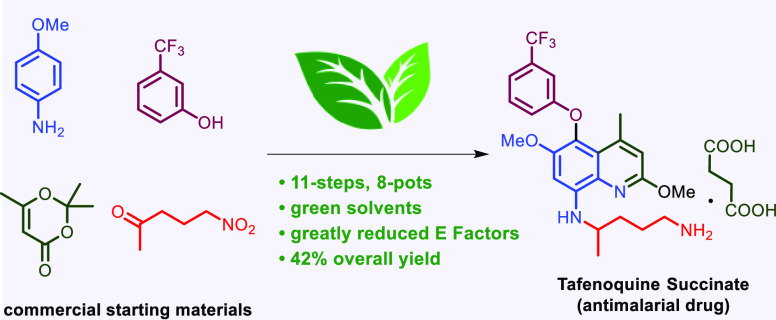

An 11-step, 8-pot synthesis of the antimalarial drug tafenoquine succinate was achieved in 42% overall yield using commercially available starting materials. Compared to the previous manufacturing processes that utilize environmentally egregious organic solvents and toxic reagents, the current route features a far greener (as measured by Sheldon’s E Factors) and likely more economically attractive sequence, potentially expanding the availability of this important drug worldwide.

## Abstract 2

The potent, Food and Drug Administration-approved antimalarial drug tafenoquine has been made using a streamlined, efficient, and environmentally responsible route.

## Introduction

**Figure 1:**
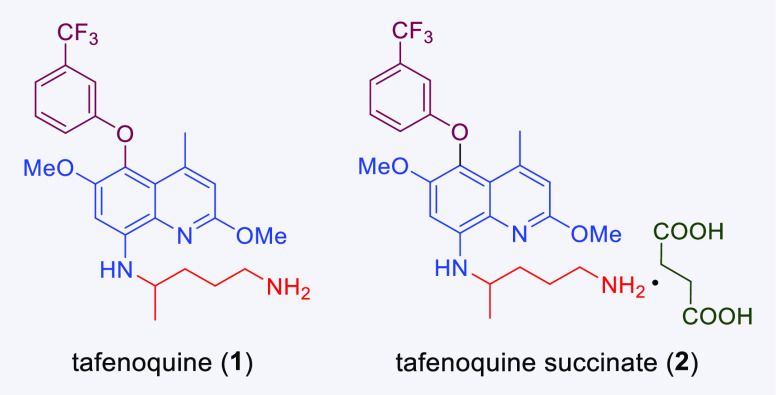
Structures of tafenoquine and its succinate salt.

Malaria is a life-threatening mosquito-borne parasitic disease responsible for an estimated 627,000 deaths globally in 2020.^1^ Many effective antimalarials have been developed (e.g., quinine, chloroquine, mefloquine, primaquine, and artemisinin-based combination therapies);^2−4^ however, their intensive use has led to the emergence of resistant Plasmodium strains as well as toxicological concerns. In response, a new antimalarial drug, tafenoquine (**1**), has been developed (Figure 1). Tafenoquine was recently approved by the US Food and Drug Administration as the first new single-dose treatment for *Plasmodium vivax* malaria in over 60 years.^5,6^ It is currently sold as the racemic succinate salt (**2**), under the names Krintafel (tablets of 150 mg) by GlaxoSmithKline (GSK)^7^ as well as Arakoda and Kodatef (tablets of 100 mg) by 60 Degrees Pharmaceuticals LLC.^8^ In comparison to previous generations of antimalarials, tafenoquine is considerably less toxic, has a longer plasma life (2–3 weeks),^9^ and is 10 times more potent.^10−12^ These combined features allow for single-dose treatment, compared to, e.g., the standard 14-day course of treatment associated with primaquine. Two synthetic routes to tafenoquine have previously been disclosed. The first involved 16 steps, ultimately affording the final drug in low overall yield (0.8%).^13^ An improved route reported by GSK involves 11 steps, leading to **2** in 14% overall yield.^14−16^ The key limitations of these routes include excess use of organic solvents along with toxic reagents (arsenic pentoxide)^13^ and low-yielding overall syntheses.^14,15^ Furthermore, recent increases in the stringency of environmental regulations are forcing pharmaceutical companies to pursue more sustainable processes.^17−22^ Therefore, there exists an urgent need for the development of both a green and economically attractive synthesis of tafenoquine.

**Scheme 1:**
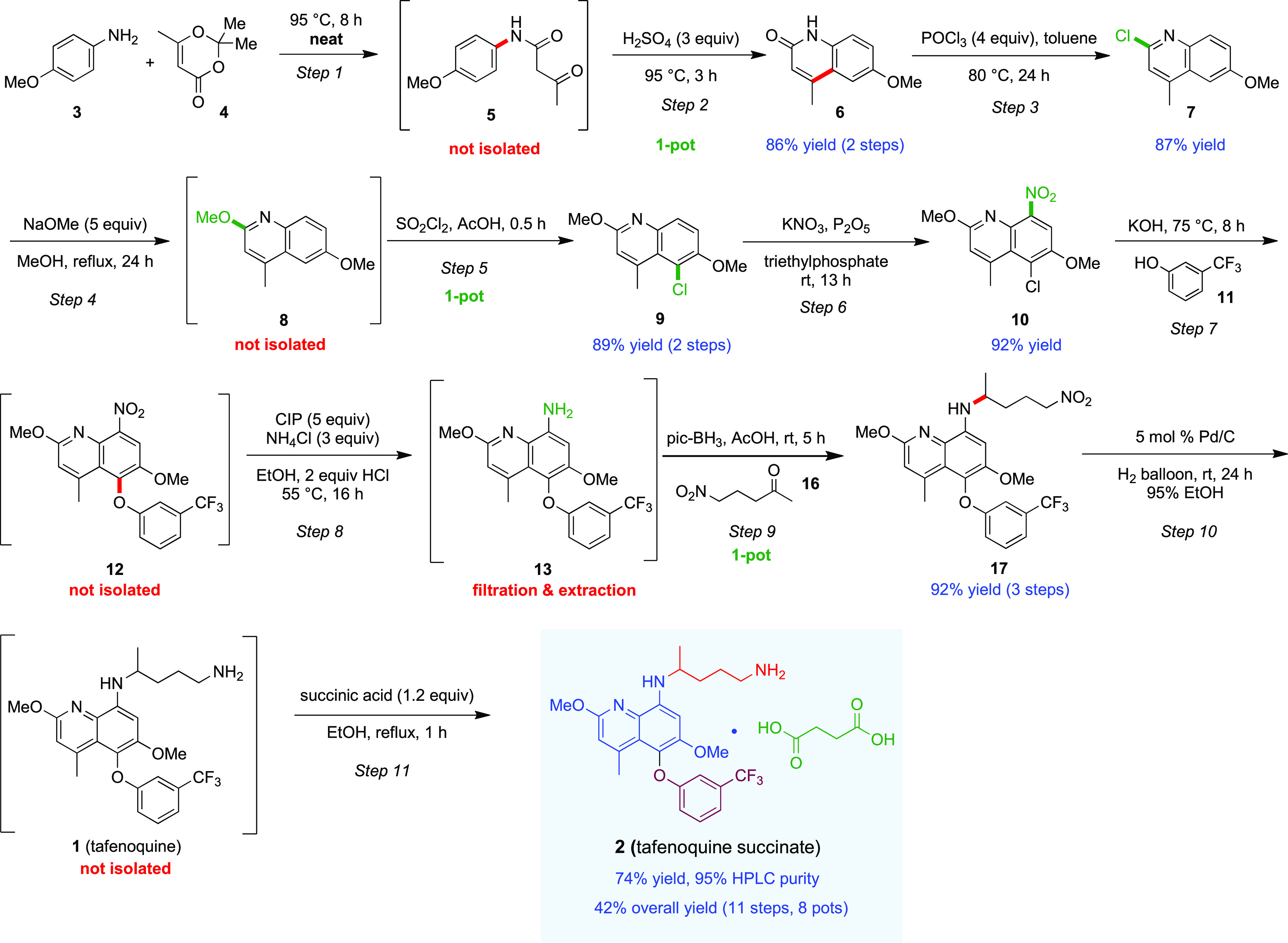
Overall Sequence to Tafenoquine Succinate (**2**)

In continuation of our group efforts to develop scalable routes to active pharmaceutical ingredients under cost-effective and environmentally friendly conditions,^23−26^ and in an ongoing collaboration with the Bill and Melinda Gates Foundation focused to date on pyronaridine (an antimalarial drug)^24^ and nirmatrelvir (the key ingredient in Pfizer’s Paxlovid for treatment of COVID-19),^23^ we now describe an environmentally responsible route to tafenoquine that simultaneously addresses these issues while maximizing both time and pot economies (Scheme 1).^27,28^ This has been accomplished by taking advantage of neat reactions following the Sheldon philosophy that “the best solvent is no solvent...”,^29−36^ and multistep, one-pot processes using environmentally preferred solvents.^37,38^

## Results and Discussion

### Two-Step, One-Pot Sequence En Route to Intermediate **6**

Scheme 1 illustrates the synthesis of intermediate lactam **6**. The first step, an amidation between **3** and methyl acetoacetate **4a** (Table 1), was performed using an aqueous solution of 2 wt % TPGS-750-M/H_2_O^39^ at a global concentration of 0.5 m to form **5** in 75% yield, along with the undesired side product **5a** (15% yield, entry 7) and unreacted starting material. In an effort to suppress side product formation and increase yield, the addition of an acid, such as BiBr_3_, HCl, and H_2_SO_4_, was screened. Unfortunately, none improved the reaction profile (entries 1–6). Scaling the best reaction conditions to 20 mmol led to only 41% isolated yield (following column chromatography) due to formation of byproduct **5a**. This vinylogous carbamate likely forms as a result of a more rapid reaction of aniline **3** toward condensation with the keto group in methyl acetoacetate, rather than the desired reaction with the ester moiety.

**Table 1:**
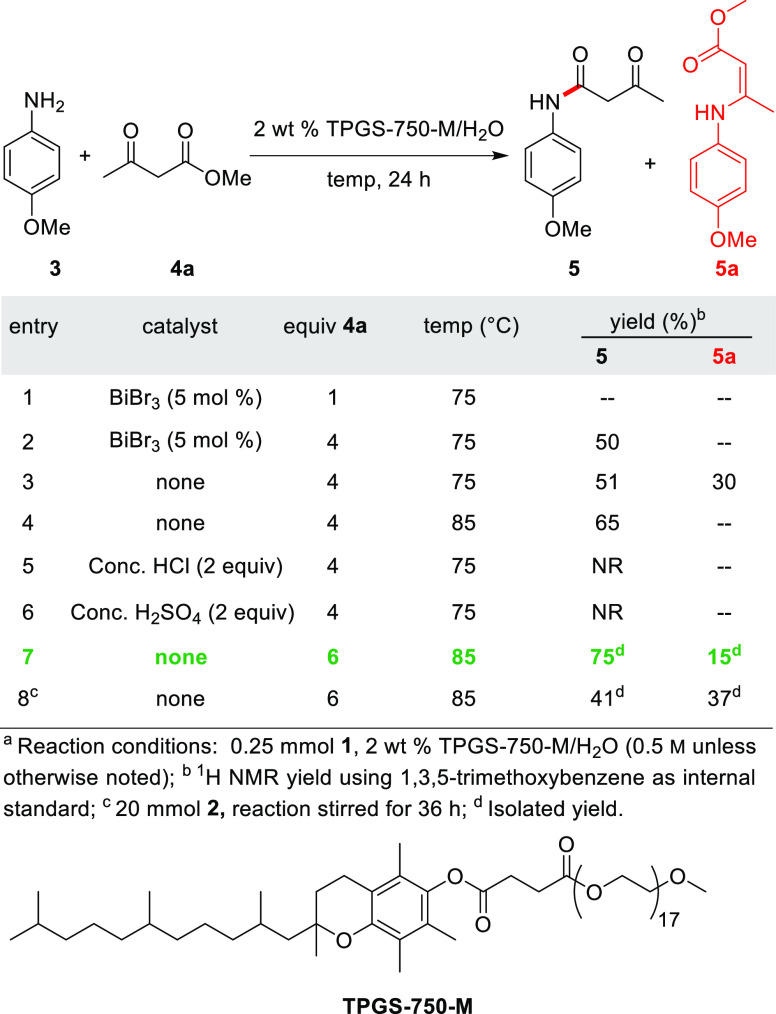
Optimization of Reaction Conditions to **5**^*a*^

To avoid this competing condensation, use of 2,2,6-trimethyl-4*H*-1,3-dioxin-4-one (**4**, TMD) was investigated, previously used by Clemens^40,41^ as an alternative coupling partner and shown to afford the same desired product. TMD is a stable equivalent of diketene which can be used to generate an acetylketene at higher temperatures (>82 °C)^42^ via a pseudo-retro-Diels-Alder reaction, eliminating acetone as the only byproduct. The resulting acetylketene intermediate can then be trapped by a nucleophile. The reaction between **3** and TMD (**4**) in 2 wt % TPGS-750-M/H_2_O at 85 °C over 12 h afforded the desired product in 87% isolated yield (Table 2, entry 1). Further increasing the reaction time to 24 h led to an improved yield of 95% (entry 2), whereas the reaction in the absence of this surfactant (i.e., “on water”) at reflux afforded product aniline **5** in a slightly lower yield (91%, entry 3). The latter result suggests that at higher temperatures the surfactant is not required to form the desired product **5**. Running the reaction neat led to an 89% isolated yield (entry 5). Despite this slightly lower yield at this smaller scale, neat conditions were taken as optimal as they allowed for telescoping using **5** in the next step without its unwanted hydrolysis (vide infra). Given the apparent benefits of the micellar medium, further investigation into the role of other surfactants (e.g., PS-750-M)^43^ in this chemistry is of future interest in our group.

**Table 2:**
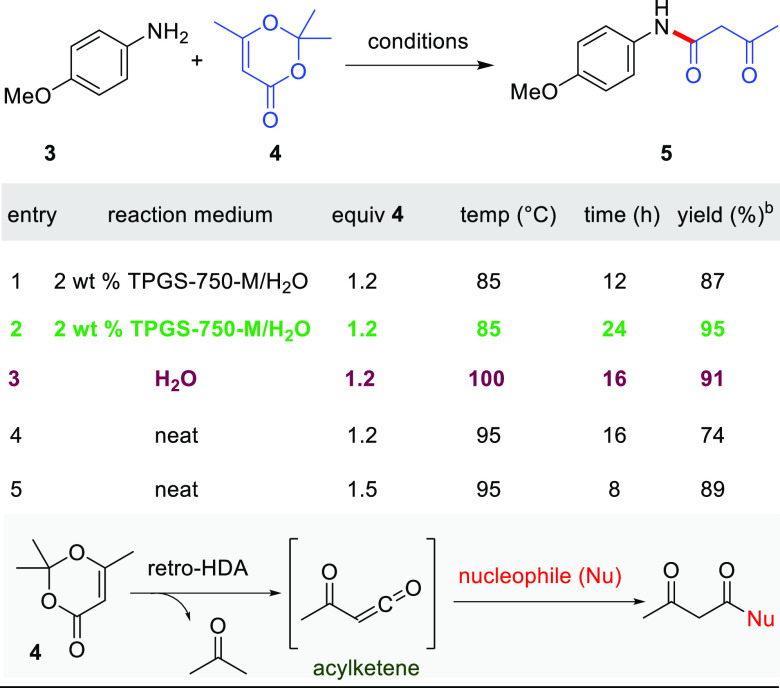
Optimization of Modified Reaction Conditions En Route to **5**^a^

a Reaction conditions: 1 mmol **1**, 0.5 M.

b Isolated yield.

Subsequent Knorr quinoline synthesis^44−47^ was employed in acidic media to convert the β-ketoanilide **5**, without isolation, to the desired 2-hydroxyquinoline **6**. Initially, the reaction was conducted in aqueous surfactant solution using three equiv. of conc. H_2_SO_4_, leading to quantitative formation of the undesired byproduct *p*-anisidine **3** (Table 3, entry 3). This outcome results from competitive hydrolysis of the amide bond in educt **5**, thereby indicating that an aqueous medium was incompatible with this transformation.

**Table 3:**
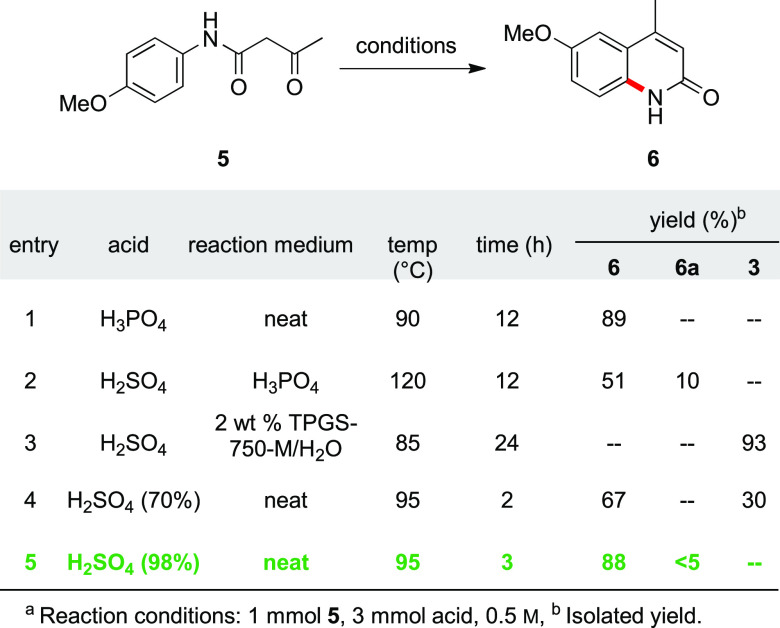
Optimization of Modified Reaction Conditions En Route to **6**^*a*^

**Figure 2:**
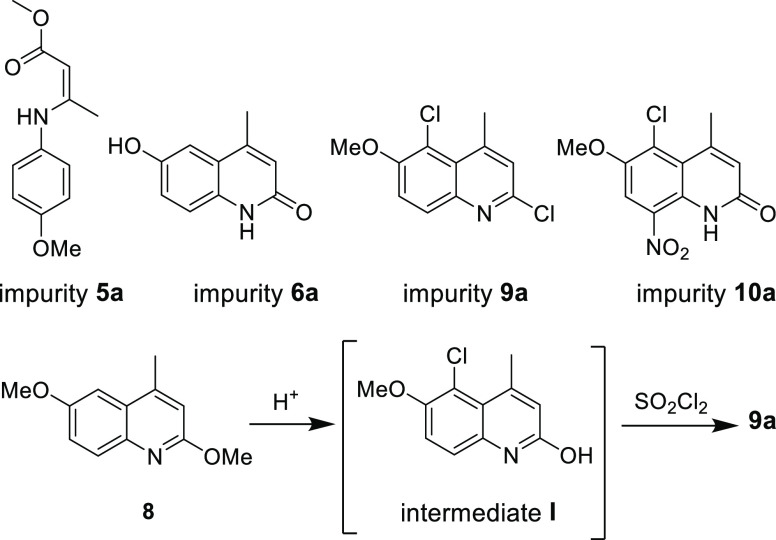
Impurities observed at various stages of the route to tafenoquine.

Under neat conditions, however, the use of three equiv. of conc. H_2_SO_4_ (0.3 M) at 95 °C afforded the desired product in 88% yield, along with traces (<5%) of demethylated side product **6a** (entry 5 and Figure 2). With the optimized stepwise synthesis of **6** in hand, a two-step, one-pot operation could then be devised to minimize handling (e.g., purification of initial product **6**). As expected, the sequence involving initial amidation to afford **5**, followed, in the same pot, by Knorr cyclization, smoothly afforded **6** in 86% isolated yield (see Scheme 1). It is interesting to note that the use of H_3_PO_4_ affords **6** in 89% isolated yield without any trace of byproduct **6a** or **3** (Table 3, entry 1). However, scaling this reaction to 5 mmol led to the formation of only trace amounts of product, with the rest being unreacted starting material. Ultimately, due to the significantly lower cost of H_2_SO_4_ on a large scale compared to H_3_PO_4_, the use of this acid was not investigated further.

### Conversion of Intermediate **6** to **7**

Deoxychlorination of intermediate **6** to form 2-chloroquinoline **7** was achieved using POCl_3_ (Scheme 1). Although water would not be tolerated in this step, toluene served nicely and could be recovered following isolation of product **7** (87% yield), thereby minimizing generation of organic waste. It was anticipated that generation of HCl during the reaction would lead to demethylation of the methoxy group, and thus triethylamine was initially included in the reaction mixture.^48^ However, omitting the base led to the same reaction outcome. Product **7** was purified by silica gel column chromatography to remove a brown impurity before proceeding to the next step. Alternatively, this material can be purified via recrystallization from EtOH.^49^

### Two-Step, One-Pot S_N_Ar/Chlorination Sequence En Route to Intermediate **9**

The S_N_Ar reaction between **7** and excess anhydrous sodium methoxide (5 equiv) in methanol under refluxing conditions led to product **8** in nearly quantitative yield (Scheme 1; specifically, see SI, section 3.5, Table S2, entry 5). Reducing the loading of sodium methoxide, e.g., from five to three equivalents led to a significant drop in conversion (to 53% yield; see SI, Section 3.5, Table S2). Chlorination at C-5 of quinoline **8** was effected with sulfuryl chloride in acetic acid at 60 °C for 30 min to afford the desired product **9** in 94% yield (see SI, Section 3.6, Table S3, entry 5). It should be noted that longer reaction times led to the formation of impurity **9a** (Figure 2). This impurity was produced via intermediate **I** (as shown in Figure 2), formed as a result of demethylation of the methoxy group in **8** under acidic conditions, followed by chlorination of intermediate **I**.

Modification of the workup associated with the initial S_N_Ar reaction allowed for direct conversion of **7** to **9** in a two-step, one-pot fashion. Thus, following the optimized S_N_Ar protocol (vide supra), excess NaOMe was quenched using four equivalents of AcOH, after which the reaction was concentrated to dryness to remove all traces of MeOH that might interfere with the subsequent chlorination step. Acetic acid (as a solvent) was then added, and chlorination was carried out as described above to afford **9** in 89% yield over both steps (in one-pot).

### Nitration of Intermediate **9** to Afford **10**

Various reagents were investigated for the nitration of **9** to arrive at nitroarene **10** (see SI, Section 3.7, Table S4). It was eventually found that in situ generation of N_2_O_5_ via dehydration of KNO_3_ (2 equiv) with P_2_O_5_ (4 equiv) in triethylphosphate (as a solvent) was optimal,^14^ leading to **10** in 92% isolated yield. The product was isolated simply by neutralizing the reaction mixture with aqueous NaHCO_3_ and collecting the resulting precipitate via filtration. Largely due to issues of solubility, very low yields (<10%) were obtained when other solvents (e.g., MeOH, CH_3_CN, sulfolane, 2-MeTHF, and DMSO) were used instead of triethylphosphate. Attempts to employ conventional nitrating conditions involving, e.g., HNO_3_ in H_2_SO_4_ led to rapid demethylation of the methoxy groups leading to impurity **10a** (Figure 2), as did the use of nitronium tetrafluoroborate (NO_2_BF_4_).

### Three-Step, One-Pot Sequence En Route to Intermediate **17**

The S_N_Ar reaction between nitroarene intermediate **10** and 3-(trifluoromethyl)phenol **11** to afford intermediate biaryl ether **12** was screened in both an aqueous surfactant medium and organic solvents. No conversion to the desired product was observed under aqueous conditions, and of the organic solvents tested, DMSO led to the highest yield 76% (see SI Section 3.8, Table S5, entry 4). Unfortunately, incomplete conversion under these conditions led to isolation problems, as **12** could not be easily separated from the starting material **10** via either column chromatography or recrystallization owing to close *R*_f_ values and poor solubility in several solvents. To obtain full consumption of starting material and facilitate product purification, use of neat conditions proved to be ideal (using 2 equiv **11** and equimolar base at 75 °C for 8 h), leading to **12** in 92% isolated yield (see SI, Section 3.8, Table S5, entry 9). The resulting product was of sufficiently high quality such that no further purification was needed at this stage. Nitro group reduction of intermediate **12** (without its isolation) to give aniline **13** (Scheme 1) was performed using two different protocols: (1) conventional Pd/C under H_2_ pressure, or (2) using carbonyl iron powder (CIP) that, as shown previously,^50^ smoothly reduces nitro groups in water. Under aqueous surfactant conditions, both Pd/C and CIP gave moderate yields of the desired product. These yields could be improved to 94% and 97%, respectively, by switching the medium to 95% EtOH (see SI, section 3.9, Table S6, entries 2, 4). The addition of one equivalent conc. HCl was necessary to achieve these results. Interestingly, a mixture of both types of media, such as 2 wt % TPGS-750-M/H_2_O and EtOH, led to only trace amounts of product formation.

An initial attempt at reductive amination between aniline **13** and ketone **14** (Table 4) was made by using the previously established protocol applied to the synthesis of Takeda’s drug TAK-954.^22^ These conditions called for an aqueous solution containing 2 wt % TPGS-750-M/H_2_O, together with MeOH (10 v/v %) as a cosolvent, and α-picoline borane as a hydride source (1.5 equiv). Under these conditions, the desired product was not formed; rather, only the starting material was fully recovered (Table 4, entry 2). Switching the cosolvent from MeOH to acetic acid led to a 54% yield (Table 4, entry 3). This suggested that AcOH was essential, presumably shifting the equilibrium toward desired product formation. Replacing the aqueous surfactant mixture by AcOH led to full conversion at rt to targeted product **15** in 91% isolated yield (after purification by column chromatography; entry 4). Although this reductive amination sequence is quite efficient, the approach suffers from inherent disadvantages, specifically with regard to the deprotection of the phthalimide group requiring hydrazinolysis, as well as separation of the phthalhydrazide byproduct that must be treated as waste. Hence, an alternative coupling partner **16** (i.e., 5-nitro-2-pentanone; Scheme 1) was selected for this final sequence, readily prepared via Michael addition of nitromethane to methyl vinyl ketone in the presence of catalytic amounts of sodium hydroxide.^51^ The reductive amination conditions initially optimized for the synthesis of intermediate **15** could be applied to the reaction between aniline **13** and ketone **16** to afford **17** in 90% yield (Table 4, entry 8) without further optimization. Once optimized conditions associated with each step had been determined, a three-step, one-pot synthesis was developed starting with **10** and ultimately affording **17** in 92% overall isolated yield, following recrystallization from ethanol. No column chromatography was needed at any stage for purification.

**Table 4:**
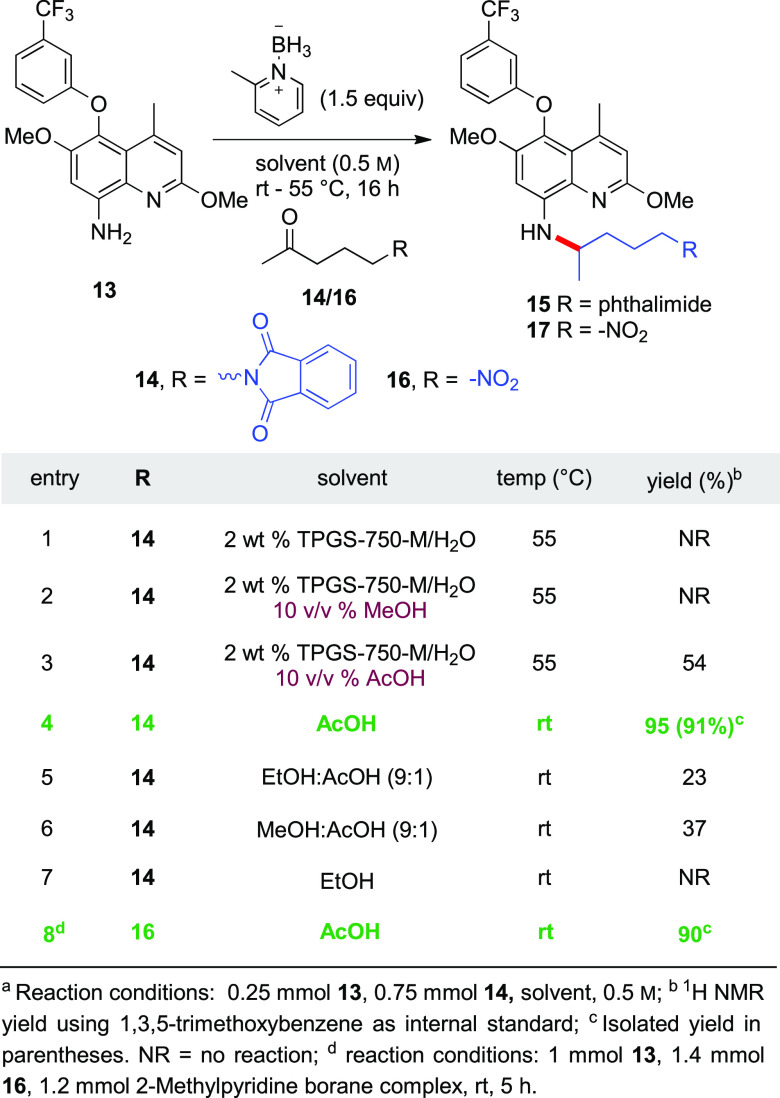
Optimization of Reductive Amination Conditions to Arrive at **15**^*a*^

### Two-Step, Tandem Sequence En Route to Tafenoquine Succinate (**2**)

Tafenoquine (**1**) was obtained by reduction in 95% EtOH of the nitro group-containing biaryl ether intermediate **17** using 5 mol % Pd/C under H_2_ pressure at rt for 24 h. Upon completion, the reaction mixture was filtered through a short plug of Celite to remove Pd/C. Tafenoquine succinate (**2**) was then formed by addition of succinic acid in EtOH. The precipitated amine succinate salt **2** was collected via filtration and obtained in 74% yield and 95% purity (by HPLC) over two steps (42% overall yield, 11 steps in 8 pots). Additional purification can be accomplished via recrystallization from EtOH, as described by GSK.^15^

### Alternative Gabriel Amine Synthesis Approach to Tafenoquine Succinate (**2**)

**Scheme 2:**
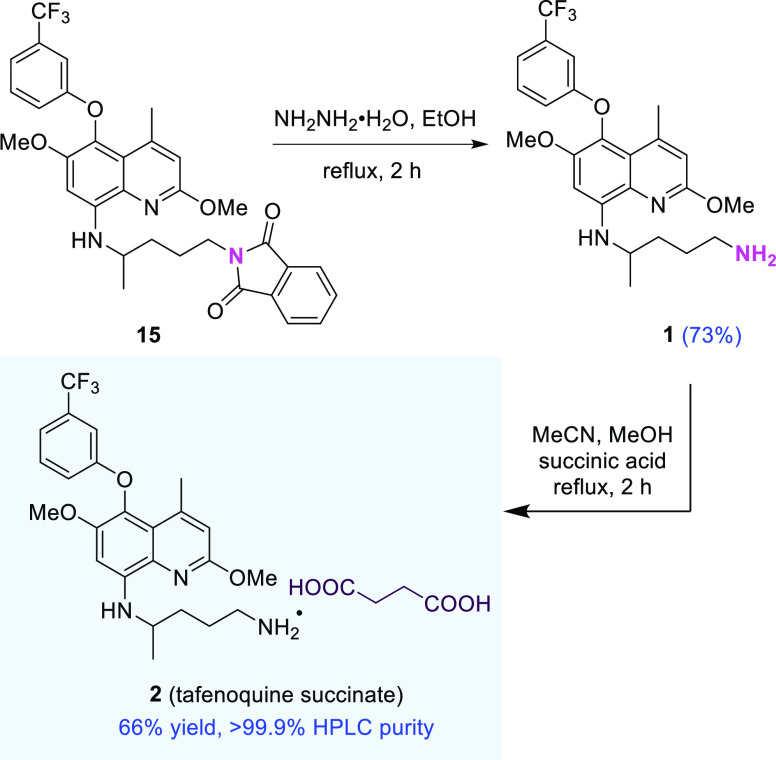
Alternative Route to Tafenoquine Succinate **2** from Phthalimide **15**

Although hydrazinolysis of phthalimide intermediate **15** in hot EtOH was found to be somewhat lower yielding and less attractive (vide supra and Scheme 2), it could be used to arrive at tafenoquine (**1**) in 73% yield following aqueous workup. This crude material was then converted to its succinic acid salt (**2**) in MeCN/MeOH. Collection of the precipitated product via filtration afforded **2** in 66% yield and >99% purity by HPLC (26% overall yield, 11 steps in 8 pots; ca. 42% using the nitro reduction route, vide supra).

### E Factor Determination

To compare the environmental footprint associated with our optimized route to **17** versus that by GSK,^14,15^ a complete E factor (cEF) was determined following the Roschangar procedure,^52^ as one measure of “greenness”, calculated as the ratio of the mass of waste generated to the mass of product. This was evaluated for the three-step tandem sequence leading to compound **17**, starting with 5-chloro-2,6-dimethoxy-4-methyl-8-nitroquinoline **10** (Scheme 1).

The results are indicative of a 3-fold decrease in waste creation, leading to a very low value of *E* = 17 (including aqueous waste streams (see SI, section 6) exemplifying the environmental friendliness of the described sequence. By contrast, the calculated cEF of *E* = 69 for the GSK sequence was characteristic of many environmentally egregious pharmaceutical processes that tend to have associated *E* factors between 25 and 100.^53^ Direct comparisons between the major reaction parameters between the GSK route^14,15^ and the current, far greener synthesis of tafenoquine are summarized in Table 5.

**Table 5:** Comparisons between GSK and This Route to Tafenoquine Succinate (**2**)

reaction parameter	GSK^14,15^	this work
amide bond formation (step 1)	solvent: xylene	solvent: none
	reaction temperature: reflux	reaction temperature: 95 °C
	reagent: ethylacetoacetate, triethanolamine	reagent: TMD
deoxychlorination (step 3)	POCl_3_ as a solvent	recoverable toluene minimal amount of POCl_3_
S_N_Ar reaction (step 7)	DMSO, reaction temperature: 100 °C product contains black tar impurity, requires activated carbon treatment and precipitation by toluene/hexane	neat, reaction temperature: 75 °C, no post purification
*E* factor for **10** to **17**	69	**17**
nitro reduction (step 8)	Pd/C	**carbonyl iron powder (CIP)**
overall yield	14%	**42%**

## Conclusions

In summary, an alternative synthetic route to the antimalarial drug tafenoquine relative to those currently known has been provided resulting in a more efficient and far greener process. This combination may significantly reduce both the cost and environmental footprint associated with this especially effective drug, potentially increasing its availability to those in need throughout the world.

## Supporting Information Available

Experimental procedures; analytical data for all new compounds; and NMR spectra of the products (PDF)
