# Supplementary material for: An Efficient and Sustainable Synthesis of the Antimalarial Drug Tafenoquine
Source: ACS Sustain Chem Eng. 2022 Dec 7;10(50):16896–902. doi: 10.1021/acssuschemeng.2c05628 (PMC9768812; doi:10.1021/acssuschemeng.2c05628)
Supplement: Supplementary file 1 — sc2c05628_si_001.pdf [file sc2c05628_si_001.pdf]

Supporting Information

An Efficient & Sustainable Synthesis of the Antimalarial Drug Tafenoquine

Rahul D. Kavthe, Joseph R. A. Kincaid, and Bruce H. Lipshutz*

Department of Chemistry and Biochemistry, University of California, Santa Barbara, CA 93106 USA

Phone : 805-893-2521
Fax : 805-893-8265
Email: lipshutz@chem.ucsb.edu
Website: https://lipshutz.chem.ucsb.edu/

Number of Pages: 59
Number of Figures: 10
Number of Schemes: 10
Number of Tables: 6

Contents

1. General Information S2
2. Synthetic Schemes S4
3. General Procedure S5
3.1 Synthesis of *p*-acetoanisidine (**5**) Step 1a: S5
3.2 Synthesis of *p*-acetoanisidine (**5**) Step 1: S6
3.3 Synthesis of 6-methoxy-4-methyl-2-quinolone (**6**): S6
3.4 Synthesis of 6-methoxy-4-methyl-2-chloroquinoline (**7**): S7
3.5 Synthesis of 2,6-dimethoxy-4-methylquinoline (**8**): S8
3.6 Synthesis of 2,6-dimethoxy-5-chloro-4-methylquinoline (**9**): S9
3.7 8-Nitro-2,6-dimethoxy-5-chloro-4-methylquinoline (**10**): S10
3.8 Synthesis of 8-nitro-2,6-dimethoxy-5-(3-trifluoromethyl)phenoxy-4-methylquinoline (**12**): S11

3.9 Synthesis of 8-amino-2,6-dimethoxy-5-(3-trifluromethyl)phenoxy-4-methylquinoline (**13**): S12

3.10 Synthesis of 8-amino-2,6-dimethoxy-5-(3-trifluromethyl)phenoxy-4-methylquinoline (**15**): S13

3.11 Synthesis of 8-Amino-2,6-dimethoxy-5-(3-trifluromethyl)phenoxy-4-methylquinoline (**1**) S14

3.12 Synthesis of tafenoquine succinate salt (**2**): S14

4. Procedures for the synthesis of tafenoquine succinate salt via Route 2: S15

4.1 Synthesis of 2,6-dimethoxy-4-methyl-*N*-(5-nitropentan-2-yl)-5-(3-(trifluoromethyl)phenoxy)quinolin-8-amine (**17**) S15

4.2 Synthesis of tafenoquine succinate salt (**2**) via nitro group reduction S16

5. Experimental procedure for tandem and 1-pot syntheses of intermediates S16

5.1 2-Step, 1-pot synthesis of intermediate **6** S16

5.2 2-Step, 1-pot synthesis of intermediate **9** S17

5.3 3-Step tandem sequence to afford intermediate **15** (Route 1) S18

5.4 3-Step tandem sequence to afford intermediate **17** (Route 2) S19

6. E Factor calculations S20

7. Purity analysis by HPLC S22

7.1 HPLC analysis of tafenoquine succinate salt **2** via hydrazinolysis route S22

7.2 HPLC analysis of tafenoquine succinate salt **2** via nitro group reduction route: S23

7.3 HPLC analysis for Intermediates 5 to 17 ....S23

8. Experimental Data S29

9. References S35

10. ^{1}H, ^{13}C NMR Spectra of synthesized products..S36

1. General Information

Preparation of the aqueous surfactant solution:

A 2 wt % TPGS-750-M/H_2O solution was prepared by dissolving TPGS-750-M in degassed HPLC grade water (20 mg/1 mL). TPGS-750-M[1] was made as described previously and is also commercially available. Reagents were purchased from Sigma-Aldrich, Combi-Blocks, and Alfa Aesar and used as received.

Chromatography:

Silica gel TLC plates (UV 254 indicator, thickness 200 mm standard grade, glass backed and 230-400 mesh from Merck) were used. The developed TLC plate was analyzed by a UV lamp (254

nm). The plates were further analyzed with the use of an aqueous ceric ammonium molybdate stain or ethanolic vanillin and developed with a heat gun. All commercially available reagents were used without further purification. Flash chromatography was performed using Silicycle Silicaflash® P60 unbonded grade silica.

NMR:

^{1}H and ^{13}C NMR were recorded at 25 ºC on either an Agilent Technologies 400 MHz, a Bruker Avance III HD 400 MHz, or a Varian Unity Inova 600 MHz spectrometer in $CDCl_3$ or DMSO-d_6 with residual $CHCl_3$ (^{1}H = 7.26 ppm, ^{13}C = 77.16 ppm) or DMSO (^{1}H = 2.54 ppm, ^{13}C = 40.45 ppm) as internal standard. Chemical shifts are reported in parts per million (ppm). The data presented will be reported as follows; chemical shift, multiplicity (s = singlet, bs = broad singlet, d = doublet, dd = doublet of doublet, t = triplet, q = quartet, quin = quintet, m = multiplet), coupling constant (if applicable), and integration.

HPLC:

HPLC analysis was performed on an Agilent 1220 series HPLC with an Agilent Poroshell HPH C18 column (4.6 x 50 mm, 2.7 μm). HPLC-grade solvents were obtained from Fischer Scientific.

2. Synthetic Schemes

Scheme S1: Stepwise Synthesis of Tafenoquine

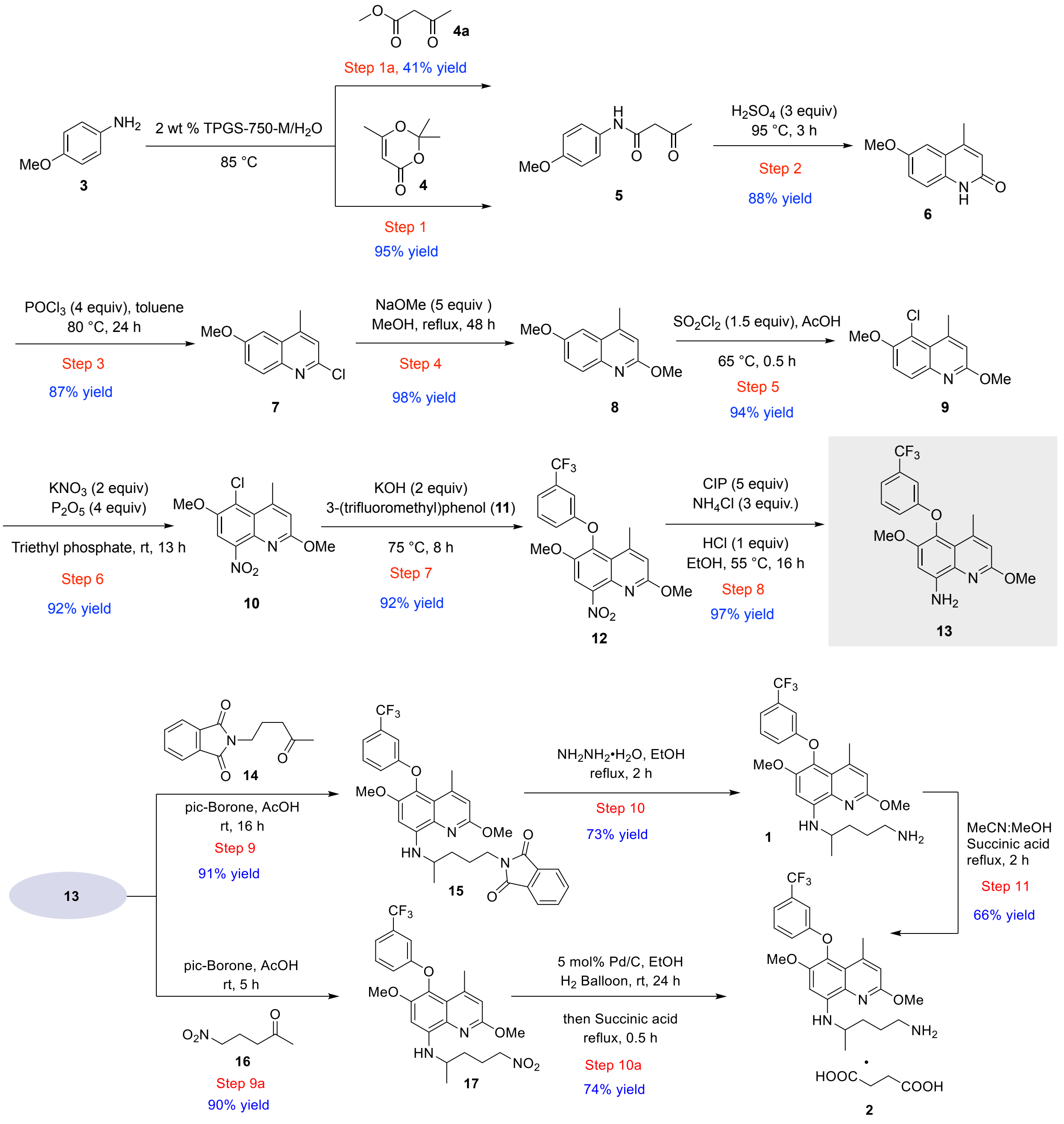

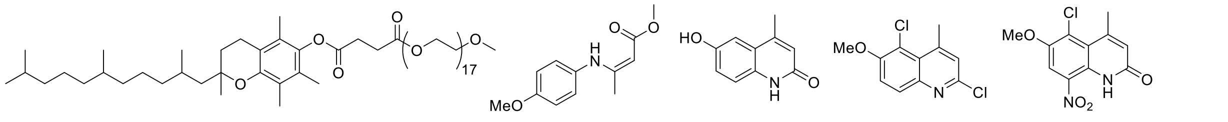

TPGS-750-M impurity **5a** impurity **6a** impurity **9a** impurity **10a**

Scheme S2: The Overall Route to Tafenoquine

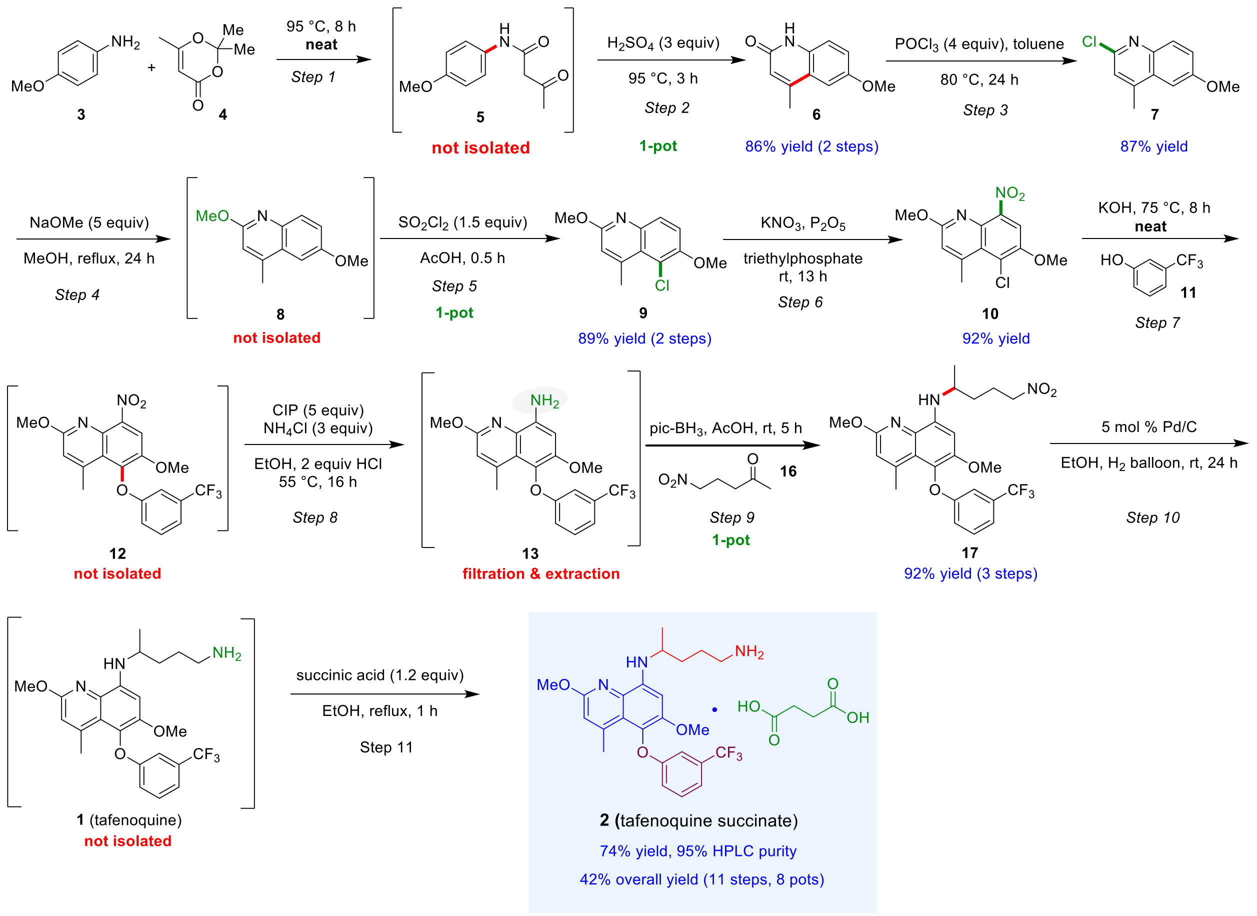

3. General Procedure

3.1 Synthesis of *p*-acetoanisidine (**5**) Step 1a:

Procedure: *p*-Anisidine **3** (2.46 g, 20 mmol), methyl acetoacetate **4a** (13 mL, 6 equiv) and 2 wt % TPGS-750-M/H_2O solution (40 mL) were added to a 100 mL RBF with a Teflon-coated magnetic stir bar. The reaction was stirred at 85 °C for 36 h (as monitored by TLC). After completion, the reaction was extracted with 2 x 25 mL of EtOAc. The organic layer was dried over anhydrous $MgSO_4$, filtered, and concentrated under vacuum. Purification was by flash chromatography using a hexane gradient (10-50% EtOAc/hexanes) to afford the desired compound as a light grey solid as the major product **5**, 41% (1.7 g), and as a yellow solid for the minor product **5a** (37%; 1.63 g).

3.2 Synthesis of *p*-acetoanisidine (**5**) Step 1:

Procedure: To a 2 dr vial equipped with a PTFE-coated magnetic stir bar was added *p*-anisidine **3** (1 mmol, 1 equiv, 123.2 mg), 2,2,6-trimethyl-4H-1,3-dioxin-4-one **4** (1.2 equiv, 1.2 mmol, 170.6 mg), and 2 wt % TPGS-750-M/H_2O (2 mL) after which the vial was sealed and allowed to stir at 85 °C for 24 h. After completion, the reaction was extracted with 2 x 5 mL of EtOAc. The organic layer was dried with anhydrous $MgSO_4$, filtered, and concentrated under vacuum. Purification by flash chromatography using hexanes and EtOAc (60:40) afforded the desired product **5** (196.8 mg, 95% yield, 99.1% purity by HPLC) as a brown solid.

Neat Conditions: To a 1 dr vial equipped with a PTFE-coated magnetic stir bar was added *p*-anisidine **3** (1 equiv, 1 mmol, 123.2 mg) and 2,2,6-trimethyl-4H-1,3-dioxin-4-one **4** (1.5 equiv, 1.5 mmol, 213 mg), then the vial was sealed and allowed to stir at 95 °C for 8 h. After completion, the reaction mixture was purified by flash chromatography using hexanes and EtOAc (60:40) to afford the desired product **5** (184 mg, 89% yield).

3.3 Synthesis of 6-methoxy-4-methyl-2-quinolone (**6**):

Procedure: To a 2 dr vial equipped with a PTFE-coated magnetic stir bar was added acylated compound **5** (1 equiv, 1 mmol, 207.2 mg) and 98% sulfuric acid (3 equiv, 3 mmol, 300.2 mg) at rt. The reaction was stirred at 95 °C for 3 h (as monitored by TLC). After cooling to rt, ice cold water was added to the reaction mixture and neutralized with $NaHCO_3$ and the resulting precipitate was filtered to afford the desired compound **6** (166 mg, 88% yield, >99% purity by HPLC) as a brown solid.

3.4 Synthesis of 6-methoxy-4-methyl-2-chloroquinoline (7):

Table S1: Synthesis of 6-methoxy-4-methyl-2-chloroquinoline

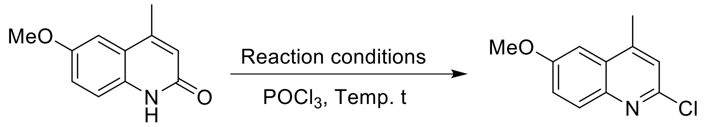

6 **7**

Entry	Solvent	Temp. (°C)	Time (h)	Yield (%)[a]
1	Neat[b]	80	2	83
**2**	**Toluene**	**80**	**24**	**87**
3	Toluene	80	16	86
4	2-Me-THF	75	24	79

Reaction conditions: 0.5 mmol **6**, 4 mmol $POCl_3$ and solvent 0.5 M, [a] Isolated yield, [b] $POCl_3$ (0.8 M) was used.

Procedure (entry 2): To a flame-dried 2 dr vial equipped with a PTFE-coated magnetic stir bar was added 6-methoxy-4-methyl-2-quinolone **6** (1 equiv, 0.5 mmol, 94.6 mg) and the vial was flushed with argon, then charged with anhydrous toluene (1 mL) under a flow of argon, followed by $POCl_3$ (4 mmol, 4 equiv, 187.0 µL) and the flask was sealed and allowed to stir at 80 °C for 24 h. Upon completion, the reaction mixture was transferred dropwise to a beaker containing 10 mL of ice water and 2 mL of 30% aqueous NH_4OH. The resulting precipitate was filtered, washed with water three times and dried under vacuum at rt to afford the desired compound **7** (90 mg, 87% yield).

Toluene recovery. The above reaction was performed on 10 mmol scale (1.89 g, 20 mL toluene added). After completion, the reaction mixture was quenched by addition of 30% aqueous NH_4OH. The resultant precipitate was filtered and toluene was separated from the aqueous layer using a separatory funnel. The crude organic layer was subsequently subjected to distillation to recover pure toluene (12.5 mL, 62.5%).

3.5 Synthesis of 2,6-dimethoxy-4-methylquinoline (**8**):

Table S2: Synthesis of 2,6-dimethoxy-4-methylquinoline

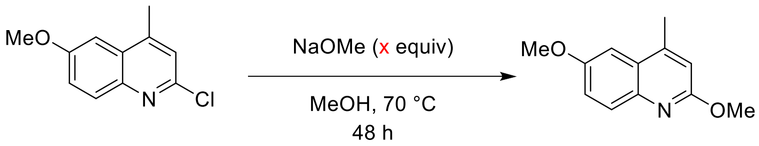

7 **8**

Entry	Equiv of NaOMe	Yield (%)
1	2	41
2	3	53
3	4	80
4	5	98(97)
**5**	**6**	**>99 (98)**
6	7	>99

[a] Reaction conditions: 0.5 mmol **7**, MeOH (0.5 M), [b] Yields were determined by crude ^{1}H NMR. [c] Isolated yields in parentheses.

Procedure (entry 5): In a 2-dram reaction vial containing a PTFE coated magnetic stir bar was added 2-chloro-6-methoxy-4-methylquinoline **7**, (0.5 mmol, 1 equiv, 103.8 mg). Anhydrous NaOMe (3 mmol, 6 equiv, 162.1 mg) was added in a glove box and the reaction vial was sealed with a rubber septum and then removed from the glove box, after which anhydrous MeOH (1 mL) was added via syringe. The reaction mixture was stirred at 70 °C for 48 h. After completion of the reaction (as monitored by TLC, 10:90 EtOAc:hexanes), the mixture was evaporated to remove solvent and purified by flash column chromatography using hexanes / EtOAc (95:5) to afford **8** (99.5 mg, 98% yield, 99.8% purity by HPLC) as a white solid.

3.6 Synthesis of 2,6-dimethoxy-5-chloro-4-methylquinoline (**9**):

Table S3: Synthesis of 2,6-dimethoxy-5-chloro-4-methylquinoline

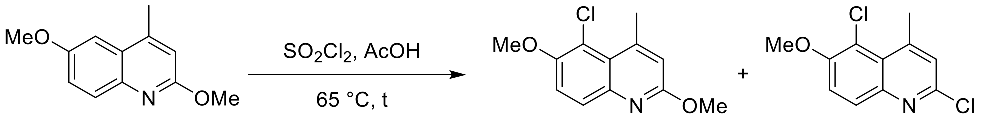

8 **9** **9a**

Entry	SO_2Cl_2 (equiv)	Time (h)	**9** Yield (%)[b]	**9a** Yield (%)[b]
1	1.2	2	54	--
2	1.5	2	60	--
3	2	2	87	7
4	2	1	90	5
**5**	**2**	**0.5**	**94**	**--**
6	2	12	80	19

[a] Reaction conditions: 0.5 mmol **8**, AcOH (1 M). [b] Isolated yield.

Procedure (entry 5): In a 1-dram reaction vial containing a PTFE coated magnetic stir bar was added compound **8** (0.5 mmol, 1 equiv, 101.6 mg) in glacial acetic acid (0.3 mL). It was then heated to 65 °C. To this solution, sulfuryl chloride (1 mmol, 2 equiv, 135.0 mg) in glacial acetic acid (0.2 mL) was added dropwise. The resulting solution was stirred for 30 min at 65 °C and then poured into 20 mL of ice-water. The resulting precipitate was filtered by suction filtration, washed with cold water and air dried to give the desired product **9** (112 mg, 94% yield, >99.5% purity by HPLC) as a white solid.

3.7 8-Nitro-2,6-dimethoxy-5-chloro-4-methylquinoline (**10**):

Table S4: Synthesis of 8-Nitro-2,6-dimethoxy-5-chloro-4-methylquinoline

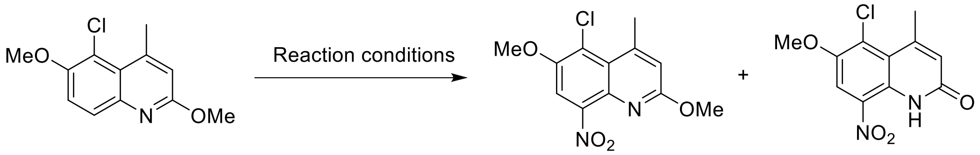

9 **10** **10a**

Entry	Nitrating reagent	Solvent	Temp. (°C)	Time	**8** Yield (%)[a]	**8a** Yield (%)[a]
1	HNO_3 (3 equiv)	H_2SO_4	0	0.5 h	11	33
2	KNO_3 (2 equiv)	H_2SO_4	0	0.5 h	15	40
3	HNO_3 (3 equiv)	H_2SO_4	0	5 min.	73	27
4	KNO_3 (2 equiv)	H_2SO_4	0	5 min.	55	44
5	KNO_3 (2 equiv)	AcOH	0	0.5 h	NR	--
6	KNO_3 (2 equiv)	2-Me-THF	60	6 h	NR	--
7	KNO_3 (2 equiv)	THF	60	3 h	NR	--
8	KNO_3 (2 equiv) P_2O_5 (4 equiv)	MeOH	60	3 h	NR	--
**9**	**KNO_3 (2 equiv) P_2O_5 (4 equiv)**	**triethyl phosphate**	**rt - 35**	**13 h**	**92[b]**	**--**
10	KNO_3 (2 equiv) P_2O_5 (4 equiv)	EtOAc	60	3 h	NR	--
11	KNO_3 (2 equiv) P_2O_5 (4 equiv)	MeCN	60	3 h	61	--
12	KNO_3 (2 equiv) P_2O_5 (4 equiv)	trimethyl phosphate	rt	3 h	57[b]	--
13	NO_2BF_4 (2 equiv)	MeCN	rt	5 h	Messy	--
14	KNO_3 (2.5 equiv) P_2O_5 (5 equiv)	DMSO	rt	16 h	NR	--

Reaction conditions: 0.25 mmol **9**, solvent 0.5 M, [a] Determined by crude ^{1}H NMR, [b] Isolated yield.

Procedure (entry 9): In a 2 mL reaction vial containing a PTFE coated magnetic stir bar and phosphorus pentoxide (1 mmol, 4 equiv, 283.9 mg) were added to a solution consisting of **9** (0.25 mmol, 1 equiv, 59.4 mg) and triethylphosphate (0.5 mL). The resulting pale-yellow suspension was stirred at rt for 12 h. The reaction mixture was then heated to 35 °C, followed by addition of KNO_3 (0.5 mmol, 2 equiv, 50.6 mg) in

one portion. The reaction mixture was stirred at 35 °C for an additional 1 h and poured into excess ice-water (ca. 10 mL). The resulting slurry was basified to pH 8 with $NaHCO_3$ and the precipitate was collected and air dried to yield the desired product **10** (65 mg, 92%, 99.4% purity by HPLC) as a yellow solid.

3.8 Synthesis of 8-nitro-2,6-dimethoxy-5-(3-trifluoromethyl)phenoxy-4-methylquinoline (**12**):

Table S5: Synthesis of 8-nitro-2,6-dimethoxy-5-(3-trifluoromethyl)phenoxy-4-methylquinoline

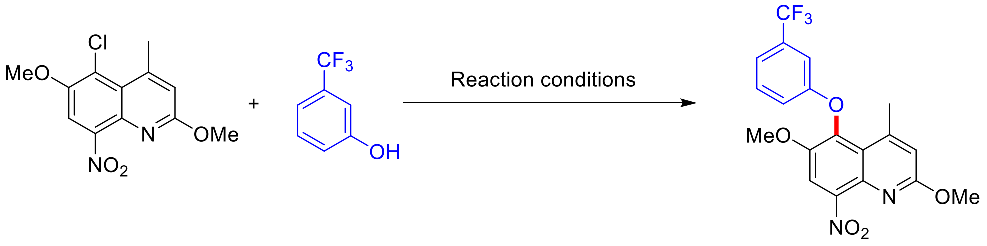

10 **11** **12**

Entry	Medium	Time (h)	Temp (°C)	Yield (%)[a]
1	2 wt % TPGS-750-M/H_2O	16	75	NR
2	2 wt % TPGS-750-M/H_2O DMSO 10 v/v %	16	75	NR
3	MeCN	16	70	18
4	DMSO	16	100	76
5	EtOH	16	70	trace
6	Sulfolane	16	80	46
7	--	12	75	82
8	--	8	75	84
**9[b]**	**--**	**8**	**75**	**92**

Reaction conditions: 0.25 mmol **10**, 0.6 mmol (1.5 equiv) of **11**, solvent (0.5 mL, 0.5 M unless otherwise noted),
[a] Isolated yield, [b] 2 mmol **10**, 4 mmol KOH and 4 mmol **11** were used.

Procedure (entry 4): In a 1-dram vial equipped with a PTFE-coated magnetic stir bar were added **11** (0.37 mmol, 1.5 equiv, 60.8 mg), DMSO (0.5 M), and potassium hydroxide (0.37 mmol, 21.0 mg). The mixture was heated to 100 °C and stirred until all the potassium hydroxide dissolved. Then, 5-chloro-2,6-dimethoxy-4-methyl-8-nitroquinoline **10** (1 equiv, 0.25 mmol, 70.7 mg) was added in one portion and the resulting solution was heated at 100 °C for 16 h. Upon completion of the reaction, the vial was cooled to rt. To the reaction mixture, water (0.5 mL) was added, and extracted with, EtOAc (3 x 3 mL), and the organic

layer was washed with 10% NaOH solution and then brine. The organic layer was dried over anhydrous $MgSO_4$. The mixture was concentrated *in vacuo* to afford crude product as a black solid, which was then purified by silica gel flash column chromatography using hexanes / EtOAc (90:10) to afford pure product **12** (78 mg, 76% yield, 99.5% purity by HPLC) as a yellow solid.

Neat conditions (entry 9): In a 2-dram vial containing a PTFE-coated magnetic stir bar was added 3-(trifluoromethyl)phenol **11** (4 mmol, 2 equiv, 648.4 mg) and KOH as a powder (4 mmol, 2 equiv, 224.4 mg). The reaction mixture was heated to 75 °C and held at this temperature until all the KOH dissolved (ca. 15 min). Then, 5-chloro-2,6-dimethoxy-4-methyl-8-nitroquinoline **10** (1 equiv, 2 mmol, 565.4 mg) was added in one portion and the resulting mixture was stirred for 8 h at 75 °C. Upon completion, DI water (0.5 mL) was added and the resulting yellow precipitate was collected via centrifugation, washed with 1 M NaOH (2 x 0.5 mL) and water (2 x 0.5 mL) by repeated resuspension in water and centrifugation, then dried under vacuum at rt to afford the pure product **12** as a yellow solid (750.1 mg, 92%).

3.9 Synthesis of 8-amino-2,6-dimethoxy-5-(3-trifluromethyl)phenoxy-4-methylquinoline (**13**):

Table S6: Synthesis of 8-amino-2,6-dimethoxy-5-(3-trifluromethyl)phenoxy-4-methylquinoline

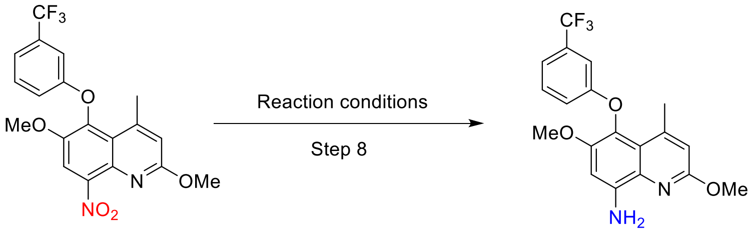

12 **13**

Entry	Reagent	Acid	Solvent	Yield (%)[a]
1	CIP (5 equiv)<br>NH_4Cl (3 equiv)	--	EtOH	45
**2**	**CIP (5 equiv)<br>NH_4Cl (3 equiv)**	**HCl (1 equiv)**	**EtOH**	**97**
3	CIP (5 equiv)<br>NH_4Cl (3 equiv)	HCl (1 equiv)	2 wt% TPGS-750-M/H_2O	57
4	2 mol% Pd/C<br>H_2 (1 atm)	HCl (1 equiv)	EtOH	94
5	2 mol% Pd/C<br>H_2 (1 atm)	HCl (1 equiv)	2 wt% TPGS-750-M/H_2O	63
6	CIP (5 equiv)<br>NH_4Cl (3 equiv)	HCl (1 equiv)	EtOH : 2 wt% TPGS-750-M/H_2O (1:1)	trace

Reaction conditions: 0.50 mmol **12**, solvent 0.5 M, [a] Isolated yield.

Procedure (entry 2): In a 1-dram vial were added 2,6-dimethoxy-4-methyl-8-nitro-5-(3-(trifluoro-methyl)phenoxy) quinoline **12** (1 equiv, 0.25 mmol, 102.1 mg), carbonyl iron powder (CIP; 1.25 mmol, 5.0 equiv, 69.8 mg), and NH_4Cl (0.75 mmol, 3.0 equiv, 40.1 mg). The vial was purged with argon, then EtOH (0.5 mL) and conc. HCl (1 equiv, 21 μL) were added. The vial was stirred at 55 °C until completion as monitored by TLC (10:90 EtOAc:hexanes). Saturated aqueous $NaHCO_3$ solution (50 uL) was added to the reaction and it was stirred briefly, then EtOAc (~0.5 mL) was added to the vial and it was then stirred briefly, after which the contents of the vial were filtered through ~1 cm of Celite in a pipette and rinsed with EtOAc (1 mL) (Note: filtration without prior addition of EtOAc has been found to be more difficult/slower). The solvent was removed by rotary evaporation and the product concentrated under vacuum to provide crude product. The crude product was purified by flash chromatography using EtOAc / hexanes (0 to 30%) to afford **13** as a purple solid (91.6 mg, 97% yield, 99.3% purity by HPLC).

Procedure (entry 4): In a 1-dram screw cap open top vial containing a Teflon-coated magnetic stir bar, 10 wt % Pd/C (5.3 mg, 0.005 mmol), 2,6-dimethoxy-4-methyl-8-nitro-5-(3-(trifluoromethylphenoxy) quinoline **12** (1 equiv, 0.25 mmol, 102.0 mg), EtOH (0.5 mL), and conc. HCl (1 equiv, 21 μL) were added. The reaction vial was closed and a septum was punctured with a needle (18 G) attached with a prefilled balloon of hydrogen gas. The headspace of the vial was replaced with H_2 by unscrewing the cap under positive H_2 flow for ca. 5 sec. Finally, the reaction mixture was stirred at rt for 18 h. After complete conversion of starting material, saturated $NaHCO_3$ solution (50 uL) was added to the reaction which was then stirred briefly. The reaction mixture was filtered through a short plug of silica and washed with EtOAc. Removal of the organic solvent led to crude product. Purification by flash chromatography using EtOAc / hexanes (0 to 30%) afforded **13** as a purple solid (88.5 mg, 94% yield).

3.10 Synthesis of 8-amino-2,6-dimethoxy-5-(3-trifluromethyl)phenoxy-4-methylquinoline (**15**):

Procedure (entry 4): In a 1-dram vial was added 8-amino-2,6-dimethoxy-5-(3-trifluromethyl)phenoxy-4-methylquinoline **13** (1 equiv, 0.25 mmol, 94.6 mg), *N*-(4-oxopentyl)phthalimide **14** (1.5 equiv, 0.375 mmol, 86.7 mg) and 2-picolineborane (1.5 equiv, 0.375 mmol, 39.0 mg) in glacial acetic acid (0.5 mL). The resulting mixture was stirred at rt for 16 h. After complete consumption of starting material, as indicated by TLC (30:70 EtOAc:hexanes), the reaction mixture was concentrated to dryness using a rotary evaporator to afford crude product. The crude product was purified by column chromatography using EtOAc:hexanes (1:4) to yield compound **15** (134.9 mg, 91% yield, 99.0% purity by HPLC) as a yellow solid.

3.11 Synthesis of 8-Amino-2,6-dimethoxy-5-(3-trifluromethyl)phenoxy-4-methylquinoline (**1**)

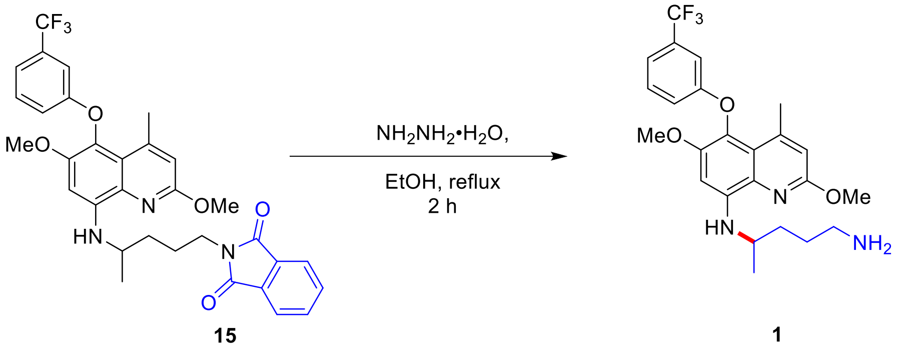

Scheme S3: Synthesis of 8-Amino-2,6-dimethoxy-5-(3-trifluromethyl)phenoxy-4-methylquinoline (**1**)

Procedure. In a 2-dram vial containing a PTFE coated magnetic stir bar was added 2-(4-((2,6-dimethoxy-4-methyl-5-(3-(trifluoromethyl)phenoxy)quinolin-8-yl)amino)pentyl)isoindoline-1,3-dione **15** (1 equiv, 0.421 mmol, 250 mg), 95% EtOH (2 mL), and hydrazine monohydrate (4.5 equiv, 1.9 mmol, 95 mg). The reaction was refluxed for 30 min resulting in a large amount of solid. Additional EtOH (0.5 mL) was added to dilute the reaction mixture, then the solution was refluxed for an additional 30 min. After complete consumption of starting material as indicated by TLC (20:80 EtOAc:hexanes), the reaction was cooled to rt. The solid was removed by filtration and washed with ethanol. The combined ethanol solution was concentrated to dryness using a rotary evaporator to give crude product. The crude product was dissolved in EtOAc and washed twice with 25% KOH solution and once with 5 mL of water. The organic layer was dried over anhydrous $MgSO_4$ and concentrated to dryness using a rotary evaporator to give **1** (142.6 mg, 73% yield), the desired product as a viscous yellow oil.

3.12 Synthesis of tafenoquine succinate salt (**2**):

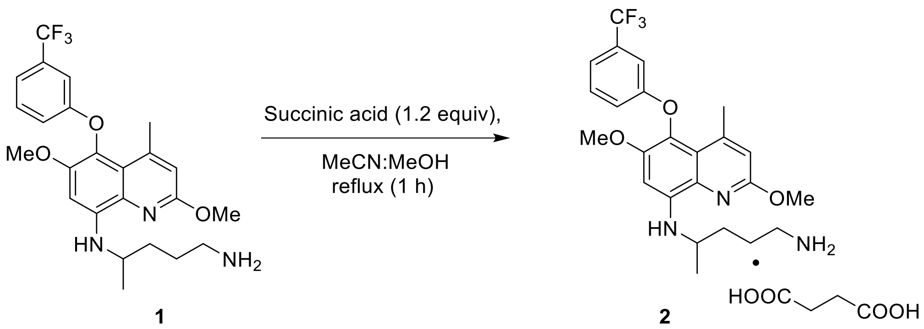

Scheme S4: Synthesis of tafenoquine succinate salt (**2**)

Procedure. In a 2-dram vial oily compound **1** (1 equiv, 0.30 mmol, 139.1 mg,) was dissolved in CH_3CN (2 mL). To the resulting solution was added succinic acid (0.36 mmol, 1.2 equiv, 42.5 mg) and CH_3OH (0.1 mL). The mixture was stirred at 50 °C for 1 h and allowed to cool to rt, then held at this temperature for about 2-3 h. The resulting precipitate was collected by suction filtration, washed with CH_3CN (1 mL) and air dried to afford tafenoquine succinate salt **2** (115.2 mg, 66% yield, >99.5% purity by HPLC) as an off-white solid.

4. Procedures for the synthesis of tafenoquine succinate salt via Route 2:

4.1 Synthesis of 2,6-dimethoxy-4-methyl-*N*-(5-nitropentan-2-yl)-5-(3-(trifluoromethyl)phenoxy)quinolin-8-amine (**17**)

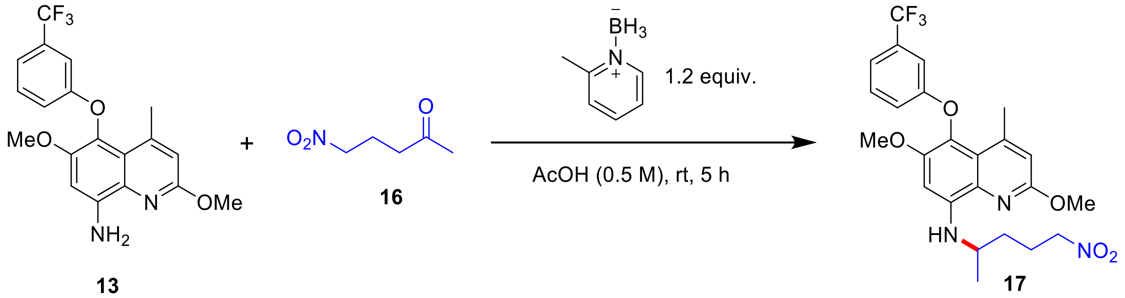

Scheme S5: Synthesis of 2,6-dimethoxy-4-methyl-*N*-(5-nitropentan-2-yl)-5-(3-(trifluoromethyl)phenoxy)quinolin-8-amine (**17**)

Procedure. In a 1-dram vial equipped with a PTFE-coated magnetic stir bar was added 8-amino-2,6-dimethoxy-5-(3-trifluromethyl) phenoxy-4-methylquinoline **13** (1 equiv, 1 mmol, 378.4 mg), 5-nitro-2-pentanone **16** (1.4 equiv, 1.4 mmol, 184 mg), and 2-picolineborane (1.2 equiv, 1.2 mmol, 124.7 mg) in glacial acetic acid (2 mL). The resulting mixture was stirred at rt for 5 h. After complete conversion of starting material as indicated by TLC (30:70 EtOAc:hexanes), the solvent was evaporated to give a brown oily residue, which was then dissolved in ethanol (0.5 mL). The solution was cooled to 5 °C for 24 h and the resulted crystals were filtered off, washed with cold ethanol (0.2 mL), and dried to afford compound **17** (446 mg, 90% yield, 97.9% purity by HPLC) as a yellow solid.

4.2 Synthesis of tafenoquine succinate salt (**2**) via nitro group reduction

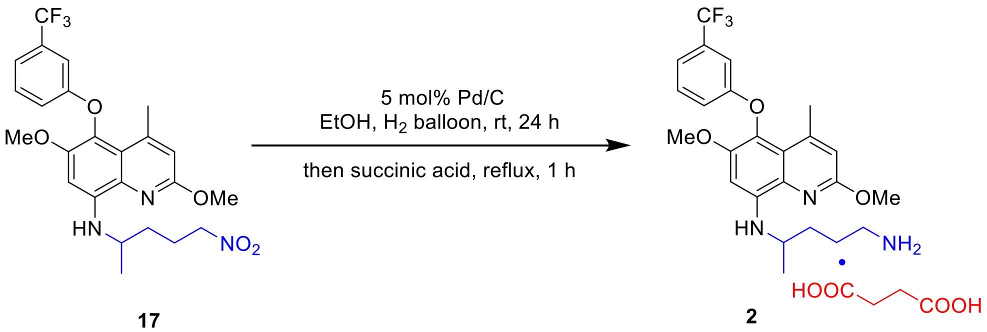

17 **2**

Scheme S6: Synthesis of tafenoquine succinate salt (**2**) via nitro group reduction

Procedure. In a 1-dram vial was added 8-amino-2,6-dimethoxy-5-(3-trifluromethyl)phenoxy-4-methylquinoline **17** (1 equiv, 0.25 mmol, 123.4 mg), ethanol (0.5 mL, 0.5 M), and 10 wt % Pd/C (0.0125 mmol, 5 mol %, 13.3 mg). After degassing the reaction mixture with hydrogen for 5 min, the reaction mixture was allowed to stir at rt for 24 h under one atmosphere of hydrogen. The catalyst was filtered off and ca. one third of the solvent was removed by evaporation *in vacuo*. Succinic acid (0.3 mmol, 1.2 equiv, 35.4 mg) was added, and the mixture was refluxed for 30 min. The solution was then cooled to 0-5 °C for 4 h. The resulting precipitate was collected by suction filtration, washed with CH_3CN (1 mL) to afford tafenoquine succinate salt **2** (107 mg, 74% yield, 95% purity by HPLC) as an off-white solid.

5. Experimental procedure for tandem and 1-pot syntheses of intermediates

5.1 2-Step, 1-pot synthesis of intermediate **6**

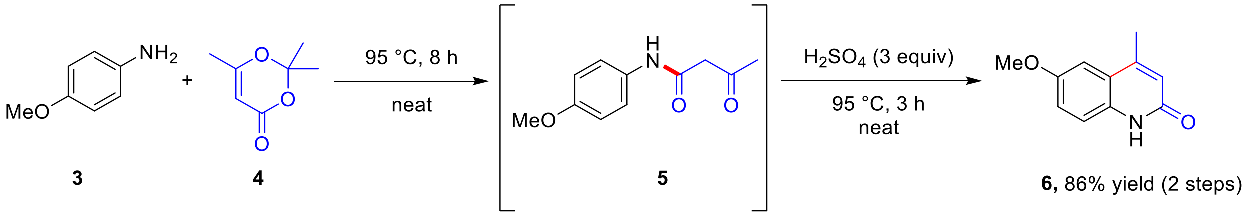

3 **4** **5** **6,** 86% yield (2 steps)

Scheme S7: 2-Step, 1-pot synthesis of intermediate **6**

To a 2-dram vial equipped with a PTFE-coated magnetic stir bar was added *p*-anisidine **3** (2.5 mmol, 1 equiv, 307.9 mg) and 2,2,6-trimethyl-4H-1,3-dioxin-4-one **4** (3.75 mmol, 1.5 equiv, 533 mg) after which the vial was sealed and allowed to stir at 95 °C for 8 h. Complete conversion of starting material was determined by TLC ((30:70 EtOAc:hexanes),). To the same reaction mixture was then added 98% sulfuric

acid (3 equiv, 7.5 mmol, 750 mg) at rt. The reaction was stirred at 95 °C for 3 h while the extent of conversion was monitored by TLC (50:50 EtOAc:hexanes). After cooling to rt, ca. 1 mL of ice cold water was added dropwise to the reaction mixture which was then neutralized by dropwise addition of saturated $NaHCO_3$ and the resulting precipitate was filtered and dried under high vacuum for 16 h to afford the desired compound **6** (406.6 mg, 86% yield) as a brown solid.

5.2 2-Step, 1-pot synthesis of intermediate **9**

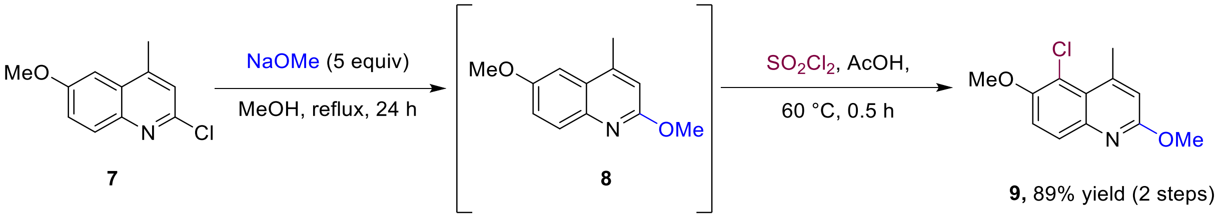

Scheme S8: 2-Step, 1-pot synthesis of intermediate **9**

In a 1-dram vial containing a PTFE-coated magnetic stir bar was added 2-chloro-6-methoxy-4-methylquinoline **7** (1 equiv, 0.5 mmol, 103.8 mg). Anhydrous NaOMe (5 equiv, 2.5 mmol, 135.1 mg) was then added to the vial in a glove box and the reaction vial was sealed with a rubber septum, removed from the glove box, to which was then added anhydrous MeOH (1 mL) via syringe. The reaction mixture was stirred at 70 °C for 24 h. After completion of the reaction as monitored by TLC (5:95 EtOAc:hexanes), the reaction mixture was cooled to rt, then AcOH (4 equiv, 2 mmol, 114 µL) was added to quench excess NaOMe and the mixture was stirred for 10 min, after which MeOH was removed *in vacuo*. To the crude residue was added AcOH (666 µL) followed by dropwise addition of a solution of SO_2Cl_2 (2 equiv, 1 mmol, 81 µL) in AcOH (222 µL) and the reaction was stirred at 60 °C for 0.5 h, then poured into ice water (ca. 20 mL). The resulting precipitate was collected by suction filtration, washed with cold water, and air dried to afford **9** as a white solid (105.3 mg, 89%).

5.3 3-Step tandem sequence to afford intermediate **15** (Route 1)

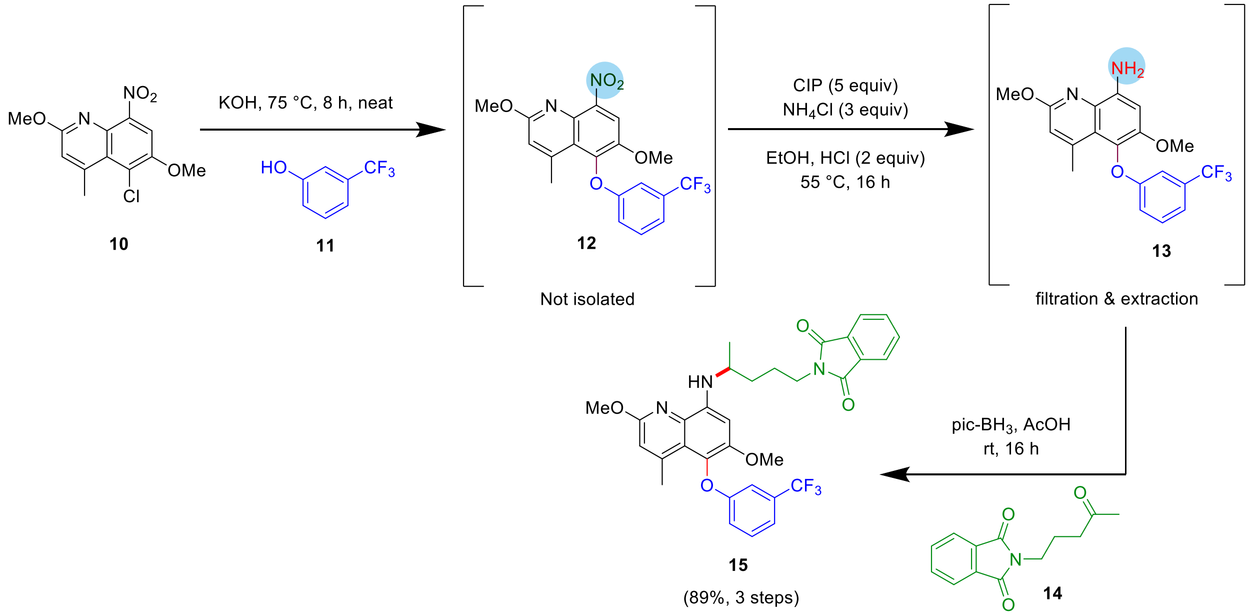

Scheme S9: 3-Step tandem sequence to afford intermediate **15**

In a 1-dram vial containing a PTFE-coated magnetic stir bar was added 3-(trifluoromethyl)phenol **11** (0.5 mmol, 2 equiv, 81.1 mg) and KOH powder (0.5 mmol, 2 equiv, 28.1 mg). The reaction mixture was heated to 75 °C and held at this temperature until all the KOH had dissolved (about 15 min). Then, 5-chloro-2,6-dimethoxy-4-methyl-8-nitroquinoline **10** (0.25 mmol, 1.0 equiv, 71.7 mg) was added in one portion and the resulting solution was heated at this temperature for 8 h. After complete conversion of starting material as indicated by TLC (10:90 EtOAc:hexanes), the reaction mixture was cooled to rt and carbonyl iron powder (CIP; 1.25 mmol, 5.0 equiv, 69.8 mg) and NH_4Cl (0.75 mmol, 3 equiv, 40.1 mg) were added. The vial was purged with argon, then 95% EtOH (0.5 mL) and conc. HCl (2 equiv, 42 ul) were added at rt, and the resulting mixture was allowed to stir at 55 °C until completion as monitored by TLC (10:90 EtOAc:hexanes). Saturated $NaHCO_3$ solution (50 uL) was added to the reaction mixture which was stirred briefly, then EtOAc (~0.5 mL) was added to the vial and after brief stirring, the contents of the vial were filtered through ~1 cm of Celite in a pipette and rinsed with EtOAc (0.5 mL). The solvent was removed by rotary evaporation and the product concentrated under vacuum to provide crude product as a thick purple liquid which was used without further purification.

In a 1-dram vial with a PTFE-coated magnetic stir bar, the crude product from the previous step was used along with *N*-(4-oxopentyl)phthalimide **14** (0.375 mmol, 1.5 equiv, 86.7 mg) and 2-picolineborane (0.375 mmol, 1.5 equiv, 40.0 mg). Glacial acetic acid (0.5 mL) was then added, and the reaction was stirred at rt

for 16 h, then evaporated to dryness *in vacuo*. The resulting product was purified by silica gel flash column chromatography using EtOAc: hexanes (3:1) to afford the desired compound **15** in 89% yield (132.5 mg) as a yellow solid.

5.4 3-Step tandem sequence to afford intermediate **17** (Route 2)

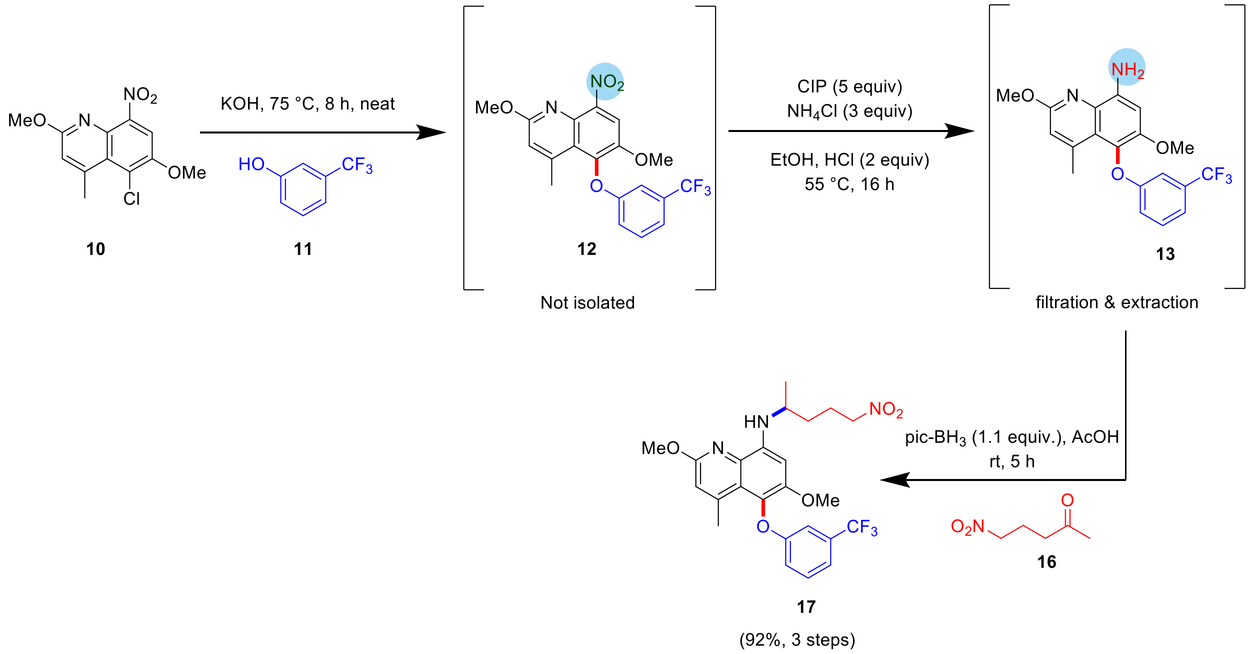

Scheme S10: 3-Step tandem sequence to afford intermediate **17**

In a 1-dram vial containing a PTFE-coated magnetic stir bar was added 3-(trifluoromethyl)phenol **11** (0.5 mmol, 2 equiv, 81.1 mg) and KOH powder (0.5 mmol, 2 equiv, 28.1 mg). The reaction mixture was heated to 75 °C and held at this temperature until all the KOH was dissolved (about 15 min). Then, 5-chloro-2,6-dimethoxy-4-methyl-8-nitroquinoline **10** (0.25 mmol, 1.0 equiv, 71.7 mg) was added in one portion and the resulting solution was kept at this temperature for 8 h. After complete conversion of starting material as indicated by TLC (10:90 EtOAc:hexanes), the reaction mixture was cooled to rt and carbonyl iron powder (CIP; 1.25 mmol, 5.0 equiv, 69.8 mg) and NH_4Cl (0.75 mmol, 3 equiv, 40.1 mg) was added. The vial was purged with argon, then 95% EtOH (0.5 mL) and HCl (2 equiv, 42 uL) were added at rt, and the resulting mixture was allowed to stir at 55 °C until completion as monitored by TLC (10:90 EtOAc:hexanes). The EtOH was evaporated under reduced pressure then saturated $NaHCO_3$ solution (200 µL) and EtOAc (~0.5 mL) were added to the vial which was further stirred briefly, after which the contents of the vial were filtered through ~1 cm of Celite in a pipette and rinsed with EtOAc (0.5 mL). The solvent was removed by rotary evaporation and the product concentrated under vacuum to provide crude product as a thick purple liquid which was used without further purification.

In a 1-dram vial with a PTFE-coated magnetic stir bar, the crude product from the previous step was used along with 5-nitro-2-pentanone **16** (0.37 mmol, 1.4 equiv, 49.2 mg) and 2-picolineborane (0.3 mmol, 1.2 equiv, 32.2 mg). Glacial acetic acid (0.5 mL) was then added, and the reaction was stirred at rt for 5 h and evaporated to dryness *in vacuo*. The obtained the brown oily residue dissolved in ethanol (0.2 mL). The solution was cooled to 5 °C for 24 h. The resulted crystals were filtered off, washed with cold ethanol (0.1 ml) and dried *in vacuo* at rt for 3-4 h to afford the desired compound **17** (113.8 mg, 92% yield) as a yellow solid.

6. Complete E Factor (cEF) calculations

To compare the present process with the conventional one, the complete E Factor (cEF) was used according to its definition by Roschangar et al.[10]

Masses of waste and products are those form the protocols described in the SI section 5.4. Masses were normalized to 1 mmol of starting material for the first pot, then subsequent pots were adjusted down in scale based on the yields of the preceding pots.

- **This work (3-step, 1-pot sequence):**

  excess phenol = 0.056 g
  excess KOH = 0.1622
  NH_4Cl = 0.160 g
  HCl = 0.25 g
  sat. $NaHCO_3$ = 0.2 gEtOAc = 3.608 g
  excess 5-nitro-2-pentanone = 0.0657 g
  AcOH = 2.02 g
  EtOH = 1.0824 g

Total waste (including aqueous) = 7.6063 g
Overall yield = 92%
Total product = 1 mmol x 0.4935 g/mmol x 0.92 = 0.4540 g
E Factor (including aqueous waste) = 7.6063/0.4540 = **17**

- **Literature:** (First two step from WO1997013753A1) and (Last step from WO03/093239A2)

  S_NAr reaction

  excess phenol = 3.24 g
  excess KOH = 1.14 g
  DMSO = 143 g
  toluene = 606.2 g
  hexanes = 330 g
  H_2O = 250 g
  carbon black = 5 g
  total waste = 1338.58 g

product = (47.3 g), 87.2 % yield

Reduction

excess hydrazine hydrate = 0.3924 g
EtOH = 2.8379 g
H_2O = 2.725 g

Reductive amination reaction

AcOH = 2.4265 g
excess 5-nitro-2-pentanone = 0.0525 g
EtOH = 0.9559 g

total waste (including aqueous) = 19.4548 g
overall yield = 57.3%
total product = 1 mmol x 0.4935 g/mmol x 0.573 = 0.2828 g
E Factor (including aqueous waste) = 19.4548/0.2828 = **69**

7. Purity analysis by HPLC

7.1 HPLC analysis of tafenoquine succinate salt **2** via hydrazinolysis route

A = 0.1 % formic acid in 5 mM aqueous ammonium acetate

B = 0.1 % formic acid in acetonitrile

HPLC Method

time (min)	flow rate (mL/min)	%A	%B
0	0.5	95	5
2	1	95	5
4.5	1	60	40
7	1	30	70
12	1	10	90
15	1	10	90
15.1	1	95	5
20	1	95	5

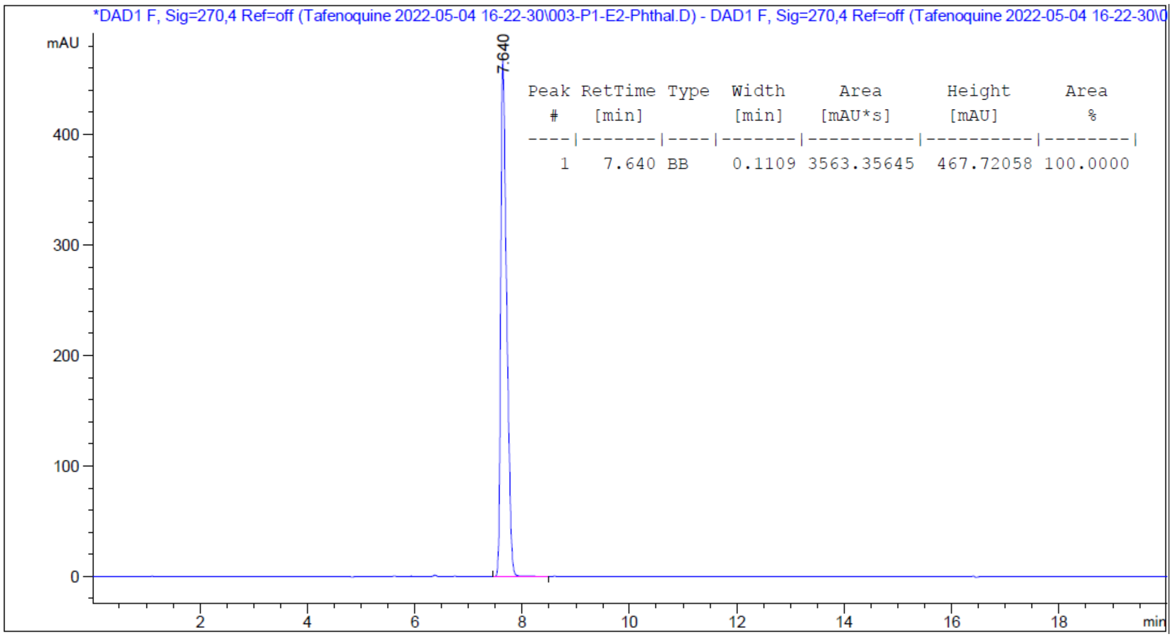

7.2 HPLC analysis of tafenoquine succinate salt **2** via nitro group reduction route:

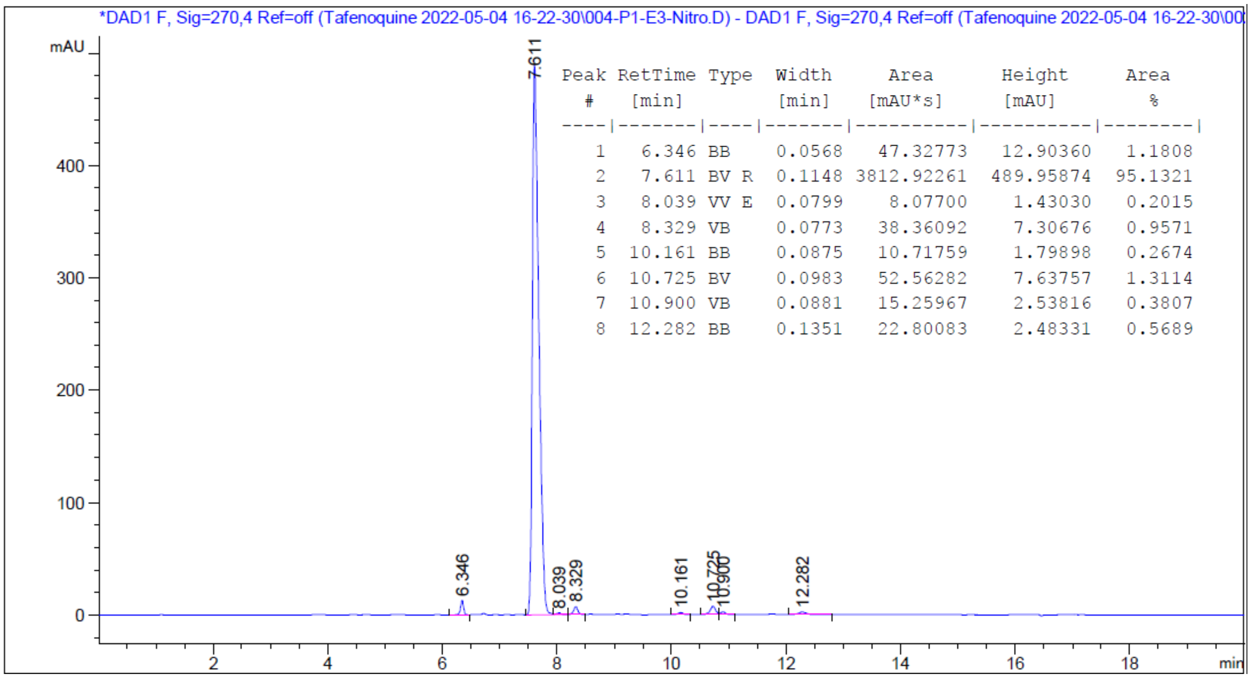

Peak #	RetTime [min]	Type	Width [min]	Area [mAU*s]	Height [mAU]	Area %
1	6.346	BB	0.0568	47.32773	12.90360	1.1808
2	7.611	BV R	0.1148	3812.92261	489.95874	95.1321
3	8.039	VV E	0.0799	8.07700	1.43030	0.2015
4	8.329	VB	0.0773	38.36092	7.30676	0.9571
5	10.161	BB	0.0875	10.71759	1.79898	0.2674
6	10.725	BV	0.0983	52.56282	7.63757	1.3114
7	10.900	VB	0.0881	15.25967	2.53816	0.3807
8	12.282	BB	0.1351	22.80083	2.48331	0.5689

7.3 HPLC analysis for Intermediates 5 to 17

Methods used for HPLC analysis:

Method 1: HPLC analysis was performed on an Agilent 1220 series HPLC with a Lux 5u Cellulose-2 column (250 x 4.6 mm, 5 µm) at a flow-rate of 1.25 mL/min using 5% v/v isopropanol/hexanes.

Method 2: HPLC analysis was performed on an Agilent 1220 series HPLC with a Lux 5u Cellulose-2 column (250 x 4.6 mm, 5 µm) at a flow-rate of 1.25 mL/min using 20% v/v isopropanol/hexanes.

Method 3: HPLC analysis was performed on an Agilent 1220 series HPLC with a Lux 5u Cellulose-2 column (250 x 4.6 mm, 5 µm) at a flow-rate of 1.25 mL/min using 50% v/v isopropanol/hexanes.

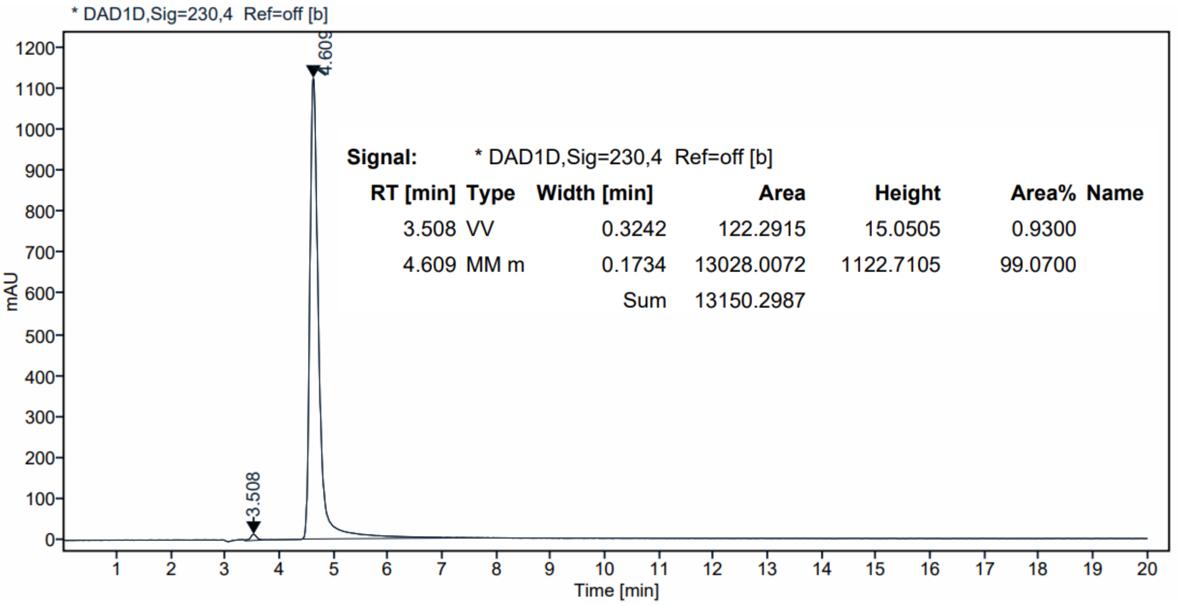

Figure S1: HPLC chromatogram used to determine purity of **5** (HPLC method 3)

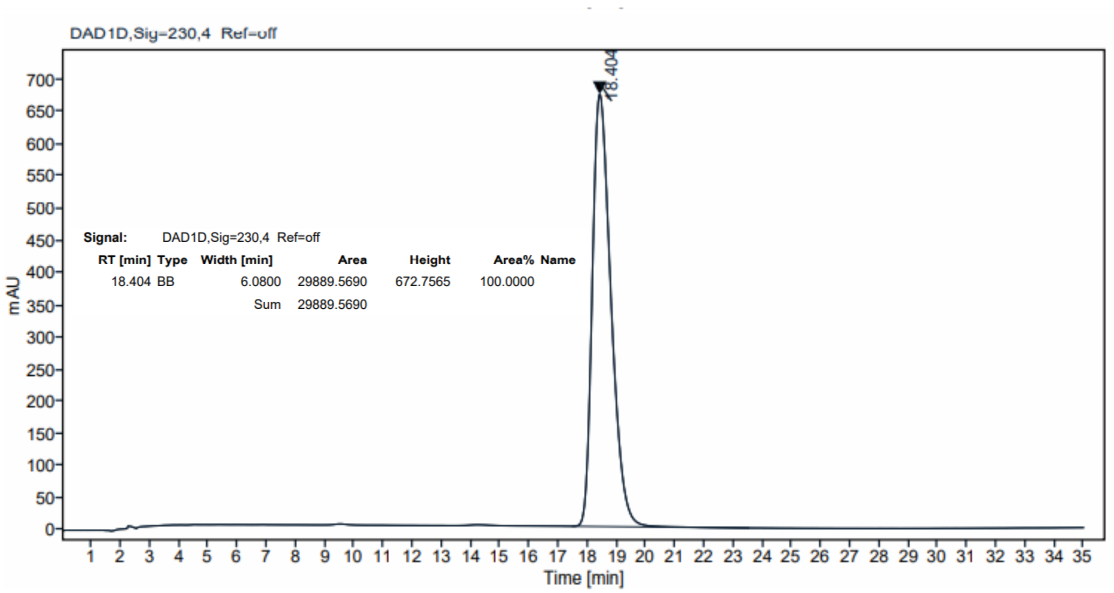

Figure S2: HPLC chromatogram used to determine purity of **6** (HPLC method 2)

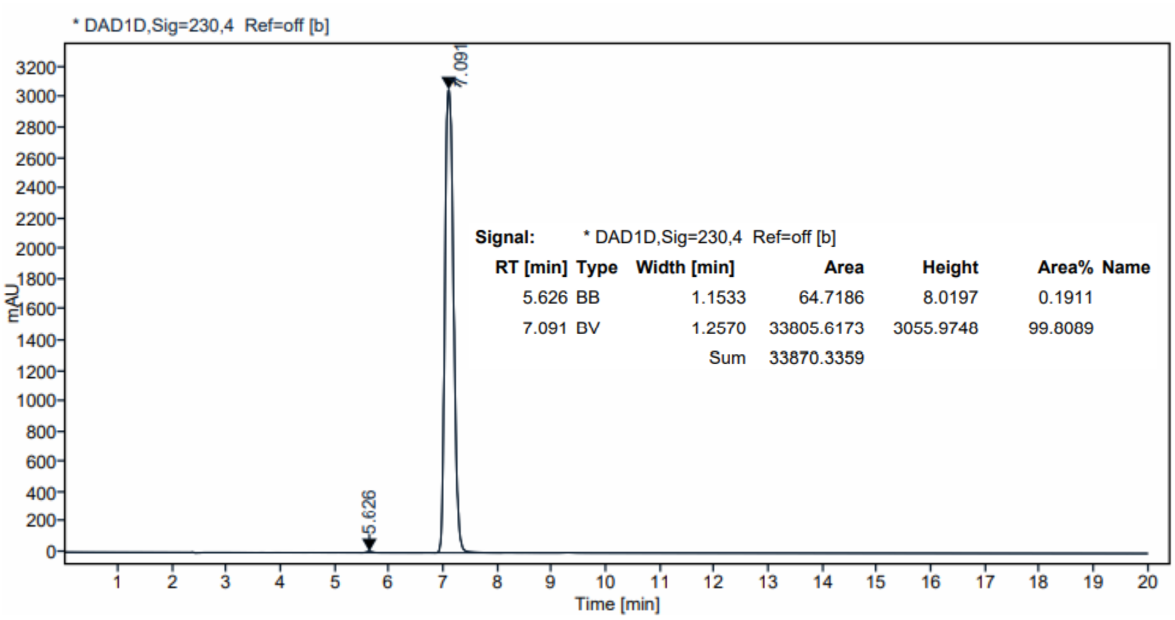

Figure S3: HPLC chromatogram used to determine purity of **7** (HPLC method 1)

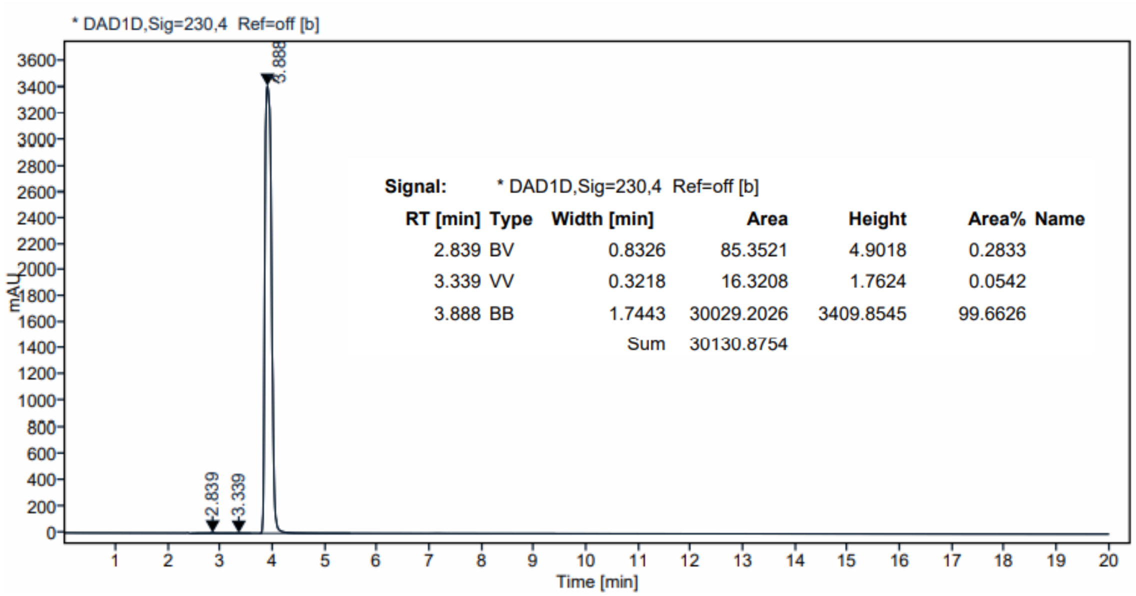

Figure S4: HPLC chromatogram used to determine purity of **8** (HPLC method 1)

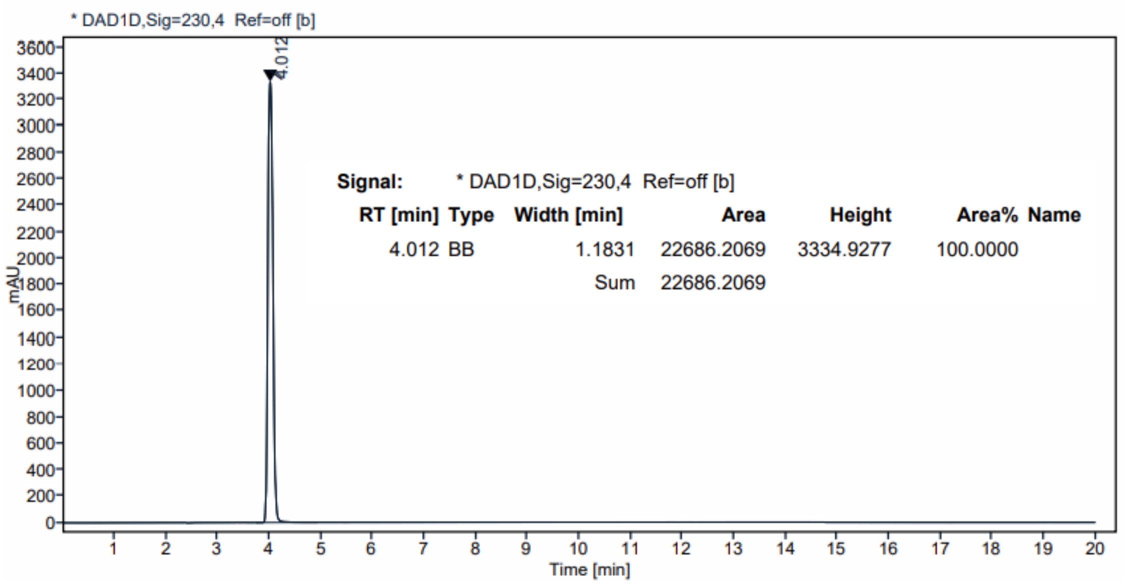

Figure S5: HPLC chromatogram used to determine purity of **9** (HPLC method 1)

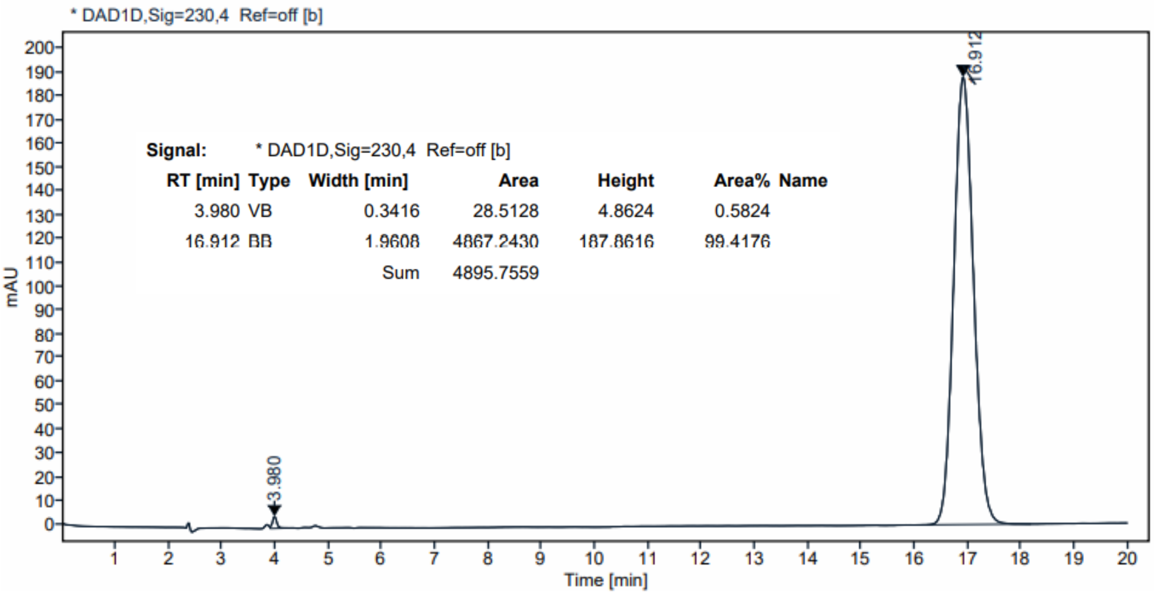

Figure S6: HPLC chromatogram used to determine purity of **10** (HPLC method 1)

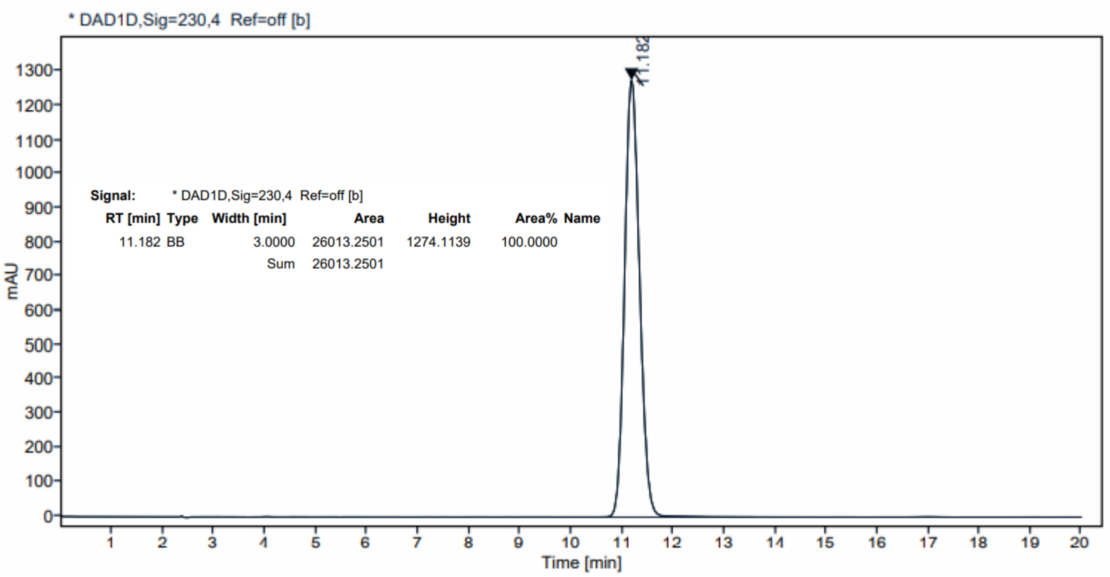

Figure S7: HPLC chromatogram used to determine purity of **12** (HPLC method 1)

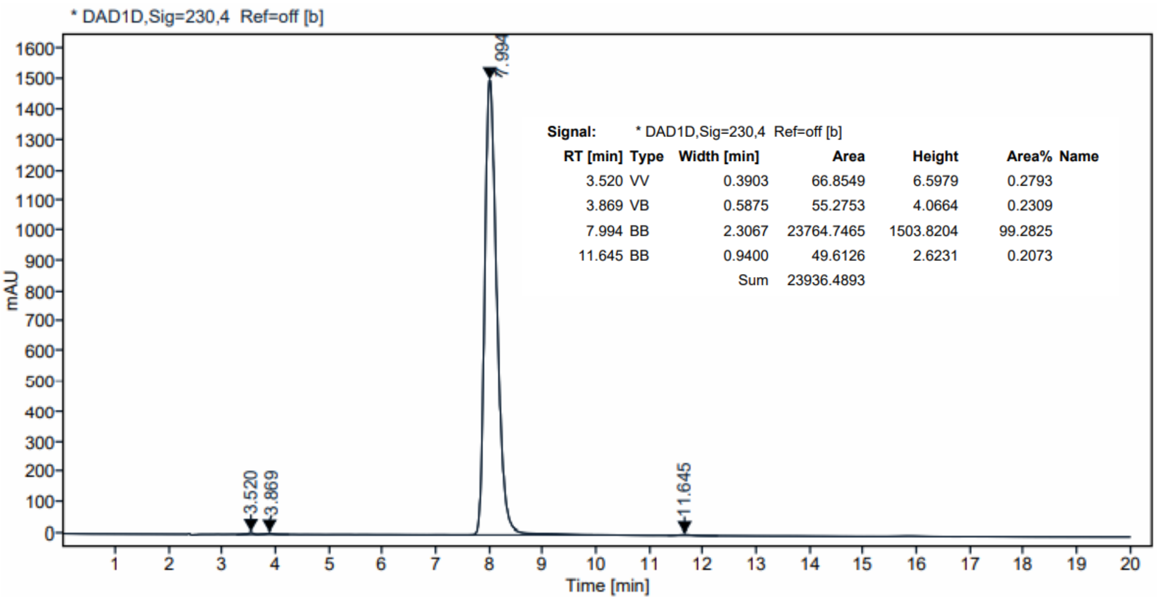

Figure S8: HPLC chromatogram used to determine purity of **13** (HPLC method 1)

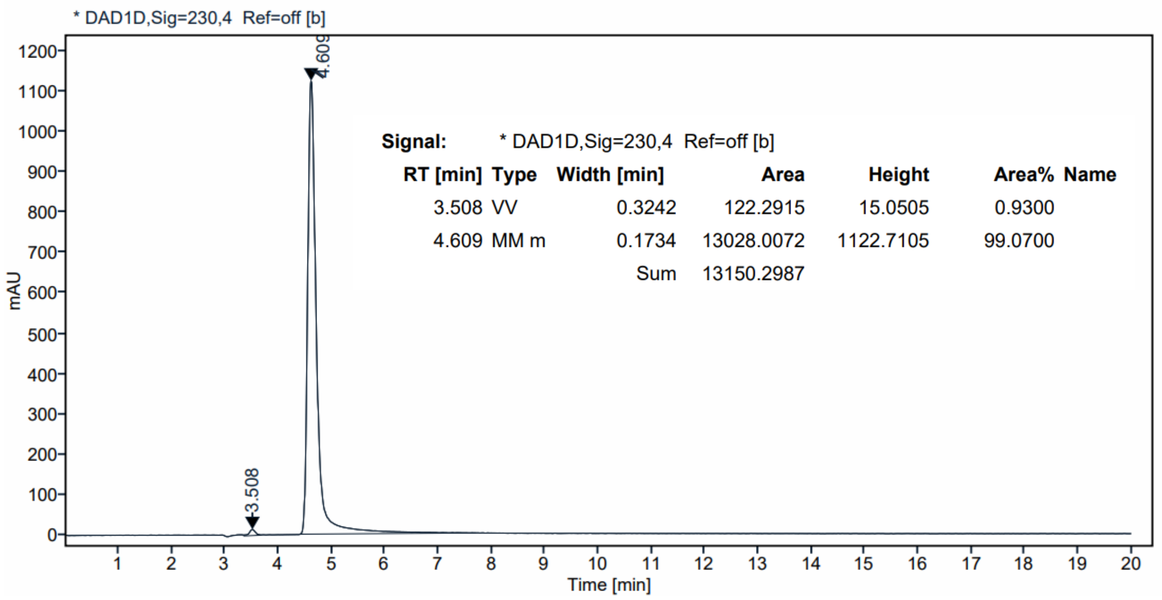

Figure S9: HPLC chromatogram used to determine purity of **15** (HPLC method 2)

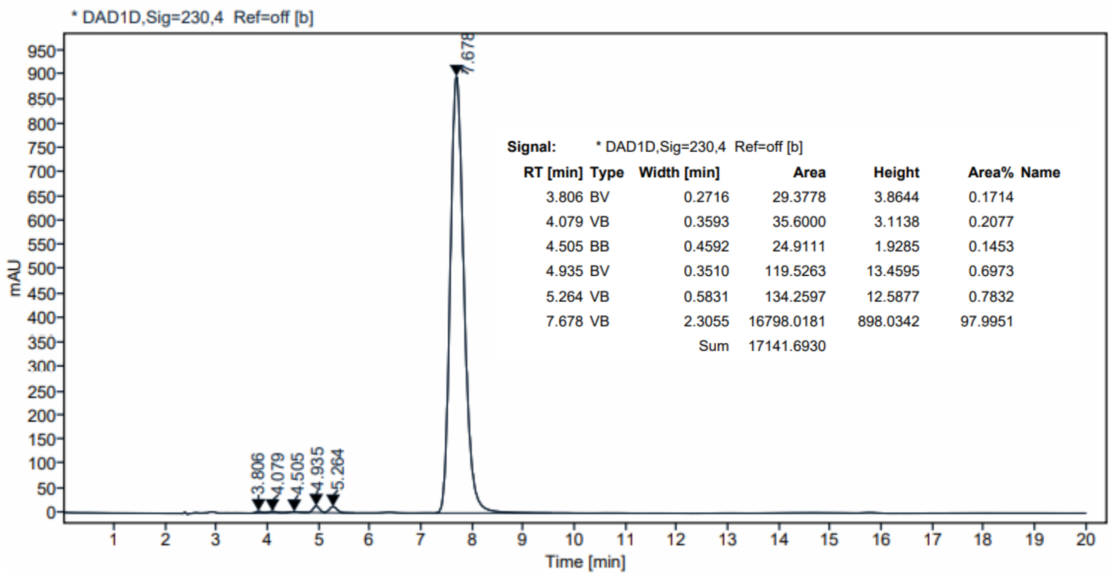

Figure S10: HPLC chromatogram used to determine purity of **17** (HPLC method 1)

8. Experimental Data

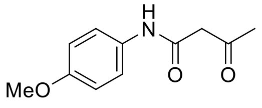

5

***N*-(4-Methoxyphenyl)-3-oxobutanamide (5): ^{1}H NMR (400 MHz, $CDCl_3$) δ** 8.95 (bs, 1H), 7.46 - 7.39 (m, 2H), 6.88 - 6.82 (m, 2H), 3.79 (s, 3H), 3.57 (s, 2H), 2.32 (s, 3H); **^{13}C NMR (100 MHz, $CDCl_3$) δ** 205.9, 163.7, 156.6, 130.7, 122.0, 114.1, 55.4, 50.0, 31.31.0. **mp =** 112-114 °C. **R_f:** 0.30 (30% EtOAc/hexanes). Spectral data matched those previously reported.[2]

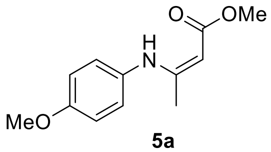

5a

Methyl (Z)-3-((4-methoxyphenyl)amino)but-2-enoate (5a): ^{1}H NMR (400 MHz, $CDCl_3$) δ 10.12 (s, 1H), 7.02 (d, *J* = 8.9 Hz, 2H), 6.85 (d, *J* = 8.9 Hz, 2H), 4.65 (s, 1H), 3.80 (s, 3H), 3.67 (s, 3H), 1.88 (s, 3H); **^{13}C NMR (100 MHz, $CDCl_3$) δ** 170.8, 160.2, 157.5, 132.1, 126.9, 114.2, 84.3, 77.4, 77.0, 76.7, 55.7, 55.4, 50.2, 49.8, 20.1. **R_f:** 0.50 (10% EtOAc/hexanes). Spectral data matched those previously reported.[3]

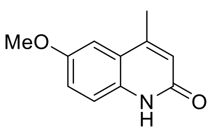

6

6-Methoxy-4-methylquinolin-2(1H)-one (6): ^{1}H NMR (400 MHz, DMSO-d_6) δ 11.45 (s, 1H), 7.23 (d, *J* = 8.8 Hz, 1H), 7.14 (dd, *J* = 8.0, 4.0 Hz, 1H), 7.11 (d, *J* = 2.5 Hz 1H), 6.37 (s, 1H), 3.79 (s, 3H), 2.39 (s, 3H); **^{13}C NMR (125 MHz, DMSO-d_6) δ** 161.6, 154.5, 147.9, 133.5, 121.7, 120.7, 119.4, 117.1, 107.2, 55.9, 19.0. **mp =** 266-269 °C. **R_f:** 0.35 (2% MeOH/CH_2Cl_2). Spectral data matched those previously reported.[4]

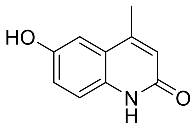

6a

6-hydroxy-4-methylquinolin-2(1H)-one (6a): ^{1}H NMR (500 MHz, DMSO-d$_6$) δ 11.41 (s, 1H), 9.40 (s, 1H), 7.21-7.10 (m, 1H), 7.01 (dd, *J* = 6.4, 2.7 Hz, 2H), 6.36 (d, *J* = 1.4 Hz, 1H), 2.35 (d, *J* = 1.3 Hz, 3H); **^{13}C NMR (125 MHz, DMSO-d$_6$) δ** 161.6, 152.5, 147.5, 132.4, 121.5, 120.9, 120.0, 117.0, 109.0, 19.0. **mp** = 321-324 °C. **R$_f$:** 0.3 (5% MeOH/CH_2Cl_2). Spectral data matched those previously reported.[5]

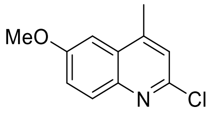

7

2-Chloro-6-methoxy-4-methylquinoline (7): ^{1}H NMR (400 MHz, CDCl$_3$) δ 7.92 (d, *J* = 9.2 Hz, 1H), 7.37 (dd, *J* = 9.2, 2.8 Hz, 1H), 7.21 (s, 1H), 7.15 (d, *J* = 2.7 Hz, 1H), 3.95 (s, 3H), 2.64 (s, 3H).; **^{13}C NMR (100 MHz, CDCl$_3$) δ** 157.8, 147.9, 146.2, 143.4, , 130.5, 127.9, 122.6, 121.8, 102.2, 55.6, 18.7. **m.p.** = 142-145 °C. **R$_f$:** 0.40 (10% EtOAc/hexanes). **HRMS** (ESI-TOF) *m/z*: $[M+H]^+$ calcd for $C_{11}H_{11}ClNO$: 208.0529; found 208.0530.

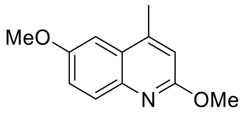

8

2,6-Dimethoxy-4-methylquinoline (8): ^{1}H NMR (400 MHz, CDCl$_3$-d$_6$) δ 7.77 (d, *J* = 9.2 Hz, 1H), 7.28 (dd, *J* = 9.2 and 2.8 Hz, 1H), 7.16 (d, *J* = 2.8 Hz, 1H), 6.74 (s, 1H), 4.03 (s, 3H), 3.92 (s, 3H), 2.58 (s, 3H).; **^{13}C NMR (100 MHz, CDCl$_3$) δ** 161.0, 155.9, 145.5, 141.9, 129.0, 125.9, 120.0, 113.0, 103.4, 55.0, 53.0, 18.8. **mp** = 55-58 °C. **R$_f$:** 0.45 (10% EtOAc/hexanes). Spectral data matched those previously reported.[6]

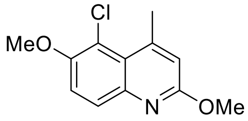

9

5-Chloro-2,6-dimethoxy-4-methylquinoline (9): ^{1}H NMR (400 MHz, CDCl$_3$) δ 7.86 (d, *J* = 8.0 Hz, 1H), 7.37 (d, *J* = 8.7 Hz, 1H), 6.74 (s, 1H), 4.04 (s, 3H), 4.0 (s, 3H), 2.99 (s, 3H).; **^{13}C NMR (100 MHz, CDCl$_3$) δ** 160.8, 152.0, 147.2, 143.5, 127.8, 124.3, 117.8, 116.5, 115.5, 57.1, 53.0, 25. **mp** = 107-110 °C. **R$_f$:** 0.46 (10% EtOAc/hexanes). Spectral data matched those previously reported.[6]

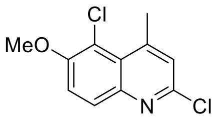

9a

2,5-Dichloro-6-methoxy-4-methylquinoline (9a): ^{1}H NMR (500 MHz, $CDCl_3$) δ 7.95 (d, J = 9.3 Hz, 1H), 7.48 (d, J = 9.2 Hz, 1H), 7.18 (d, J = 1.1 Hz, 1H), 4.03 (s, 3H), 3.04 (d, J = 1.0 Hz, 3H); **^{13}C NMR (125 MHz, $CDCl_3$) δ** 153.9, 148.5, 148.1, 144.3, 129.5, 126.2, 125.7, 117.2, 116.4, 57.0, 25.5. **mp =** 114-118 °C. **R_f:** 0.5 (10% EtOAc/hexanes). **HRMS** (ESI-TOF) *m/z*: $[M+H]^+$ calcd for $C_{11}H_{10}Cl_2NO$: 242.0139; found 242.0144.

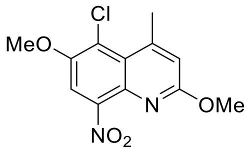

10

5-Chloro-2,6-dimethoxy-4-methyl-8-nitroquinoline (10): ^{1}H NMR (400 MHz, $CDCl_3$) δ 7.62 (s, 1H), 6.83 (s, 1H), 4.02 (s, 3H), 3.98 (s, 3H), 2.99 (s, 3H).; **^{13}C NMR (125 MHz, $CDCl_3$) δ** 161.7, 150.6, 147.6, 146.3, 134.7, 125.4, 122.1, 118.3, 109.4, 57.4, 53.7, 25.6. **mp =** 193-196 °C. **R_f:** 0.35 (10% EtOAc/hexanes). Spectral data matched those previously reported.[6]

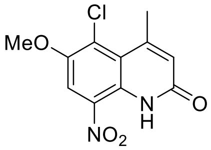

10a

5-chloro-6-methoxy-4-methyl-8-nitroquinolin-2(1H)-one (10a): ^{1}H NMR (400 MHz, $CDCl_3$) δ 6.93 (s, 1H), 6.69 (s, 1H), 4.10 (s, 3H), 2.68 (s, 3H).; **^{13}C NMR (125 MHz, $CDCl_3$) δ** 178.0, 177.9, 166.0, 155.3, 154.2, 150.8, 129.2, 120.9, 115.1, 114.8, 54.7, 54.5, 22.4. **mp =** 205-209 °C. **R_f:** 0.35 (10% EtOAc/hexanes). **HRMS** (ESI-TOF) m/z: $[M+H]^+$ calcd for $C_{11}H_{10}ClN_2O_4$: 269.0329; found 269.0345.

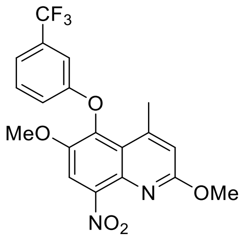

12

2,6-Dimethoxy-4-methyl-8-nitro-5-(3-(trifluoromethyl)phenoxy)quinoline (12): ^{1}H NMR (400 MHz, CDCl$_3$) δ 7.76 (s, 1H), 7.32 (d, *J* = 7.9 Hz, 1H), 7.33 - 7.29 (m, 1H), 7.08 (s, 1H), 6.92 (dd, *J* = 8.1 and 2.5 Hz, 1H), 6.77 (s, 1H), 4.01 (s, 3H), 3.81 (s, 3H), 2.63 (s, 3H).; **^{13}C NMR (125 MHz, CDCl$_3$) δ** 162.4, 157.9, 146.5, 146.0, 144.5, 140.8, 134.9, 132.0 (q, J_{C-F} = 26.4 Hz), 130.4, 129.9, 124.7, 122.6, 122.1, 121.9, 120.4, 119.2 (q, J_{C-F} = 4.0 Hz), 118.2, 117.3, 112.2 (q, J_{C-F} = 3.9 Hz), 111.3, 57.0, 53.8, 23.1. **^{19}F NMR (376 MHz, CDCl$_3$)** δ -62.64. **mp =** 192-195 °C. **R$_f$:** 0.40 (20% EtOAc/hexanes). **HRMS** (ESI-TOF) *m/z*: $[M+H]^+$ calcd for $C_{19}H_{16}F_3N_2O_5$: 409.1011; found 409.1041.

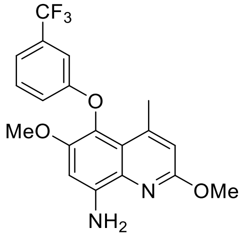

13

2,6-Dimethoxy-4-methyl-5-(3-(trifluoromethyl)phenoxy)quinolin-8-amine (13): ^{1}H NMR (400 MHz, CDCl$_3$) δ 7.33 (t, *J* = 7.8 Hz, 1H), 7.22 (d, *J* = 7.3 Hz, 1H), 7.04 (s, 1H), 6.93 (dd, *J* = 8.4 and 2.6 Hz 1H), 6.78 (s, 1H), 6.65 (s, 1H), 4.86 (brs, 2 H), 4.02 (s, 3H), 3.76 (s, 3H), 2.55 (s, 3H).; **^{13}C NMR (125 MHz, CDCl$_3$) δ** 159.7, 159.6, 148.5, 146.1, 141.4, 131.9 (q, J_{C-F} = 26.4 Hz), 131.1, 130.0, 128.4, 128.0, 125.4, 122.6, 120.8, 119.9, 118.2, 118.0 (q, J_{C-F} = 3.7 Hz), 115.6, 111.9 (q, J_{C-F} = 3.9 Hz), 56.5, 52.9, 23.03. **^{19}F NMR (376 MHz, CDCl$_3$)** δ -62.56. **mp =** 113-115 °C. **R$_f$:** 0.30 (30% EtOAc/hexanes). **HRMS** (ESI-TOF) *m/z*: $[M+H]^+$ calcd for $C_{19}H_{18}F_3N_2O_3$: 379.1270; found 379.1281.

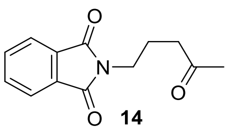

14

2-(4-Oxopentyl)isoindoline-1,3-dione (14): ^{1}H NMR (400 MHz, CDCl$_3$) δ 7.86 - 7.78 (m, 2H), 7.74 - 7.64 (m, 2H), 3.69 (t, *J* = 6.6 Hz, 2H), 2.49 (t, *J* = 7.4 Hz, 1H), 2.13 (s, 1H), 1.95 (q, *J* = 6.8 Hz, 2H).; **^{13}C**

NMR (100 MHz, $CDCl_3$) δ 207.4, 168.4, 134.0, 132.0, 40.5, 37.2, 29.9. 22.7. **R_f:** 0.35 (40% EtOAc/hexanes). Spectral data matched those previously reported.[7]

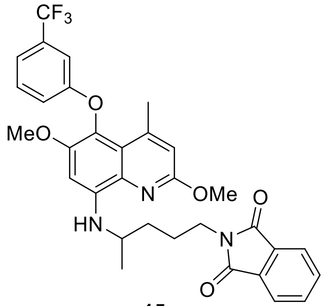

15

2-(4-((2,6-Dimethoxy-4-methyl-5-(3-(trifluoromethyl)phenoxy)quinolin-8-yl)amino)pentyl) isoindoline-1,3-dione (15): ^{1}H NMR (400 MHz, $CDCl_3$) δ δ 7.80 (dd, J = 5.4, 3.0 Hz, 2H), 7.75 - 7.65 (m, 2H), 7.32 (t, J = 8.0 Hz, 1H), 7.20 (ddd, J = 7.7, 1.7, 0.9 Hz, 1H), 7.07 (t, J = 2.1 Hz, 1H), 6.90 (dd, J = 8.2, 2.5 Hz, 1H), 6.62 (d, J = 1.1 Hz, 1H), 6.49 (s, 1H), 5.80 (s, 1H), 3.99 (s, 3H), 3.77 (s, 3H), 3.76 – 3.64 (m, 6H), 2.52 (s, 3H), 2.00 -1.64 (m, 4H), 1.33 (d, J = 6.3 Hz, 3H).; **^{13}C NMR (100 MHz, $CDCl_3$) δ** 168.4, 159.7, 159.4, 148.9, 146.2, 141.8, 139.4, 134.0, 132.0, 131.8, (q, J_{C-F} = 32.5 Hz), 130.9, 129.9, 128.0, 126.8, 125.3, 123.1, 122.6, 120.6, 119.9, 118.1, 117.9 (q, J_{C-F} = 3.7 Hz), 115.2, 112.1, (q, J_{C-F} = 3.9 Hz), 94.9, 56.9, 52.9, 48.2, 38.0, 34.0, 25.2, 23.1, 20.8. **^{19}F NMR (376 MHz, $CDCl_3$)** δ -62.52.; **mp =** 61-65 °C. **R_f:** 0.40 (30% EtOAc/hexanes). **HRMS** (ESI-TOF) *m/z*: $[M+Na]^+$ calcd for $C_{32}H_{30}F_3N_3NaO_5$: 616.2035; found 616.2007.

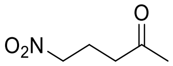

16

5-Nitropentan-2-one (16): ^{1}H NMR (400 MHz, $CDCl_3$) δ 4.42 (t, J = 6.6 Hz, 2H), 2.59 (t, J = 6.8 Hz, 2H), 2.23 (p, J = 6.7 Hz, 2H), 2.16 (s, 3H).; ^{13}C NMR (**101 MHz, $CDCl_3$**) δ 206.5, 39.2, 29.9, 29.6, 21.1.; Spectral data matched those previously reported. **R_f:** 0.45 (30% EtOAc/hexanes). Spectral data matched those previously reported.[8]

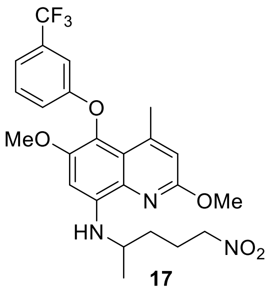

2,6-Dimethoxy-4-methyl-N-(5-nitropentan-2-yl)-5-(3-(trifluoromethyl)phenoxy)quinolin-8-amine (17): **^{1}H NMR (400 MHz, CDCl$_3$) δ** 7.34 (t, J = 8.0 Hz, 1H), 7.22 (d, J = 7.6 Hz, 1H), 7.06 (t, J = 2.1 Hz, 1H), 6.93 (dd, J = 8.3, 2.6 Hz, 1H), 6.66 (d, J = 1.1 Hz, 1H), 6.49 (s, 1H), 5.81 (s, 1H), 4.53 - 4.40 (m, 2H), 4.01 (s, 3H), 3.80 (s, 3H), 3.75 - 3.68 (m, 1H), 2.55 (d, J = 1.1 Hz, 3H), 2.35 - 2.09 (m, 2H), 1.78 (dtd, J = 8.5, 6.5, 1.4 Hz, 2H), 1.37 (d, J = 6.3 Hz, 3H). **^{13}C NMR (101 MHz, CDCl$_3$) δ** 159.7, 159.6, 148.9, 146.4, 141.6, 131.8 (q, J_{C-F} = 32.0 Hz), 131.0, 130.0, 128.0, 127.2, 125.3, 122.6, 120.7, 119.9, 118.2, 117.9, (q, J_{C-F} = 4 Hz), 115.4, 112.0 (q, J_{C-F} = 4 Hz), 95.0, 75.5, 57.0, 52.9, 48.0, 33.6, 24.2, 23.1, 20.9. **^{19}F NMR (376 MHz, CDCl$_3$)** δ -62.55. **mp =** 83-85 °C. **R$_f$:** 0.40 (20% EtOAc/hexanes). **HRMS** (ESI-TOF) *m/z*: $[M+H]^+$ calcd for $C_{24}H_{27}F_3N_3O_5$: 494.1902; found 494.1922.

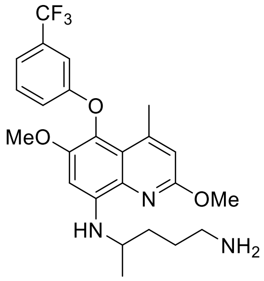

***N*^{4}-(2,6-Dimethoxy-4-methyl-5-(3-(trifluoromethyl)phenoxy)quinolin-8-yl)pentane-1,4-diamine (13):** **^{1}H NMR (400 MHz, CDCl$_3$) δ** 7.32 (t, J = 8.2 Hz, 1H), 7.24 - 7.17 (m, 1H), 7.07 (t, J = 2.1 Hz, 1H), 6.93 (dd, J = 8.3, 2.6 Hz, 1H), 6.64 (d, J = 1.1 Hz, 1H), 6.50 (s, 1H), 5.83 (d, J = 8.1 Hz, 1H), 3.99 (s, 3H), 3.78 (s, 3H), 3.71 – 3.60 (m, 1H), 2.77 (t, J = 6.5 Hz, 2H), 2.54 (d, J = 1.1 Hz, 3H), 1.84 - 1.72 (m, 1H), 1.72 - 1.55 (m, 3H), 1.43 (s, 1H), 1.34 (d, J = 6.3 Hz, 3H).; **^{13}C NMR (100 MHz, CDCl$_3$) δ** 159.8, 159.4, 148.9, 146.3, 142.0, 131.8 (q, J_{C-F} = 32.0 Hz), 130.9, 128.0, 126.8, 125.3, 122.6, 120.6, 119.9 (q, J_{C-F} = 4.0 Hz), 115.2, 112.0 (q, J_{C-F} = 4 Hz), 94.8, 56.9, 52.9, 48.4, 42.3, 34.4, 30.3 23.1, 20.8. **^{19}F NMR (376 MHz, CDCl$_3$)** δ -62.57. **R$_f$:** 0.25 (5% MeOH/CH_2Cl_2). Spectral data matched those previously reported.[9]

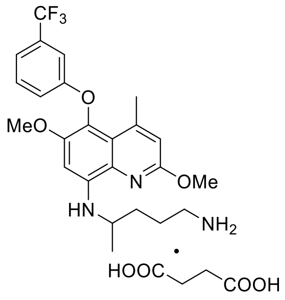

2

Tafenoquine succinate (2): ^{1}**H NMR (400 MHz, DMSO-d$_6$) δ** 7.49 (t, *J* = 8.0 Hz, 1H), 7.35 - 7.28 (m, 1H), 7.03 - 6.94 (m, 2H), 6.78 (d, *J* = 1.1 Hz, 1H), 6.66 (s, 1H), 5.82 (d, *J* = 8.9 Hz, 1H), 3.94 (s, 3H), 3.78 (s, 1H), 3.74 (s, 3H), 2.84 - 2.76 (m, 2H), 2.45 (d, *J* = 1.0 Hz, 3H), 2.22 (s, 4H), 1.74 - 1.59 (m, 5H), 1.25 (d, *J* = 6.2 Hz, 3H). ^{13}**C NMR (125 MHz, DMSO-d$_6$) δ** 175.9, 159.9, 159.2, 149.2, 146.0, 142.2, 131.50, 130.8 (q, J_{C-F} = 31.4 Hz), 130.3, 127.6, 125.6, 125.5, 123.3, 121.1, 120.3, 119.1, 118.3 (q, J_{C-F} = 4.0 Hz), 115.4, 111.6 (q, JC-F = 4.0 Hz), 95.0, 56.9, 53.3, 47.5, 39.6, 33.7, 32.9, 24.8, 22.9, 20.9.; ^{19}**F NMR (471 MHz, DMSO-*d*$_6$)** δ -61.11. **HRMS** (ESI-TOF) *m/z*: $[M+H]^+$ calcd for $C_{24}H_{29}F_3N_3O_3$: 464.2161; found 464.2136. **mp =** 146–148 °C. **R$_f$:** N/A for succinate salt.

9. References

1. B. H. Lipshutz, S. Ghorai, A. R. Abela, R. Moser, T. Nishikata, C. Duplais, A. Krasovskiy, R. D. Gaston and R. C. Gadwood, *J. Org. Chem.* 2011, **76**, 4379-4391.
2. A. K. Kabi, R. Gujjarappa. N. Vodnala, D. Kaldhi, U. Tyagi, K. Mukherjee and C. C. Malakar, *Tetrahedron Lett.* **2020**, *61*, 152535-152541.
3. S. Würtz, S. Rakshit, J. J. Neumann, T. Dröge and F. Glorius, *Angew. Chem., Int. Ed.* 2008, **47**,7230-7233.
4. Z. Zhang, L-L. Liao, Si-S. Yan, L. Wang, Y.-Q. He, J.-H. Ye, J. Li, Y.-G. Zhi, and Yu, D-G. *Angew. Chem., Int. Ed.* **2016**, *55*, 7068-7072.
5. R. Manikandan and M. Jeganmohan, *Org. Lett.* 2014, **16**, 3568-3571.
6. H. Shiraki, M. P. Kozar, V. Melendez, T. H. Hudson, C. Ohrt, A. J. Magill and A. J. Lin, *J. Med. Chem.* **2011**, *54*, 131-142.
7. X. Li, X. Che,, G.-H. Chen, J. Zhang, J.- L. Yan, Y.-F. Zhang, L-S. Zhang, C.-P. Hsu, Y. Q. Gao and Z.-J. Shi, *Org. Lett.* **2016**, *18*, 1234-1337.
8. X. Han, L. Dong, C. Geng and P. Jiao, *Org. Lett.* **2015**, *17*, 3194-3197.
9. A. K. Das, N. Hayashi, Y. Shiraishi and T. Hirai, *RSC Adv.,* **2017**, *7*, 30453-30458.
10. F. Roschangar, R. A. Sheldon and C. H. Senanayake, Green Chem., 2015, 17, 752–768.

10. ¹H, ¹³C NMR Spectra of synthesized products

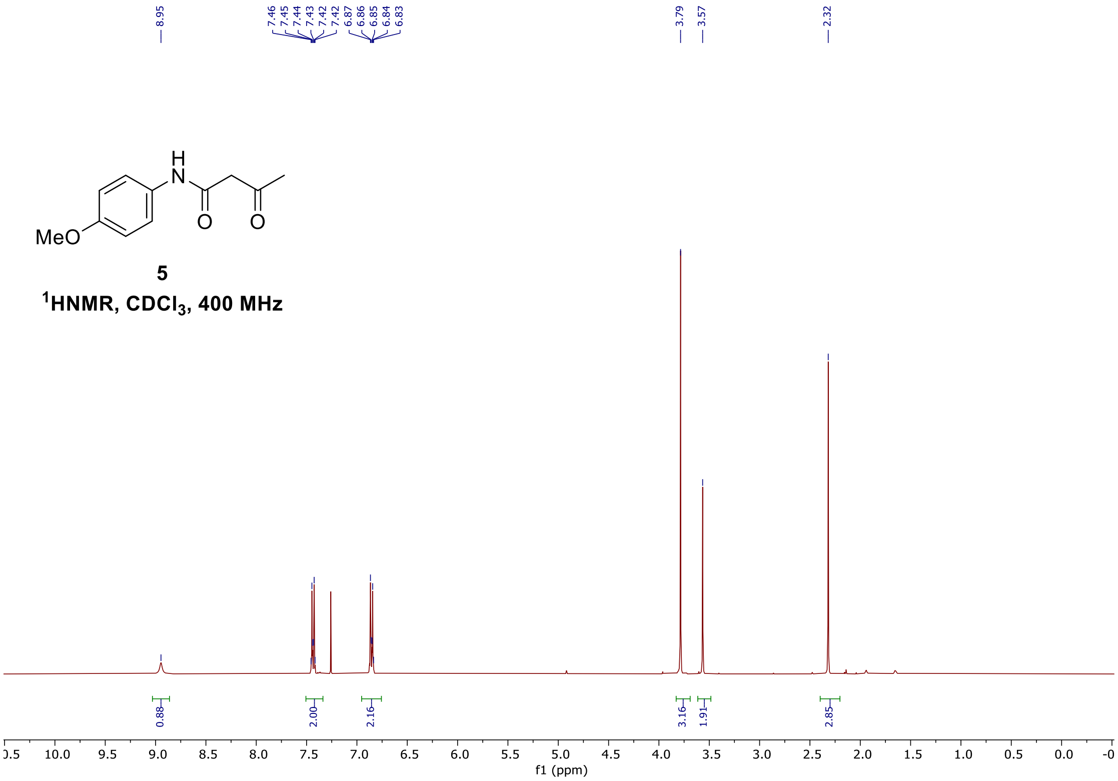

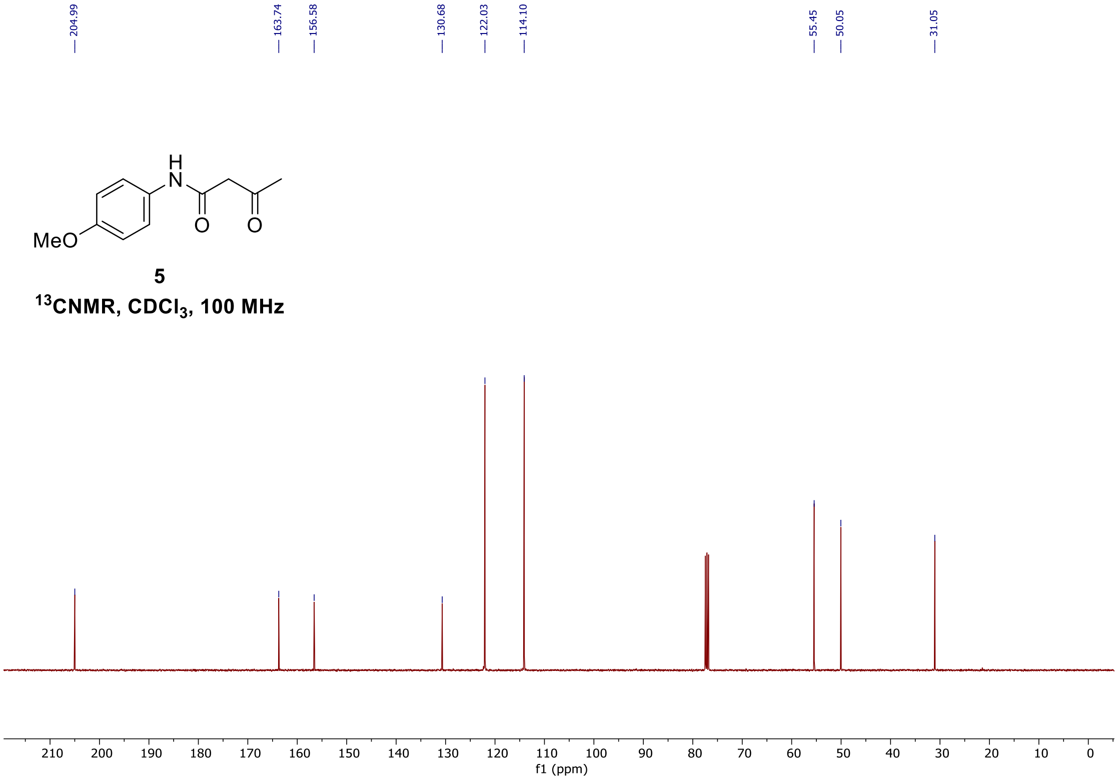

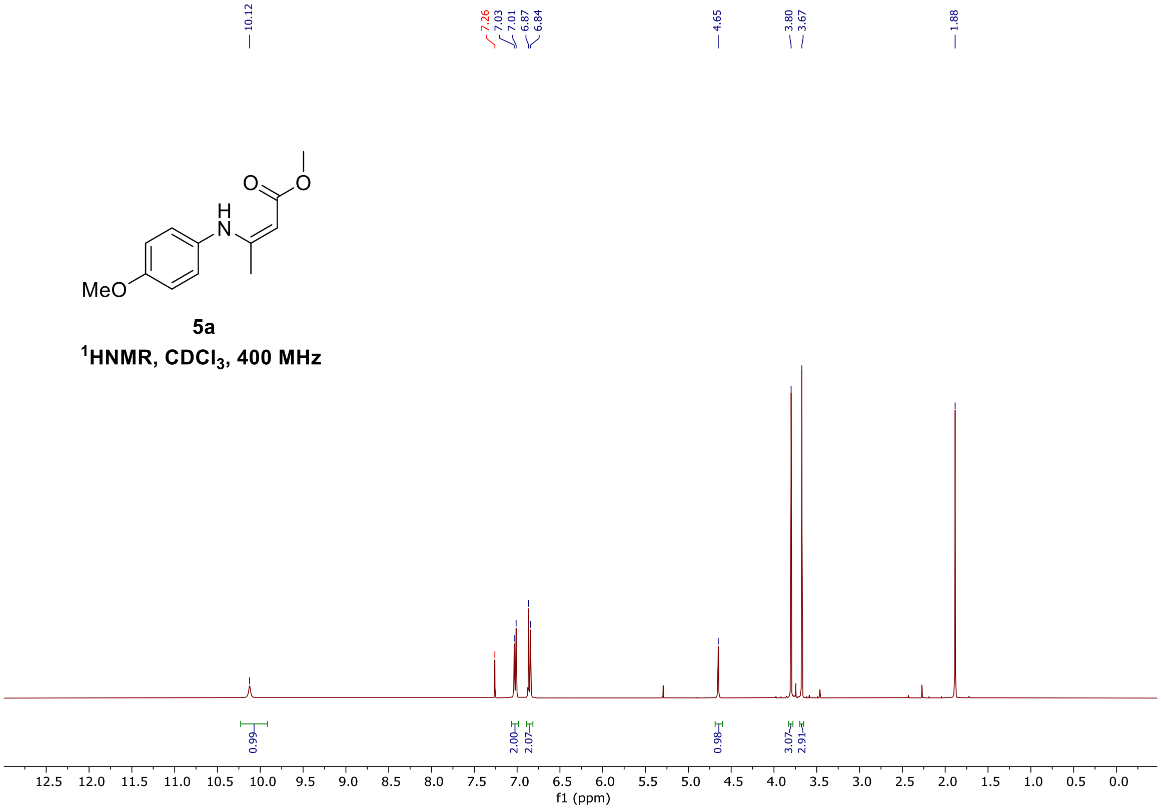
10.12
7.26
7.03
7.01
6.87
6.84
4.65
3.80
3.67
1.88
MeO
H
N
O
O
5a
¹HNMR, CDCl₃, 400 MHz
0.99
2.00
2.07
0.98
3.07
2.91
12.5 12.0 11.5 11.0 10.5 10.0 9.5 9.0 8.5 8.0 7.5 7.0 6.5 6.0 5.5 5.0 4.5 4.0 3.5 3.0 2.5 2.0 1.5 1.0 0.5 0.0
f1 (ppm)

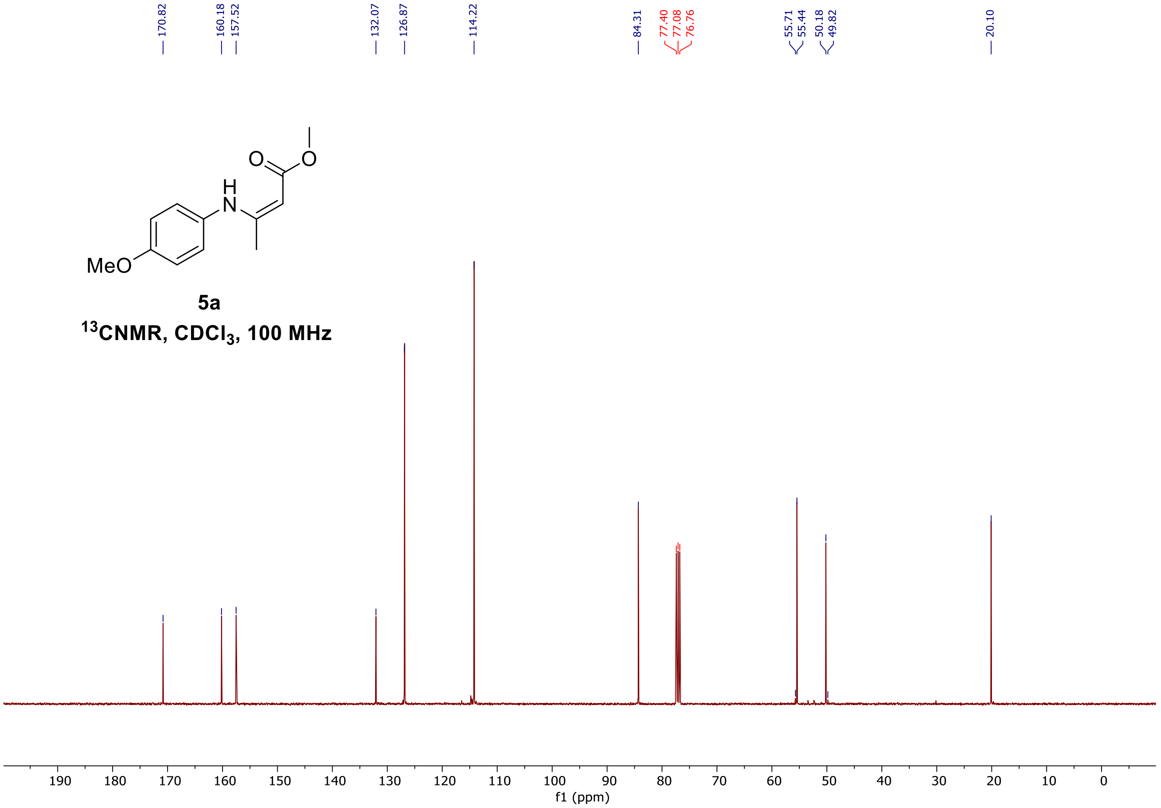
170.82
160.18
157.52
132.07
126.87
114.22
84.31
77.40
77.08
76.76
55.71
55.44
50.18
49.82
20.10
MeO
H
N
O
O
5a
¹³CNMR, CDCl₃, 100 MHz
190 180 170 160 150 140 130 120 110 100 90 80 70 60 50 40 30 20 10 0
f1 (ppm)

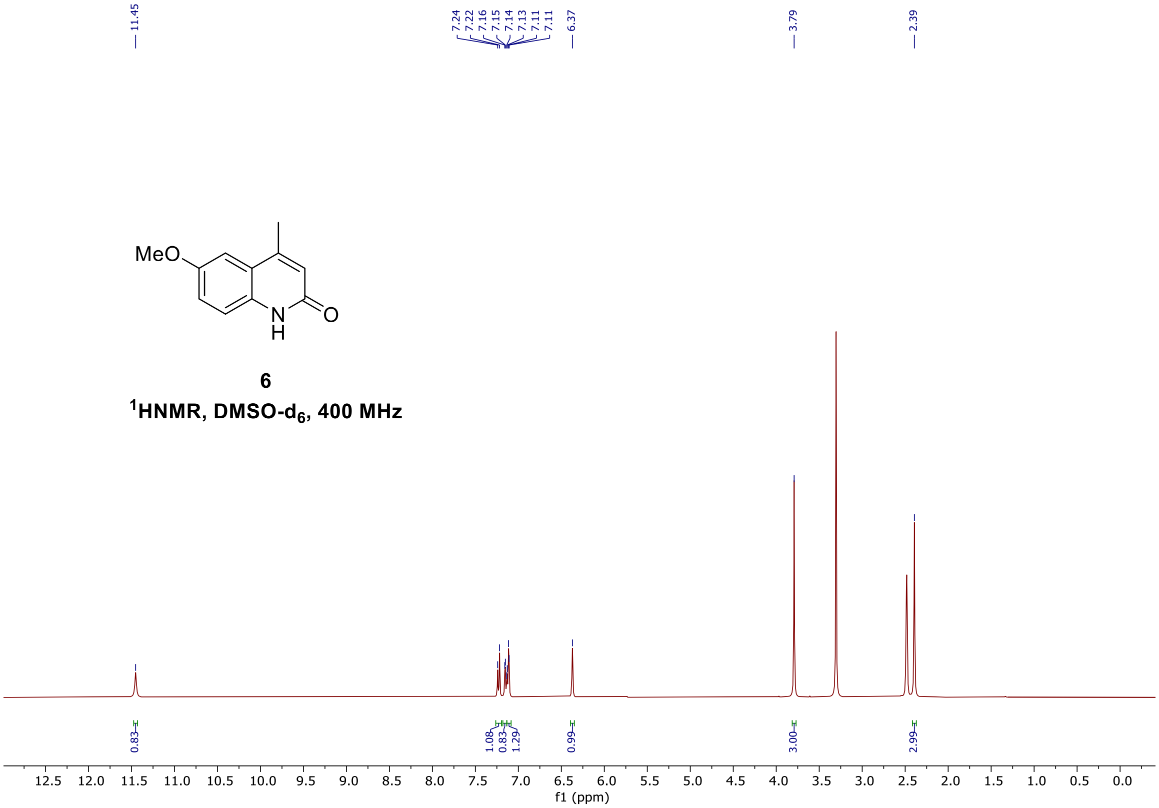
11.45
7.24
7.22
7.16
7.15
7.14
7.13
7.11
7.11
6.37
3.79
2.39
MeO
N
H
O
6
1HNMR, DMSO-d6, 400 MHz
0.83
1.08
0.83
1.29
0.99
3.00
2.99
12.5 12.0 11.5 11.0 10.5 10.0 9.5 9.0 8.5 8.0 7.5 7.0 6.5 6.0 5.5 5.0 4.5 4.0 3.5 3.0 2.5 2.0 1.5 1.0 0.5 0.0
f1 (ppm)

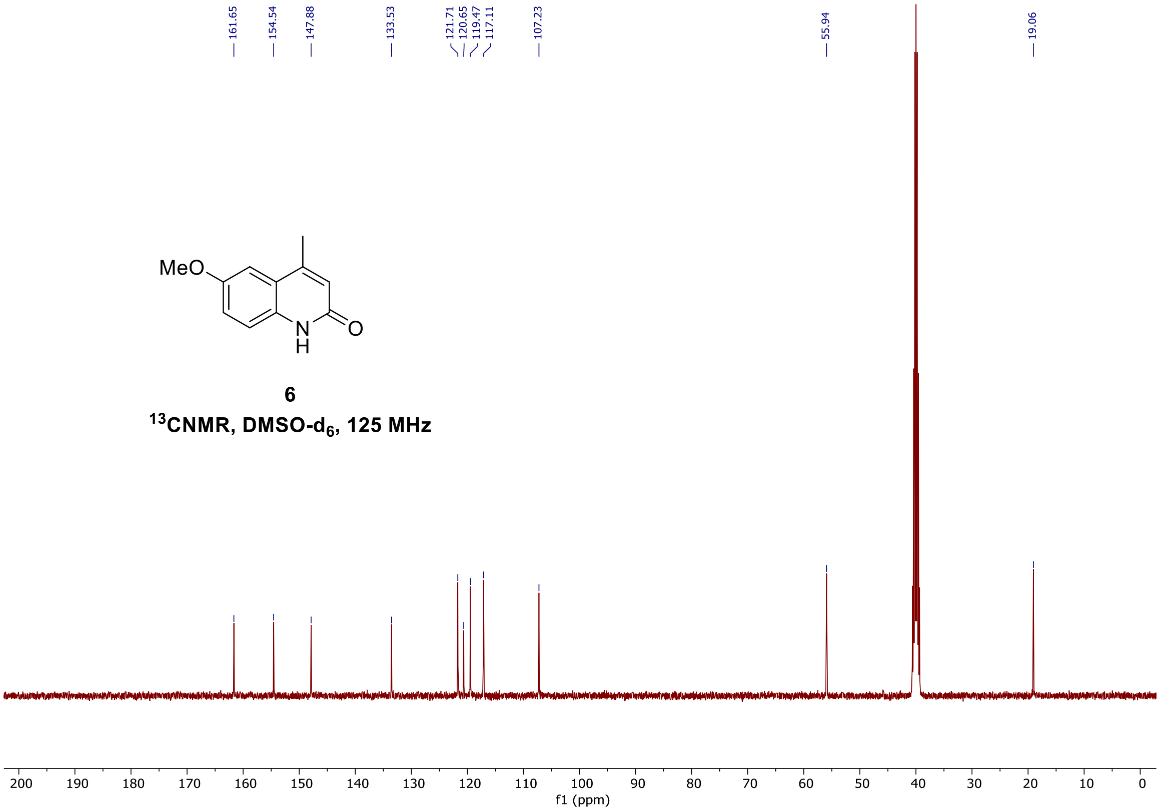
161.65
154.54
147.88
133.53
121.71
120.65
119.47
117.11
107.23
55.94
19.06
MeO
N
H
O
6
13CNMR, DMSO-d6, 125 MHz
200 190 180 170 160 150 140 130 120 110 100 90 80 70 60 50 40 30 20 10 0
f1 (ppm)

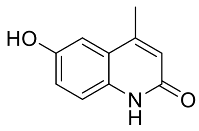

6a

^{1}HNMR, DMSO-d_6, 500 MHz

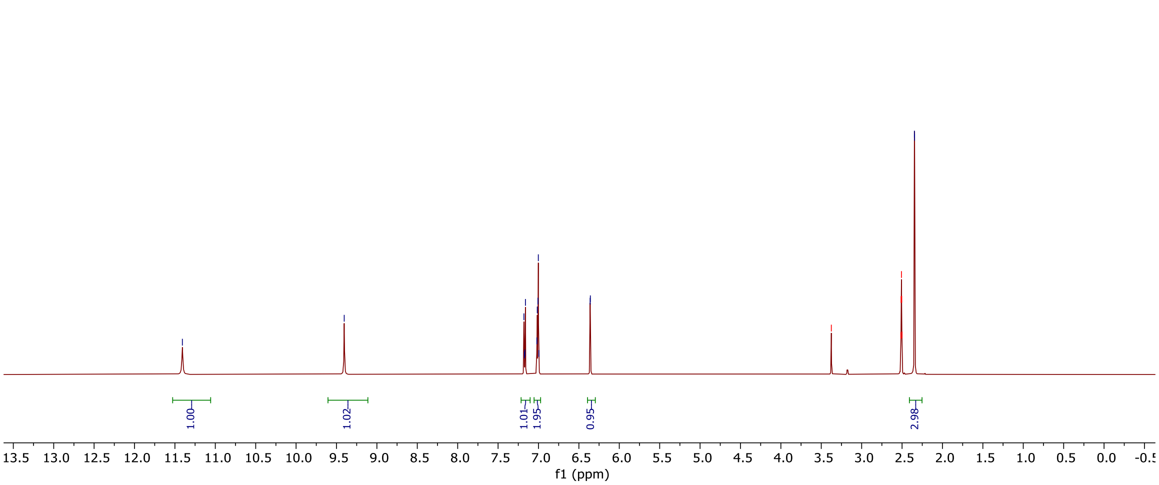

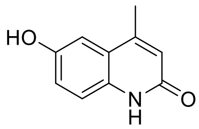

6a

^{13}CNMR, DMSO-d_6, 125 MHz

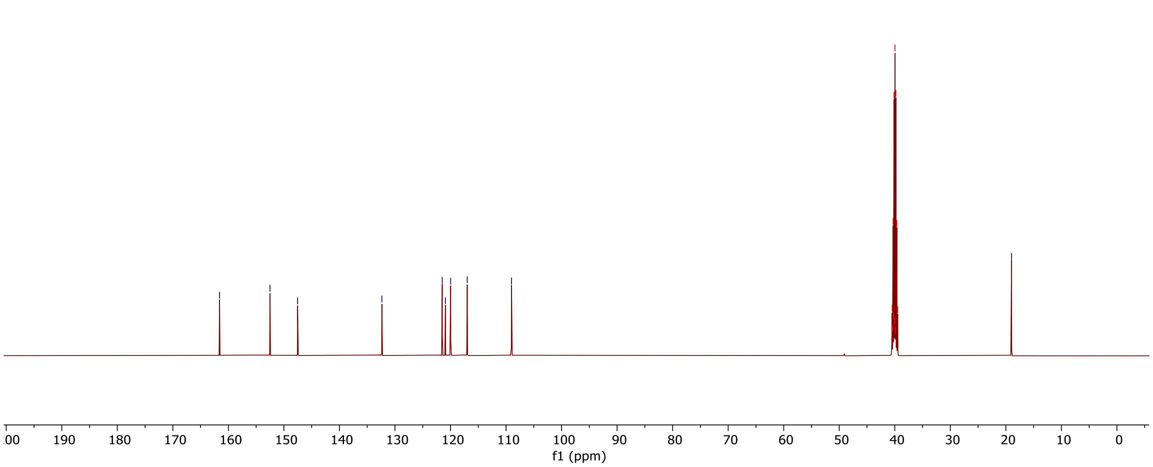

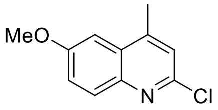

7

^{1}HNMR, $CDCl_3$, 400 MHz

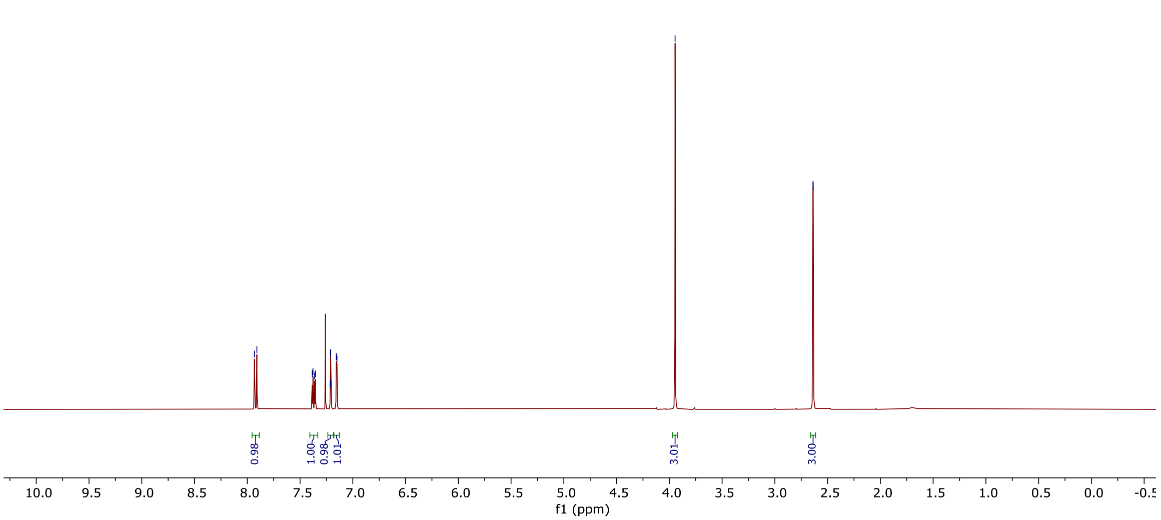

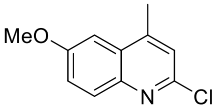

7

^{13}CNMR, $CDCl_3$, 100 MHz

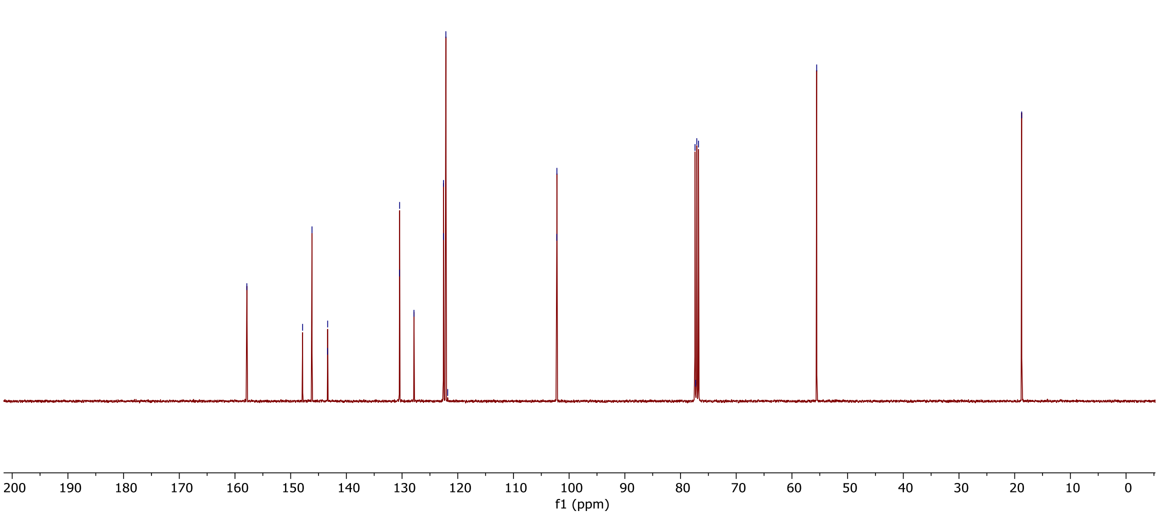

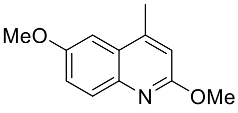

8

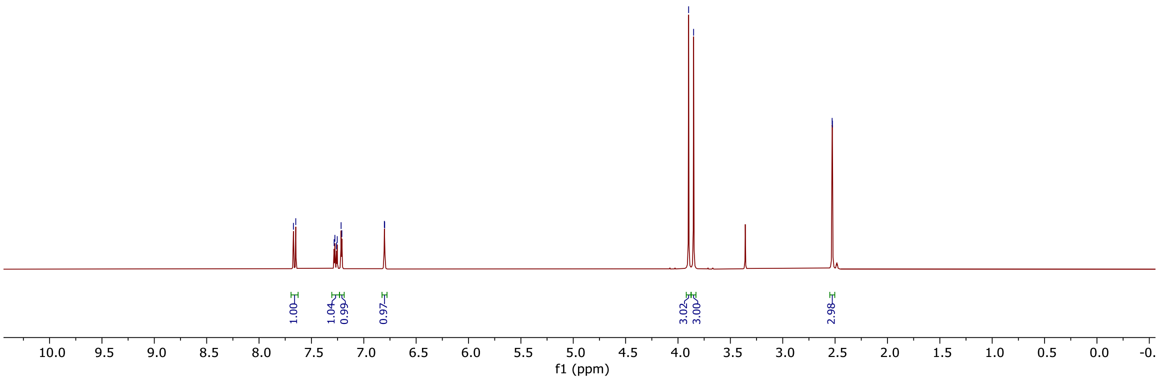

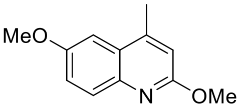

8

^{13}CNMR, $CDCl_3$, 100 MHz

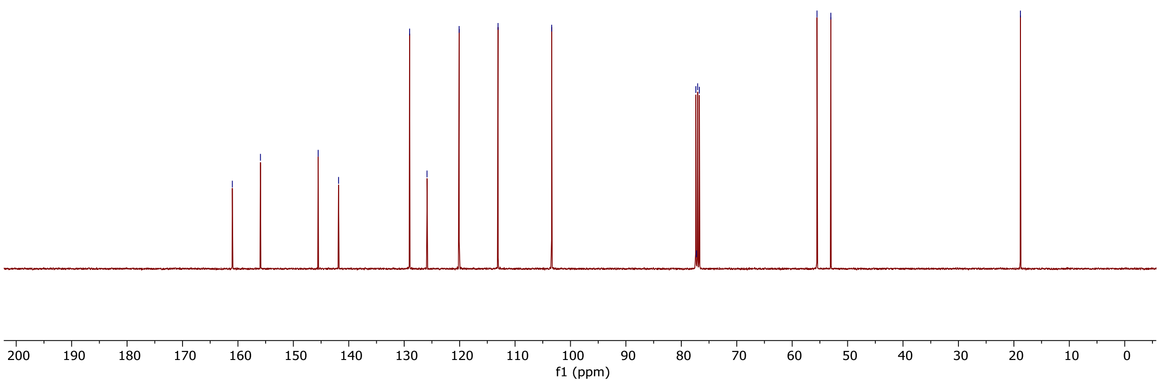

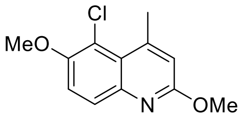

9

^{1}HNMR, $CDCl_3$, 400 MHz

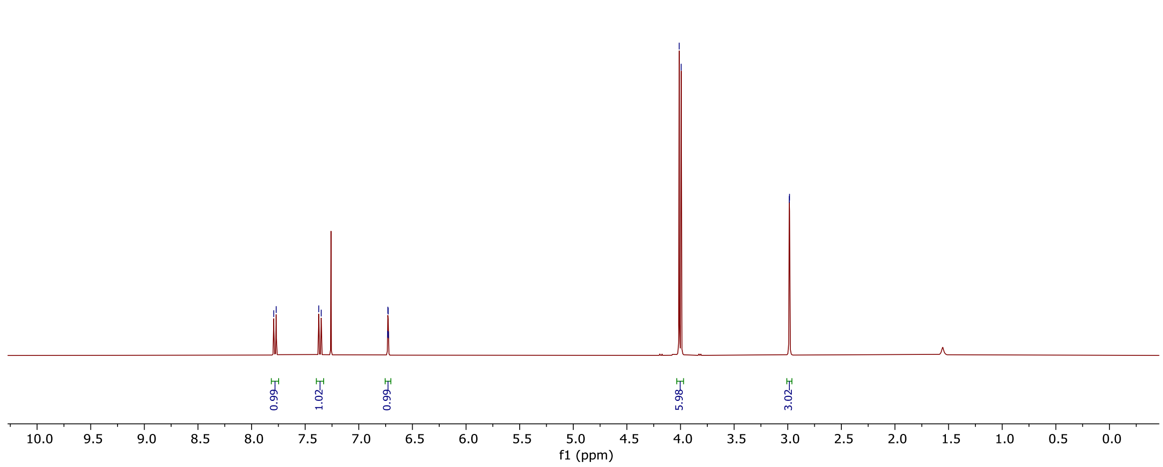

9

^{13}CNMR, $CDCl_3$, 100 MHz

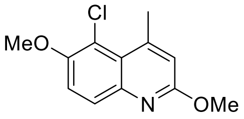

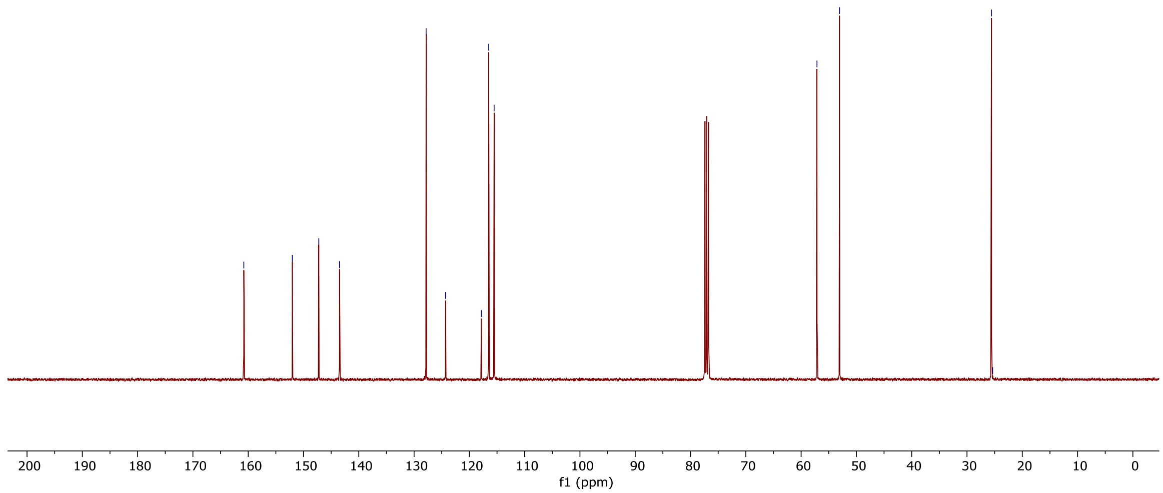

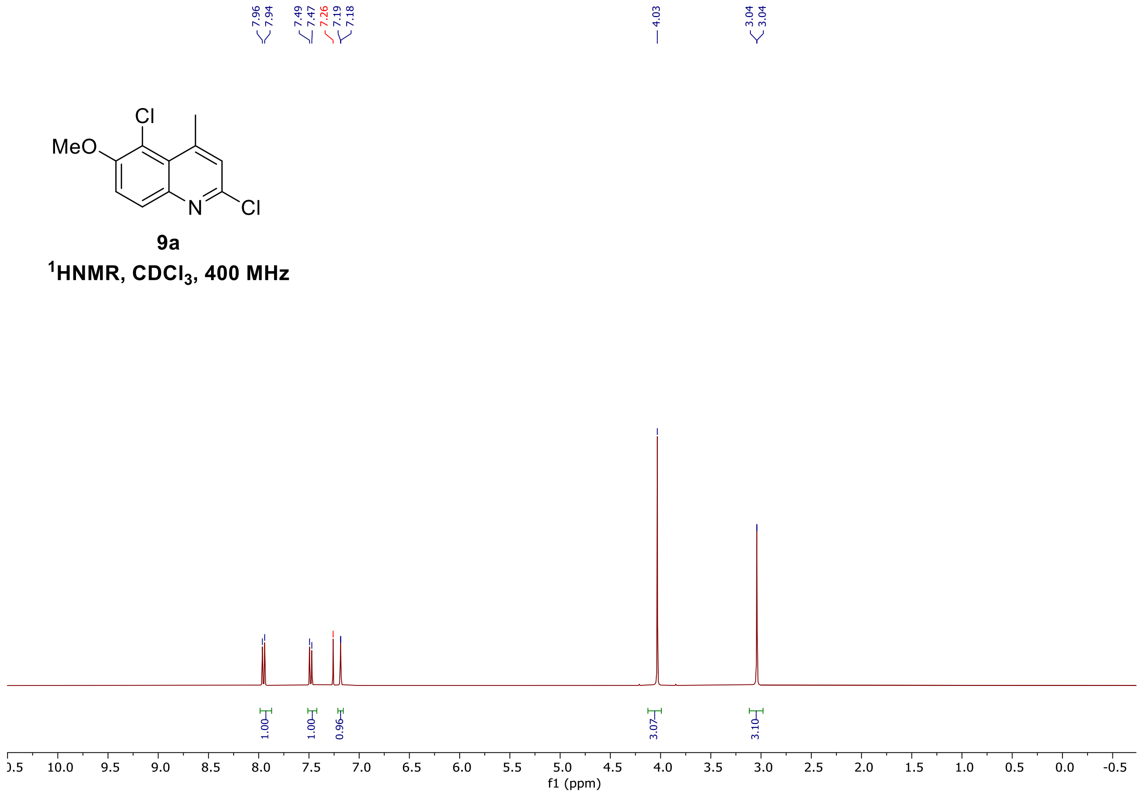
7.96
7.94
7.49
7.47
7.26
7.19
7.18
4.03
3.04
3.04
Cl
MeO
N
Cl
9a
1HNMR, CDCl3, 400 MHz
1.00
1.00
0.96
3.07
3.10
f1 (ppm)

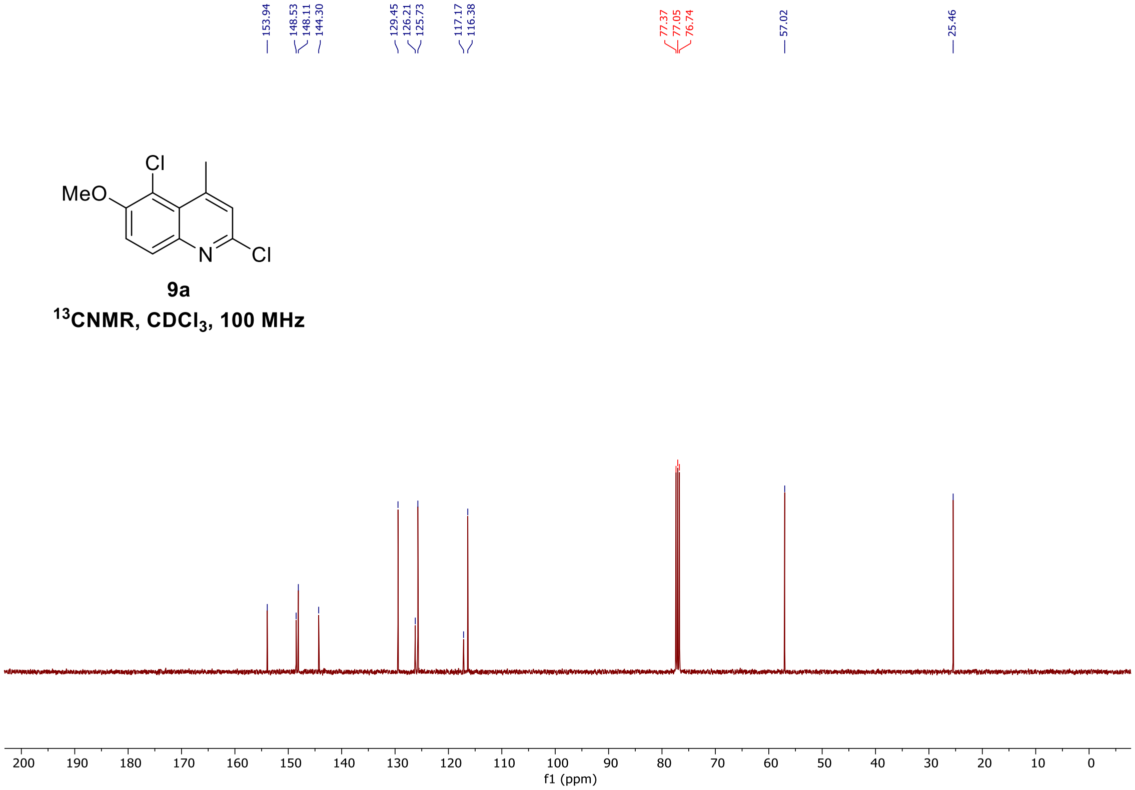
153.94
148.53
148.11
144.30
129.45
126.21
125.73
117.17
116.38
77.37
77.05
76.74
57.02
25.46
Cl
MeO
N
Cl
9a
13CNMR, CDCl3, 100 MHz
f1 (ppm)

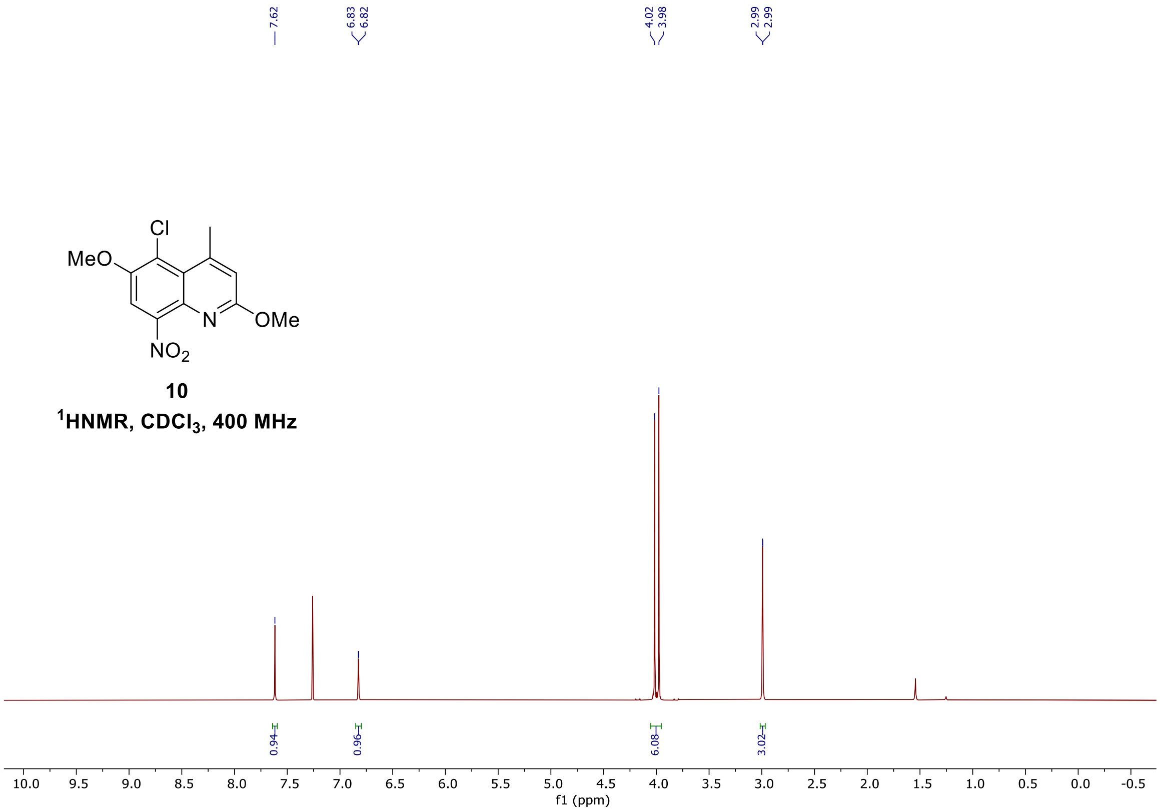
7.62
6.83
6.82
4.02
3.98
2.99
2.99
Cl
MeO
N
OMe
NO2
10
1HNMR, CDCl3, 400 MHz
0.94
0.96
6.08
3.02
10.0 9.5 9.0 8.5 8.0 7.5 7.0 6.5 6.0 5.5 5.0 4.5 4.0 3.5 3.0 2.5 2.0 1.5 1.0 0.5 0.0 -0.5
f1 (ppm)

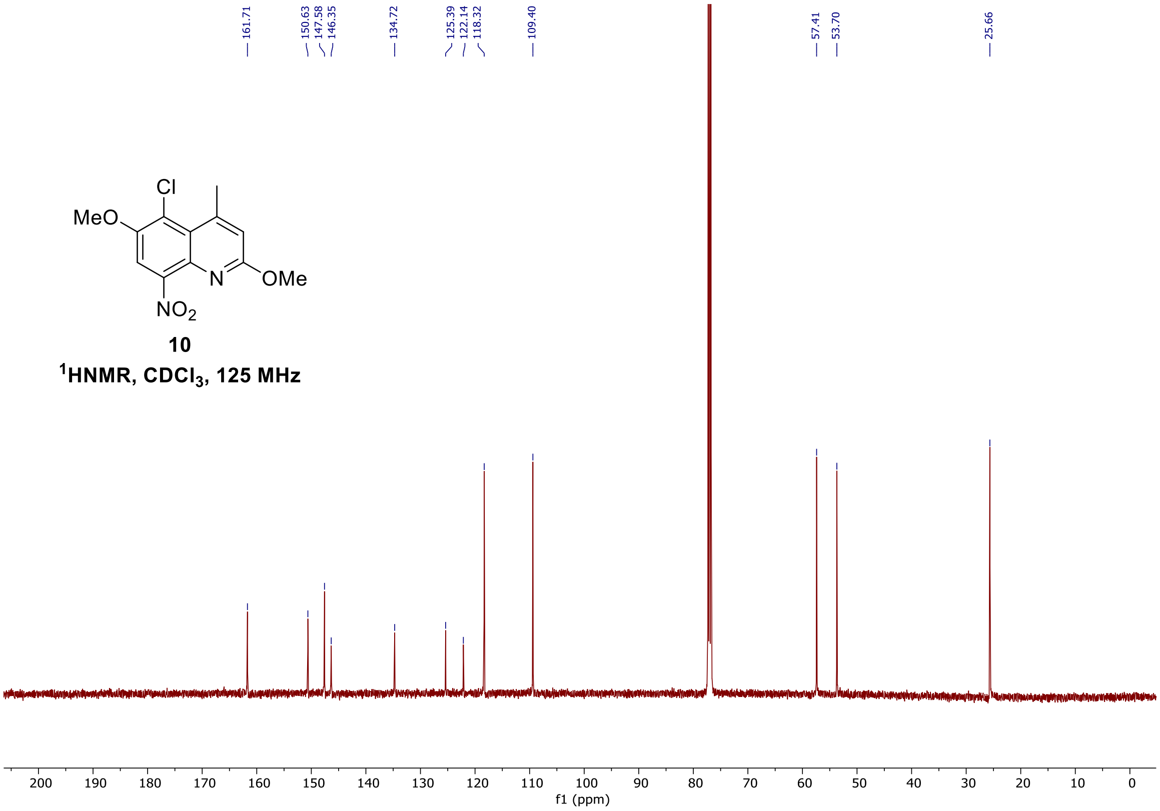
161.71
150.63
147.58
146.35
134.72
125.39
122.14
118.32
109.40
57.41
53.70
25.66
Cl
MeO
N
OMe
NO2
10
1HNMR, CDCl3, 125 MHz
200 190 180 170 160 150 140 130 120 110 100 90 80 70 60 50 40 30 20 10 0
f1 (ppm)

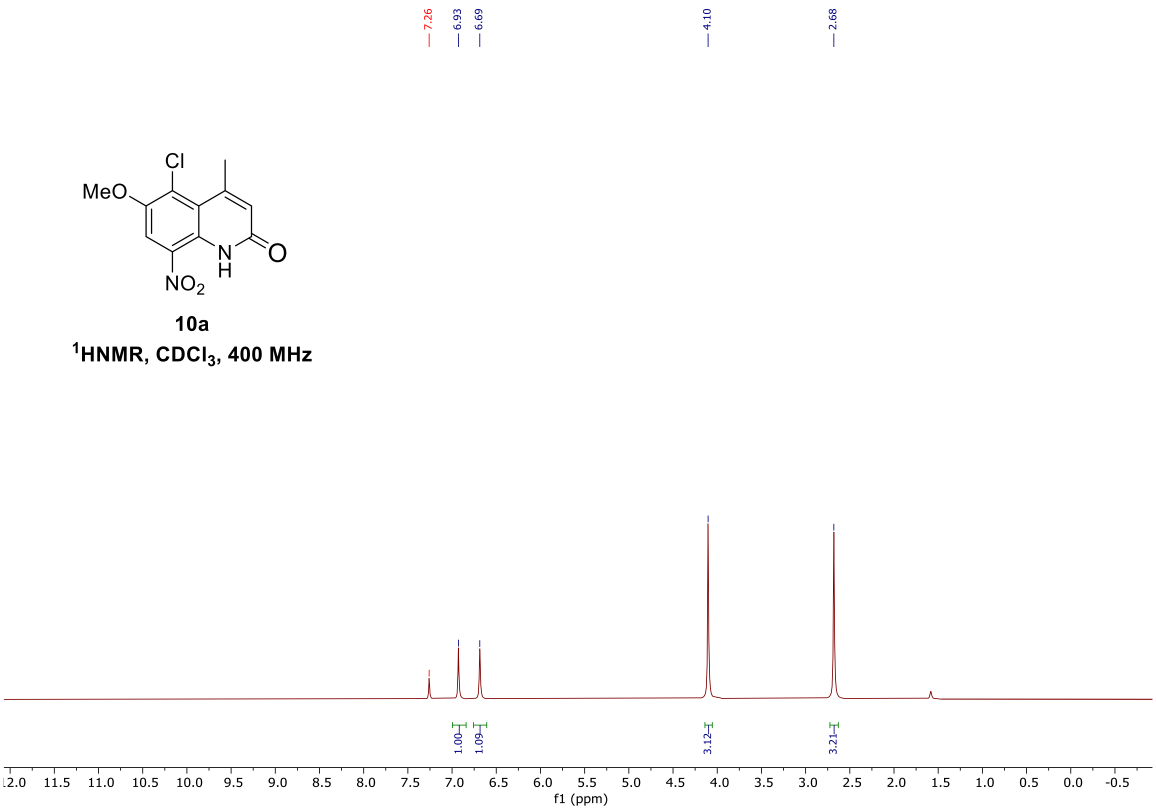

10a

^{1}HNMR, CDCl$_3$, 400 MHz

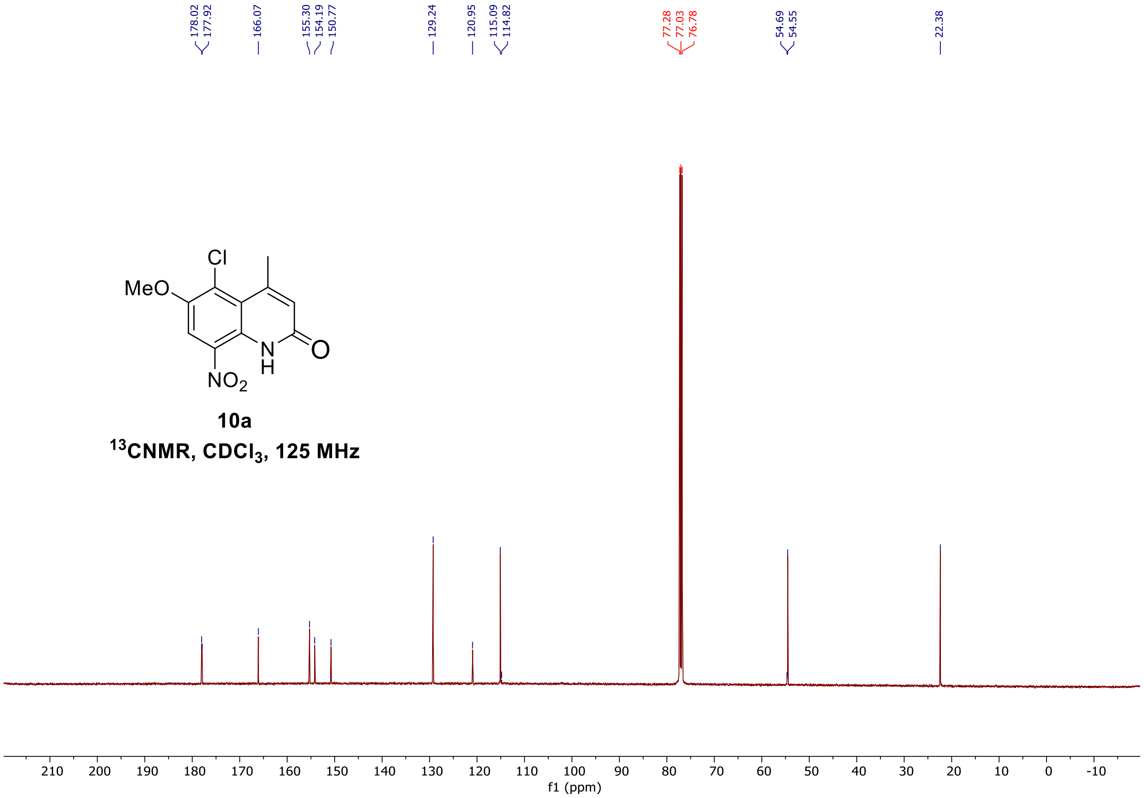

10a

^{13}CNMR, CDCl$_3$, 125 MHz

12

^{1}HNMR, CDCl$_3$, 400 MHz

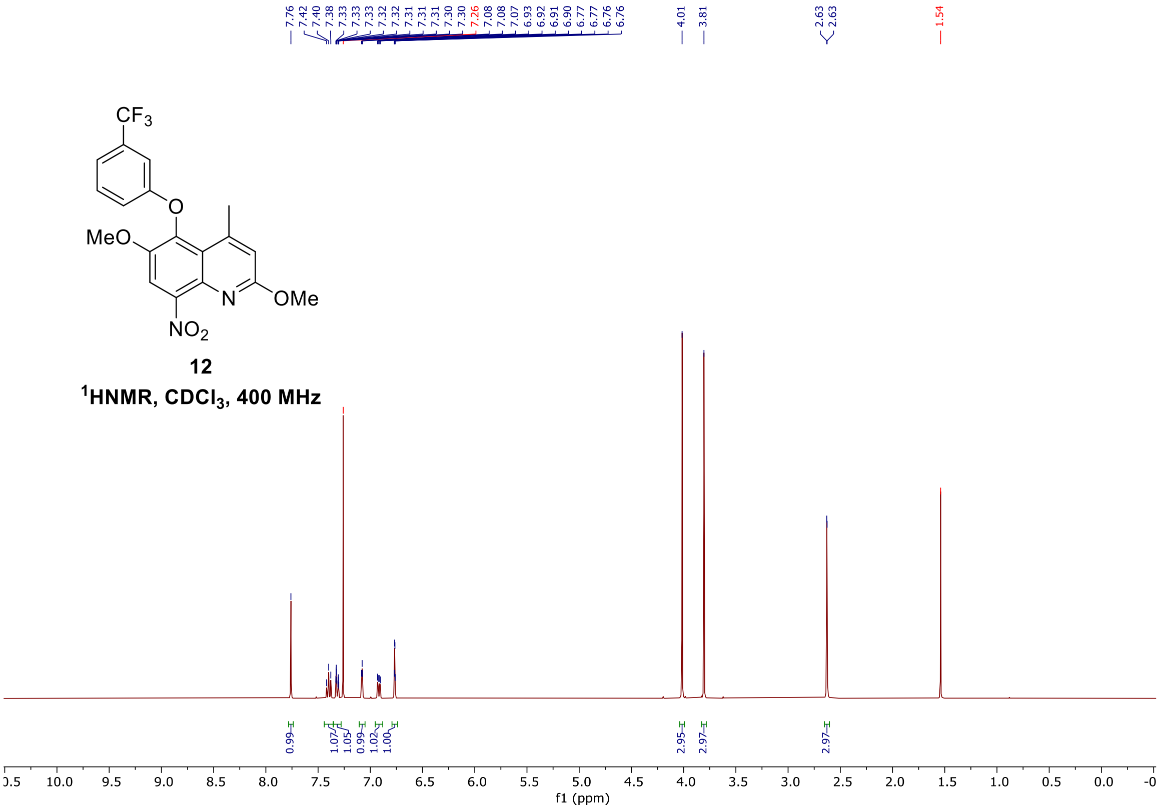

12

^{13}CNMR, CDCl$_3$, 100 MHz

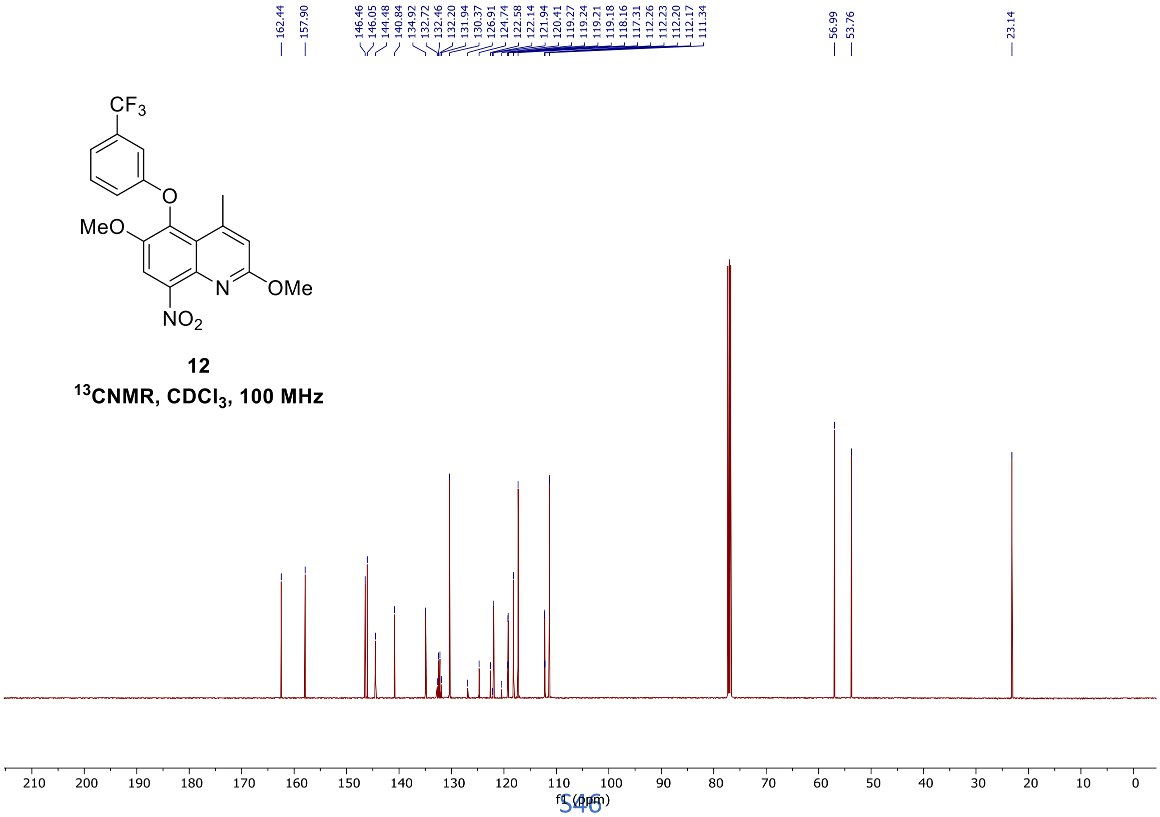

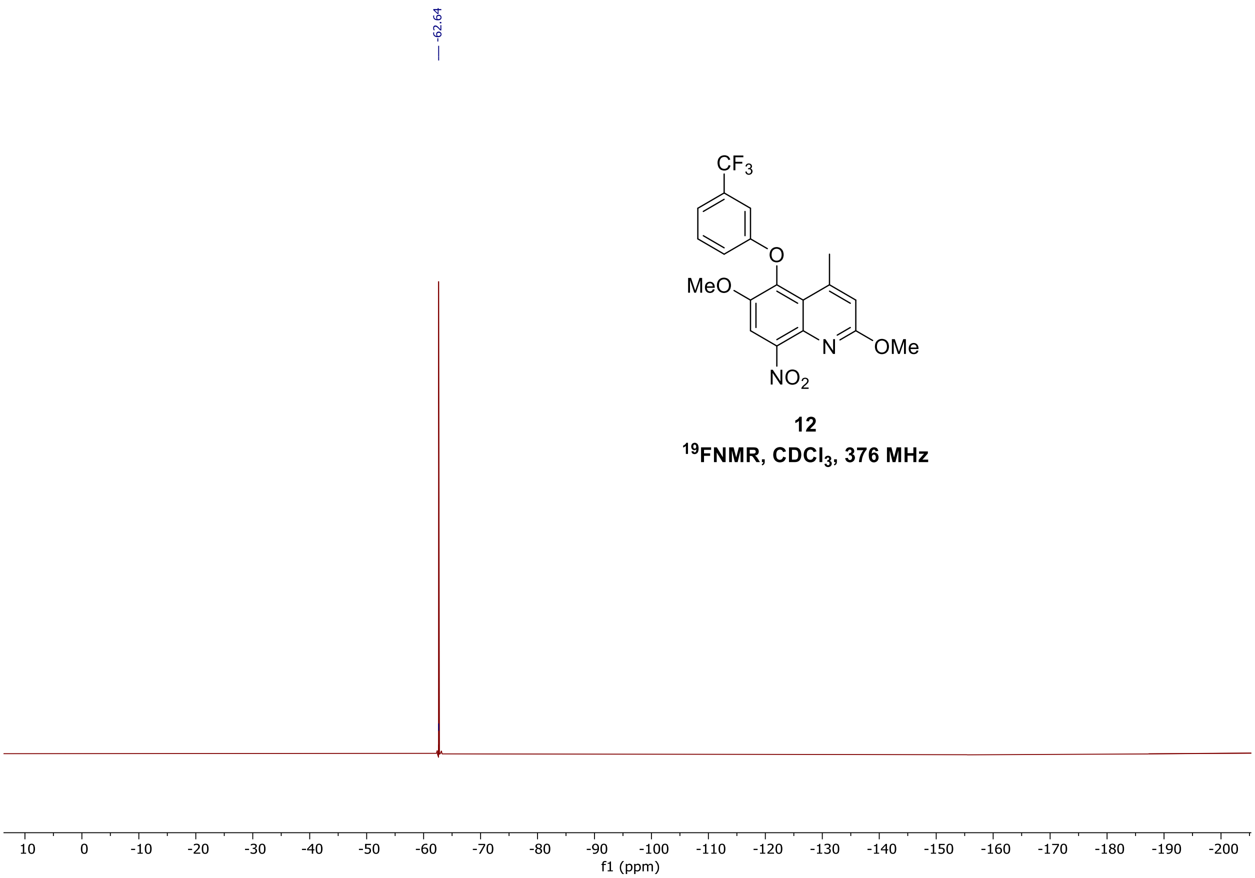

12

^{19}FNMR, $CDCl_3$, 376 MHz

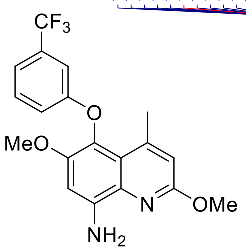

13
^{1}HNMR, $CDCl_3$, 400 MHz

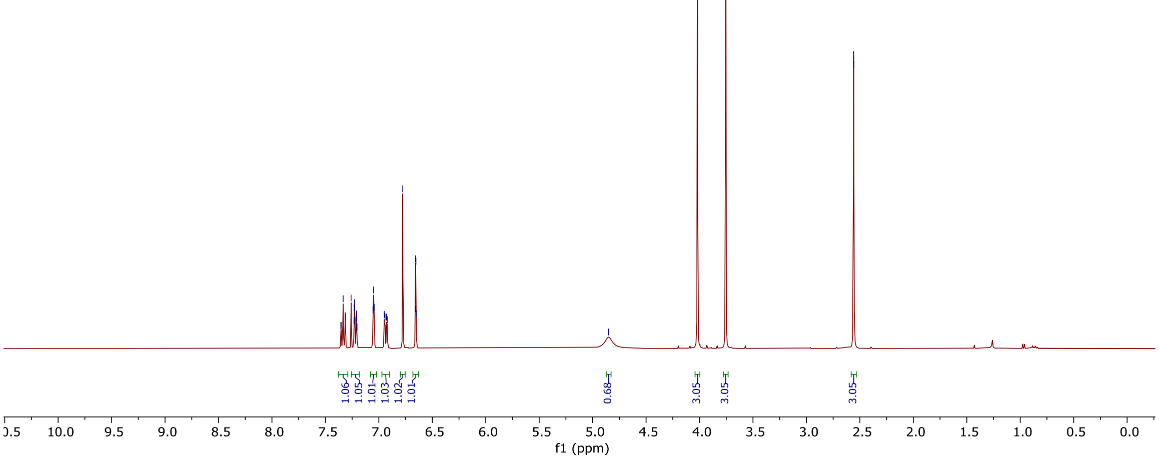

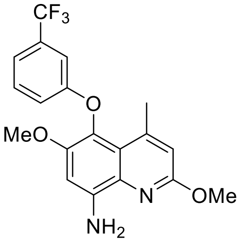

13
^{13}CNMR, $CDCl_3$, 100 MHz

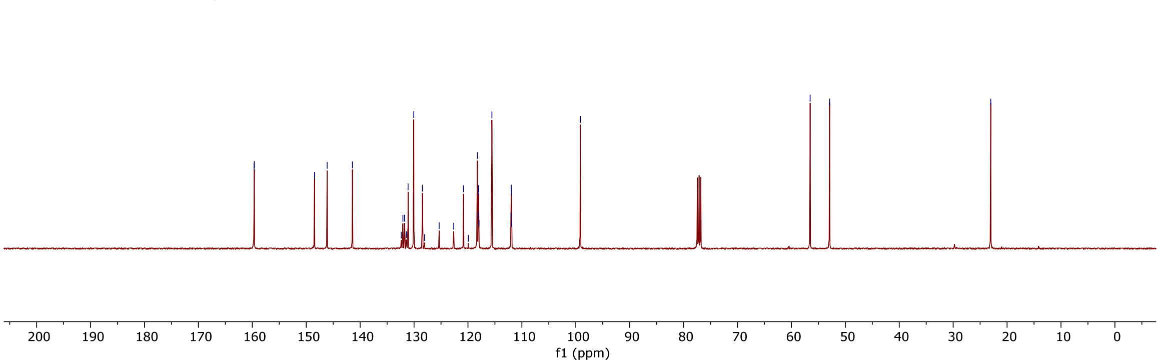

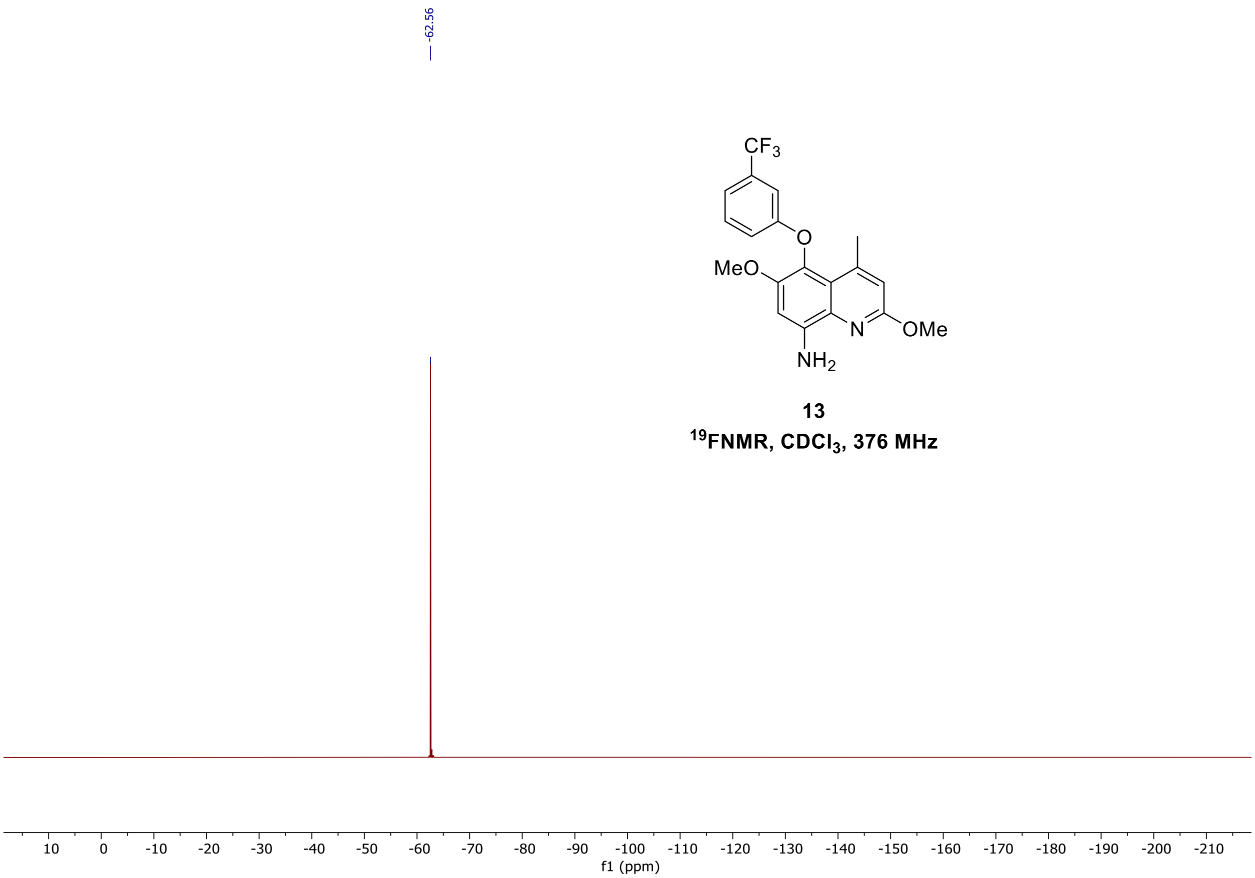

13

^{19}FNMR, CDCl$_3$, 376 MHz

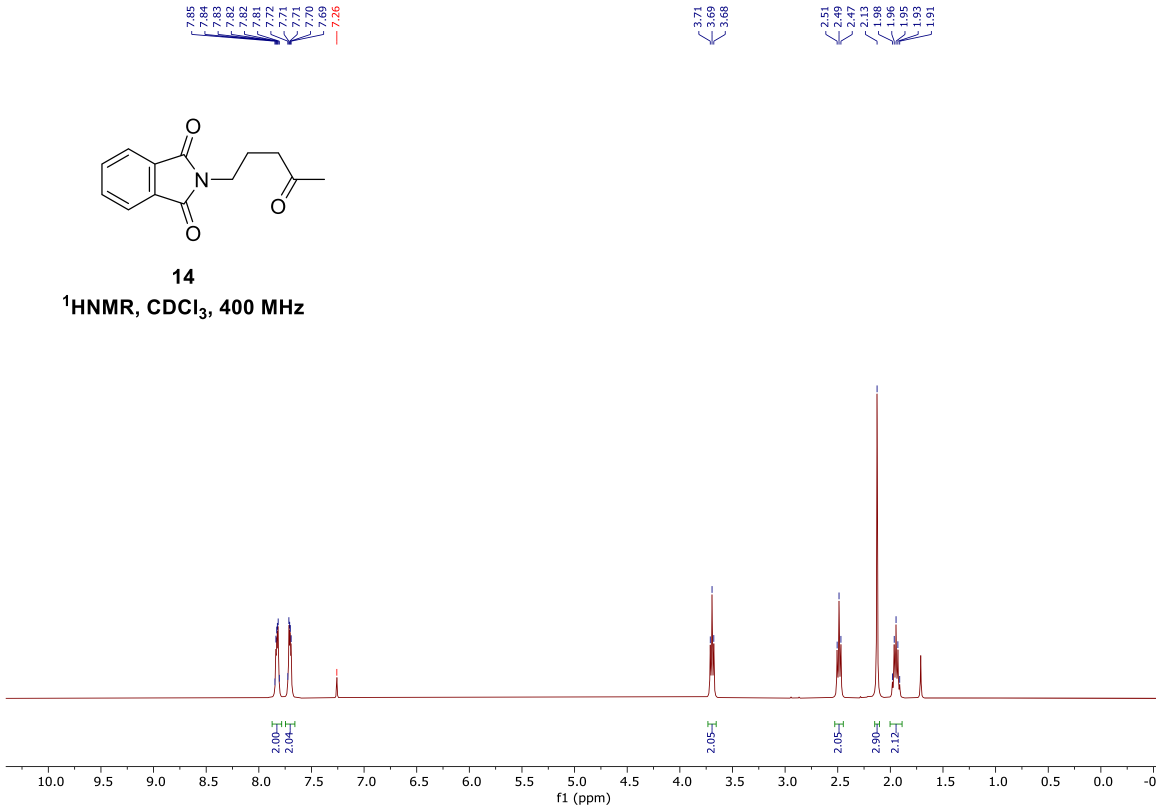
7.85 7.84 7.83 7.82 7.82 7.81 7.72 7.71 7.71 7.70 7.69 7.26
3.71 3.69 3.68
2.51 2.49 2.47 2.13 1.98 1.96 1.95 1.93 1.91
O
N
O
O
14
1HNMR, CDCl3, 400 MHz
2.00 2.04 2.05 2.05 2.90 2.12
10.0 9.5 9.0 8.5 8.0 7.5 7.0 6.5 6.0 5.5 5.0 4.5 4.0 3.5 3.0 2.5 2.0 1.5 1.0 0.5 0.0 -0
f1 (ppm)

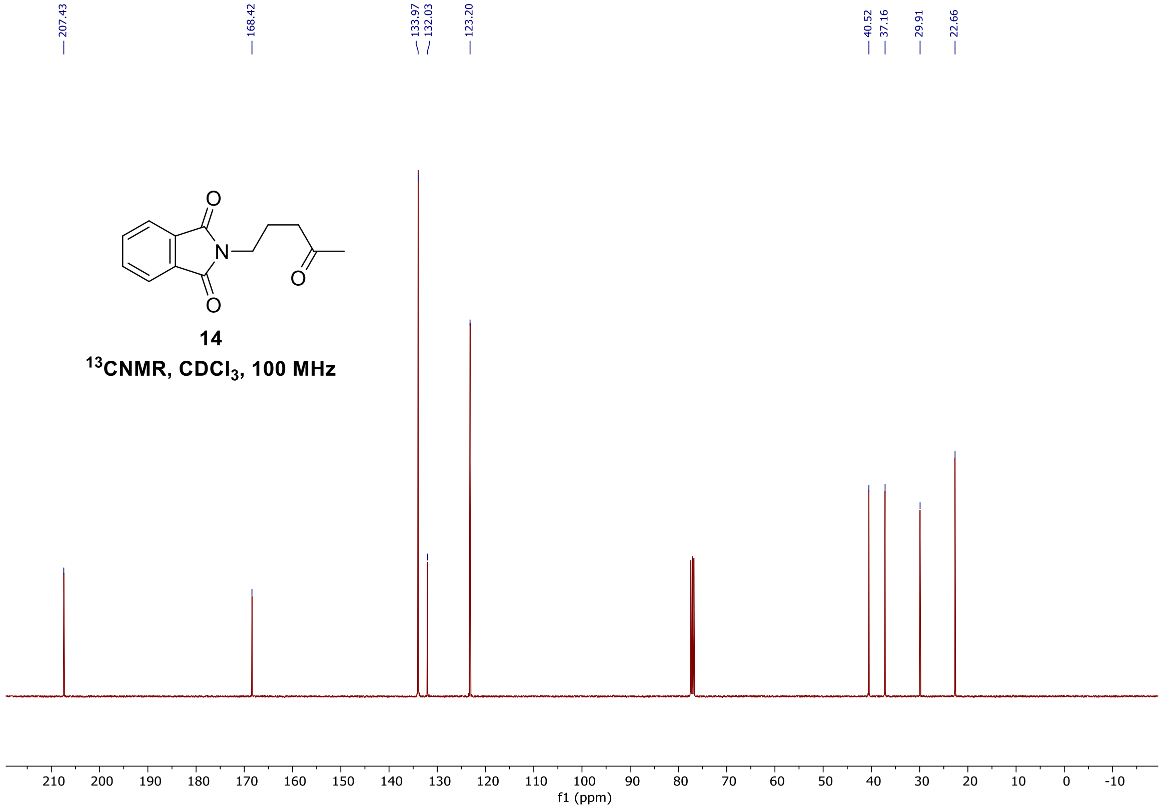
207.43
168.42
133.97 132.03
123.20
40.52 37.16 29.91 22.66
O
N
O
O
14
13CNMR, CDCl3, 100 MHz
210 200 190 180 170 160 150 140 130 120 110 100 90 80 70 60 50 40 30 20 10 0 -10
f1 (ppm)

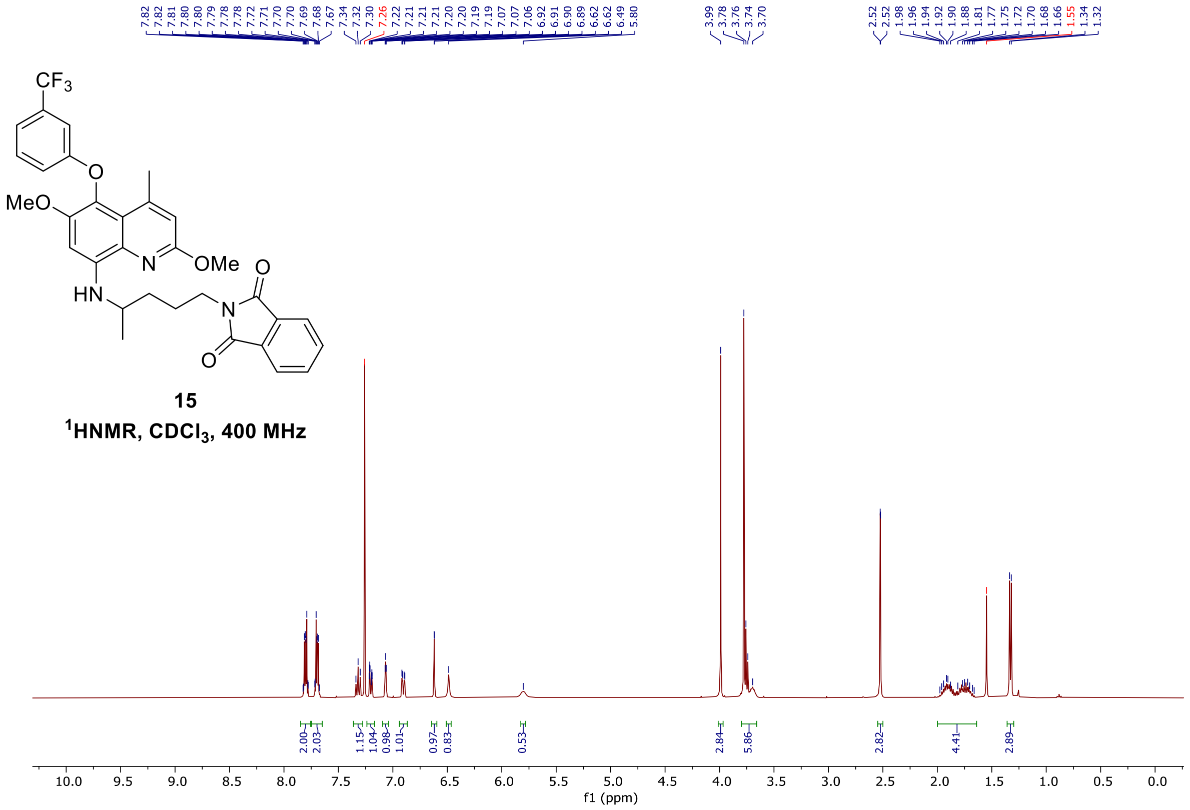
15
1HNMR, CDCl3, 400 MHz
f1 (ppm)

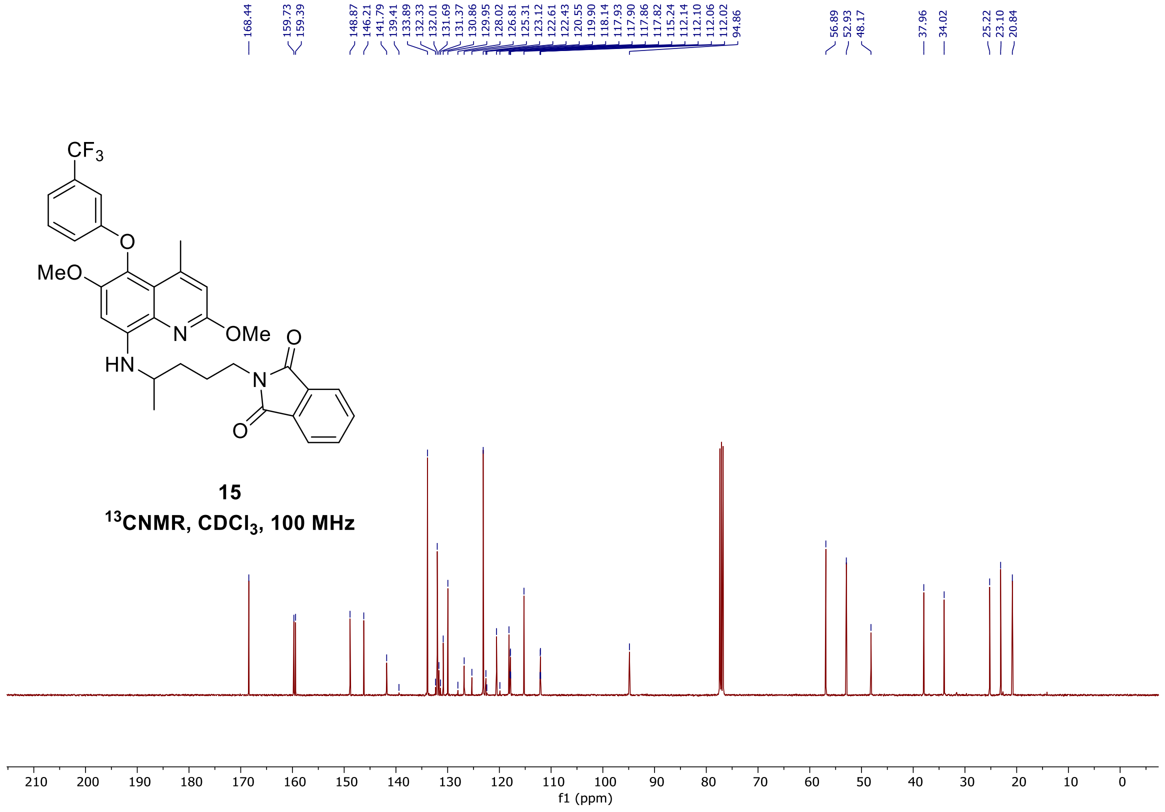
15
13CNMR, CDCl3, 100 MHz
f1 (ppm)

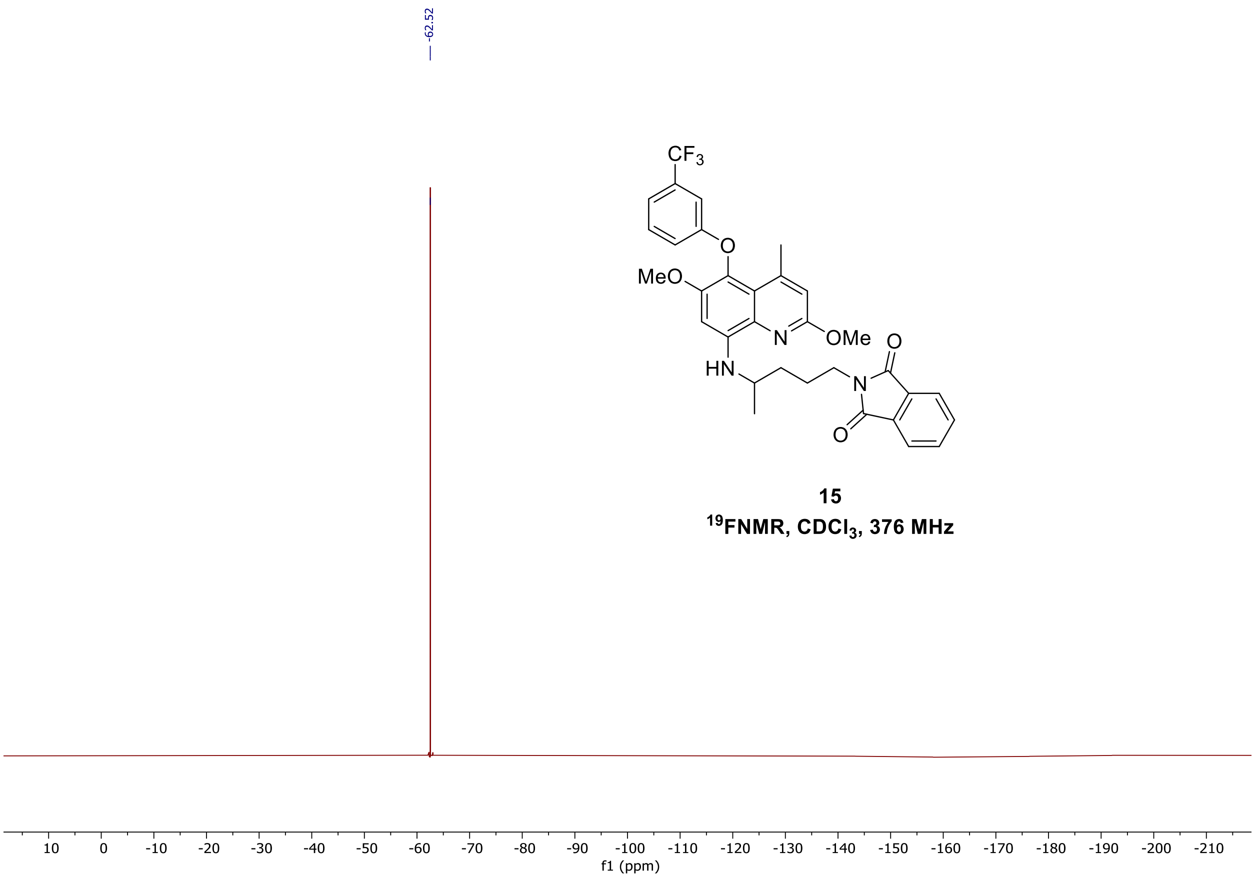

15

^{19}FNMR, CDCl$_3$, 376 MHz

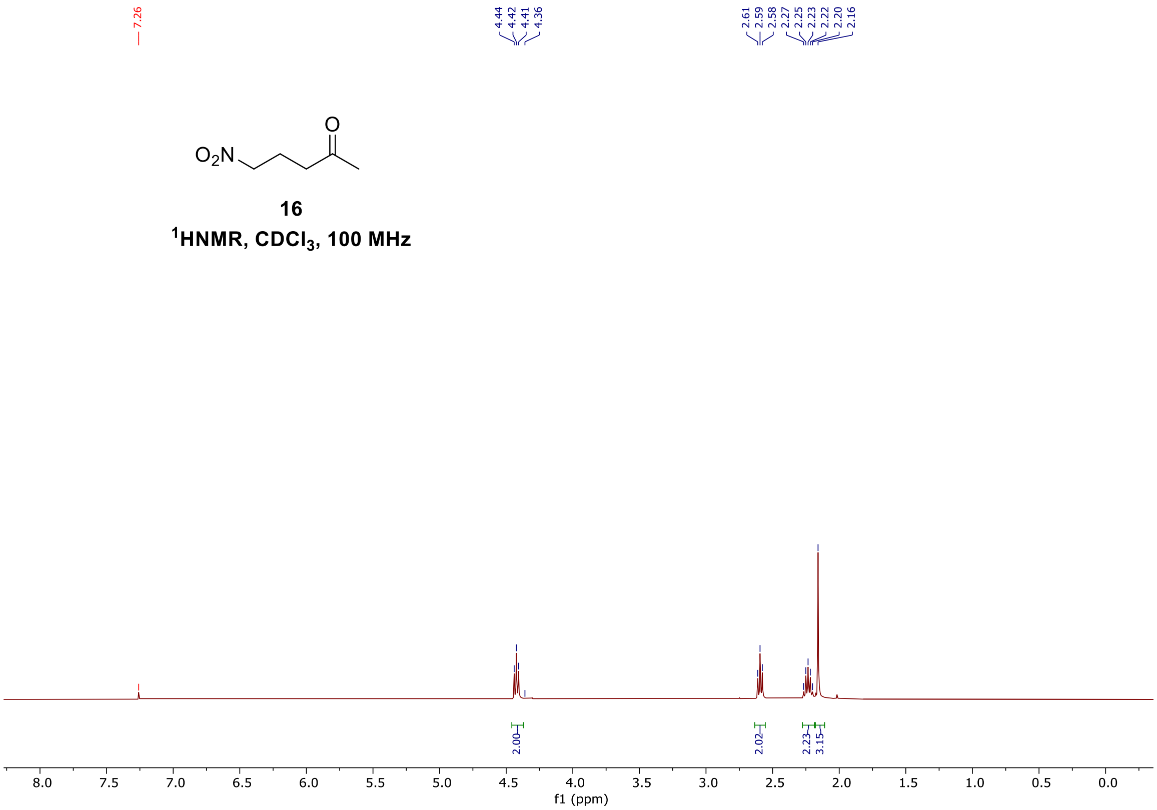

16

^{1}HNMR, $CDCl_3$, 100 MHz

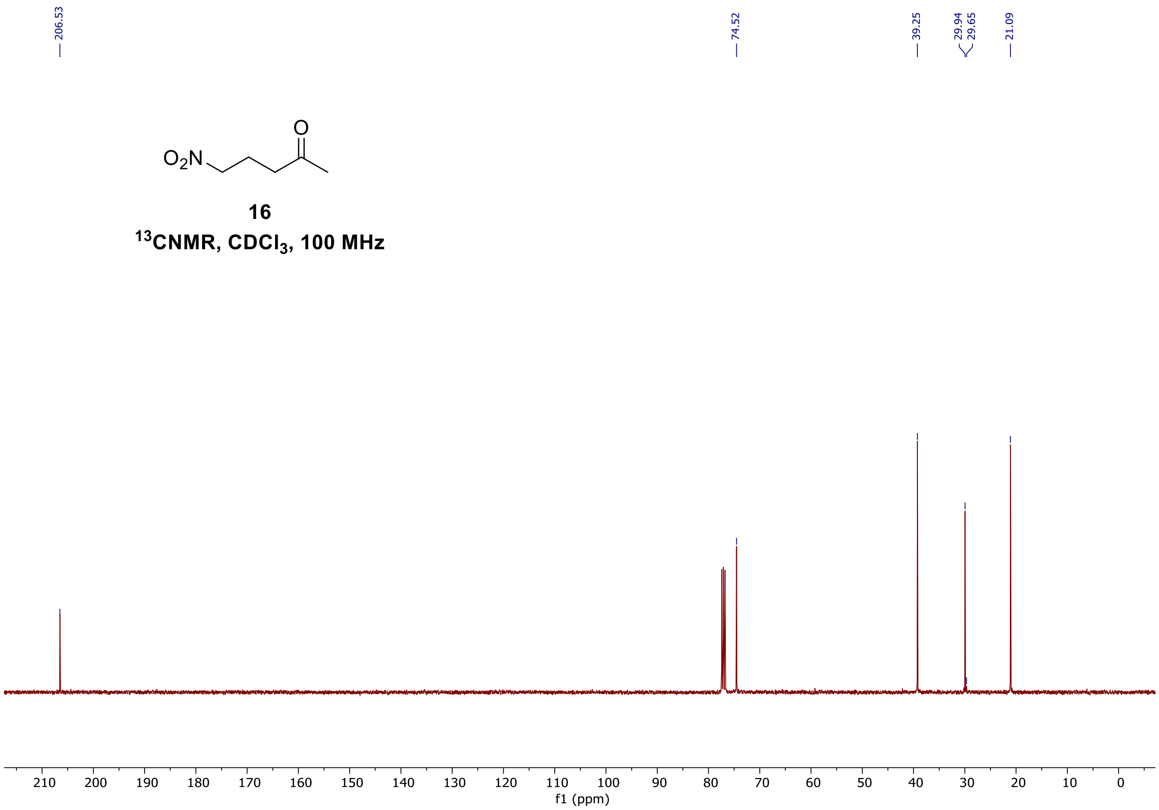

16

^{13}CNMR, $CDCl_3$, 100 MHz

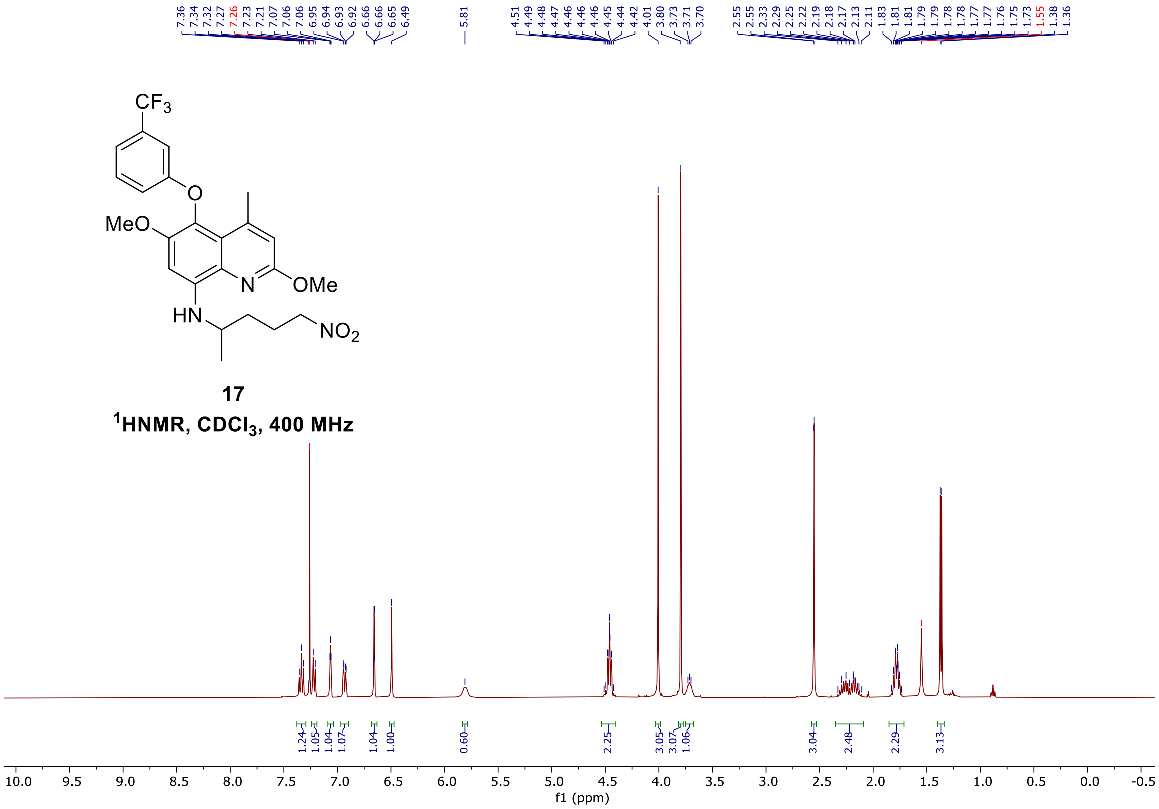
7.36 7.34 7.32 7.27 7.26 7.23 7.21 7.07 7.06 7.06 6.95 6.94 6.93 6.92 6.66 6.66 6.65 6.49
5.81
4.51 4.49 4.48 4.47 4.46 4.46 4.46 4.45 4.44 4.42 4.01 3.80 3.73 3.71 3.70
2.55 2.55 2.33 2.29 2.25 2.22 2.19 2.18 2.17 2.13 2.11 1.83 1.81 1.81 1.79 1.79 1.78 1.78 1.77 1.77 1.76 1.75 1.73 1.55 1.38 1.36
CF3
O
MeO
N
OMe
HN
NO2
17
1HNMR, CDCl3, 400 MHz
1.24 1.05 1.04 1.07 1.04 1.00 0.60 2.25 3.05 3.07 1.06 3.04 2.48 2.29 3.13
10.0 9.5 9.0 8.5 8.0 7.5 7.0 6.5 6.0 5.5 5.0 4.5 4.0 3.5 3.0 2.5 2.0 1.5 1.0 0.5 0.0 -0.5
f1 (ppm)

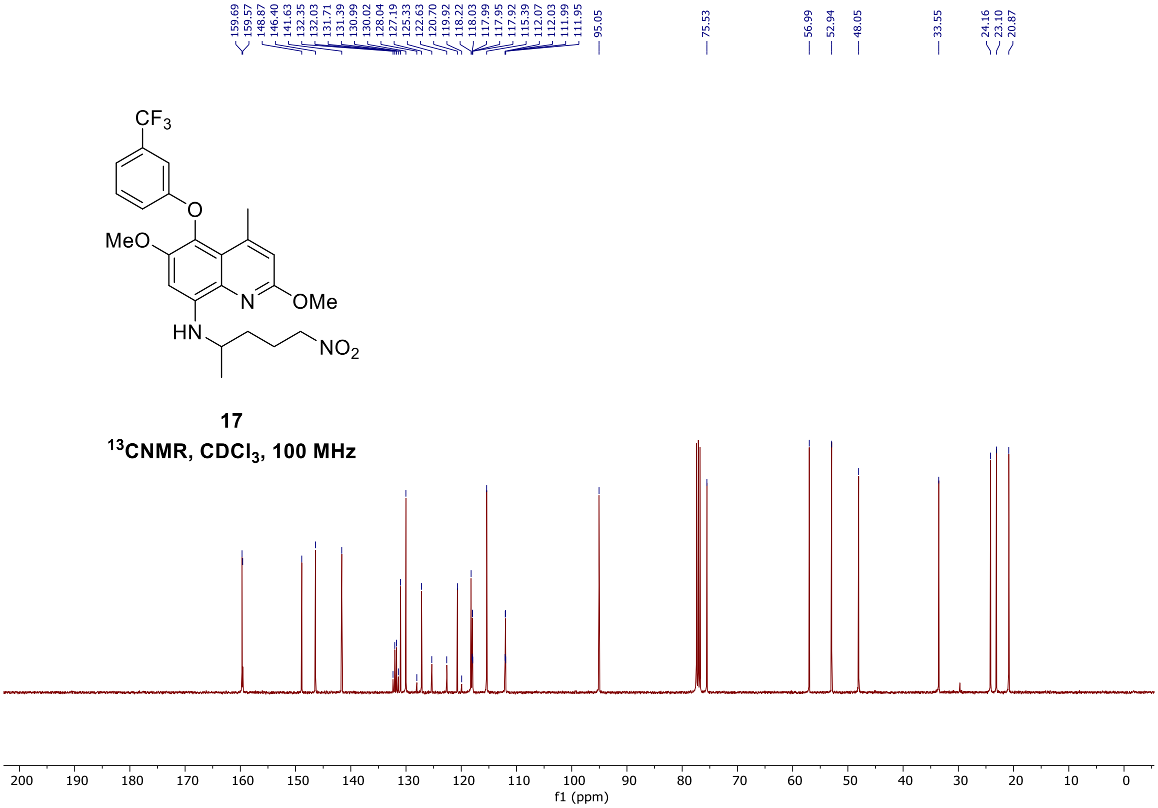
159.69 159.57 148.87 146.40 141.63 132.35 132.03 131.71 131.39 130.99 130.02 128.04 127.19 125.33 122.63 120.70 119.92 118.22 118.03 117.99 117.95 117.92 115.39 112.07 112.03 111.99 111.95
95.05
75.53
56.99
52.94
48.05
33.55
24.16 23.10 20.87
CF3
O
MeO
N
OMe
HN
NO2
17
13CNMR, CDCl3, 100 MHz
200 190 180 170 160 150 140 130 120 110 100 90 80 70 60 50 40 30 20 10 0
f1 (ppm)

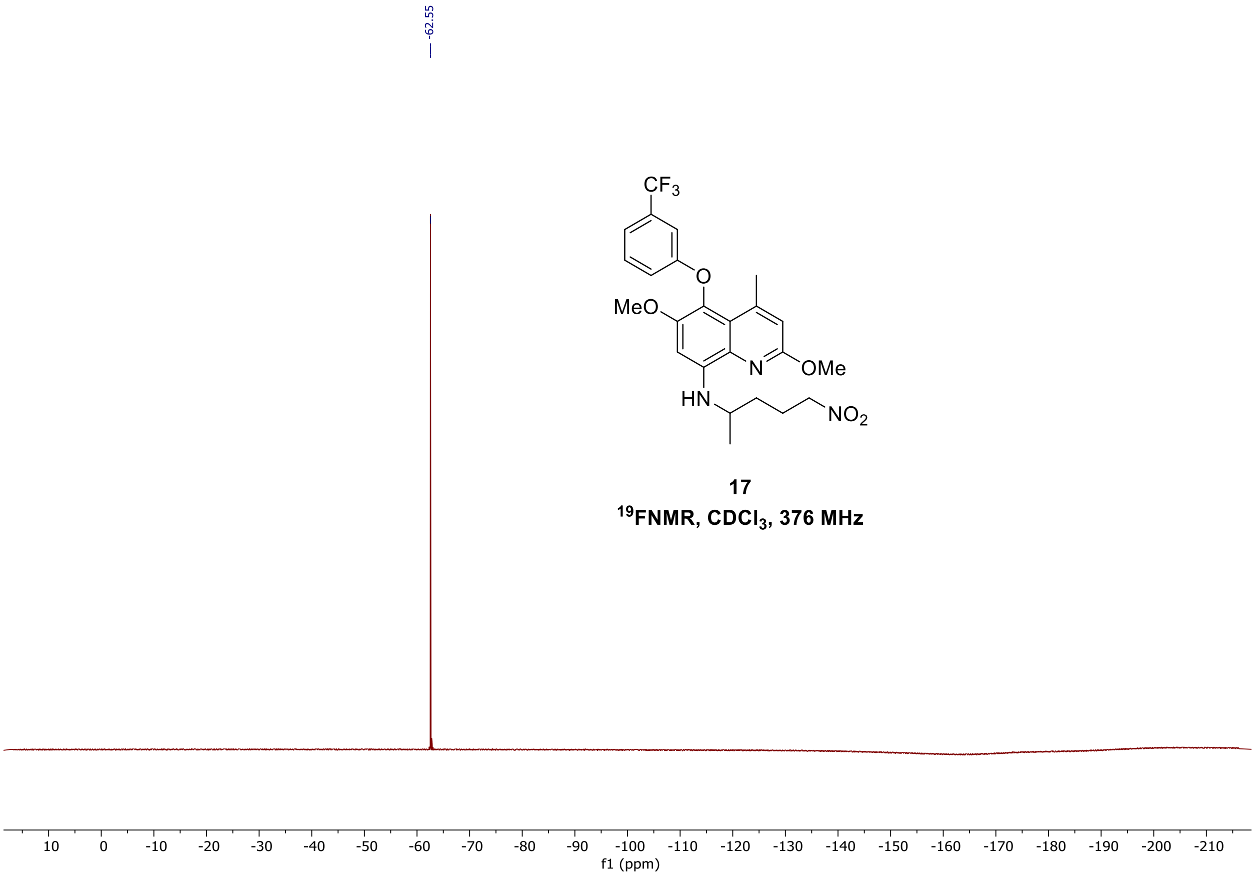

17

^{19}FNMR, $CDCl_3$, 376 MHz

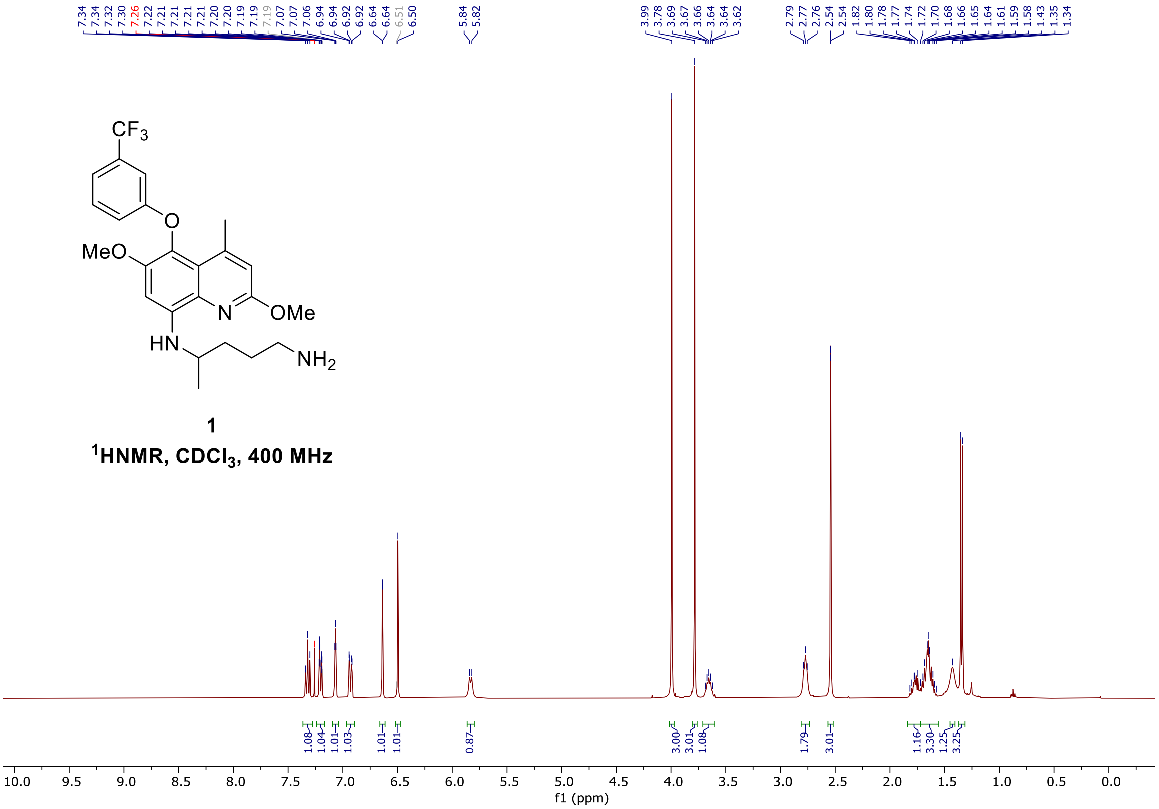

7.34 7.34 7.32 7.30 7.26 7.22 7.21 7.21 7.21 7.21 7.20 7.20 7.19 7.19 7.19 7.07 7.07 7.06 6.94 6.94 6.92 6.92 6.64 6.64 6.51 6.50
5.84 5.82
3.99 3.78 3.69 3.67 3.66 3.64 3.64 3.62
2.79 2.77 2.76 2.54 2.54 1.82 1.80 1.78 1.77 1.74 1.72 1.70 1.68 1.66 1.65 1.64 1.61 1.59 1.58 1.43 1.35 1.34
CF3
MeO
O
N
OMe
HN
NH2
1
1HNMR, CDCl3, 400 MHz
1.08 1.04 1.01 1.03 1.01 1.01 0.87 3.00 3.01 1.08 1.79 3.01 1.16 3.30 1.25 3.25
f1 (ppm)

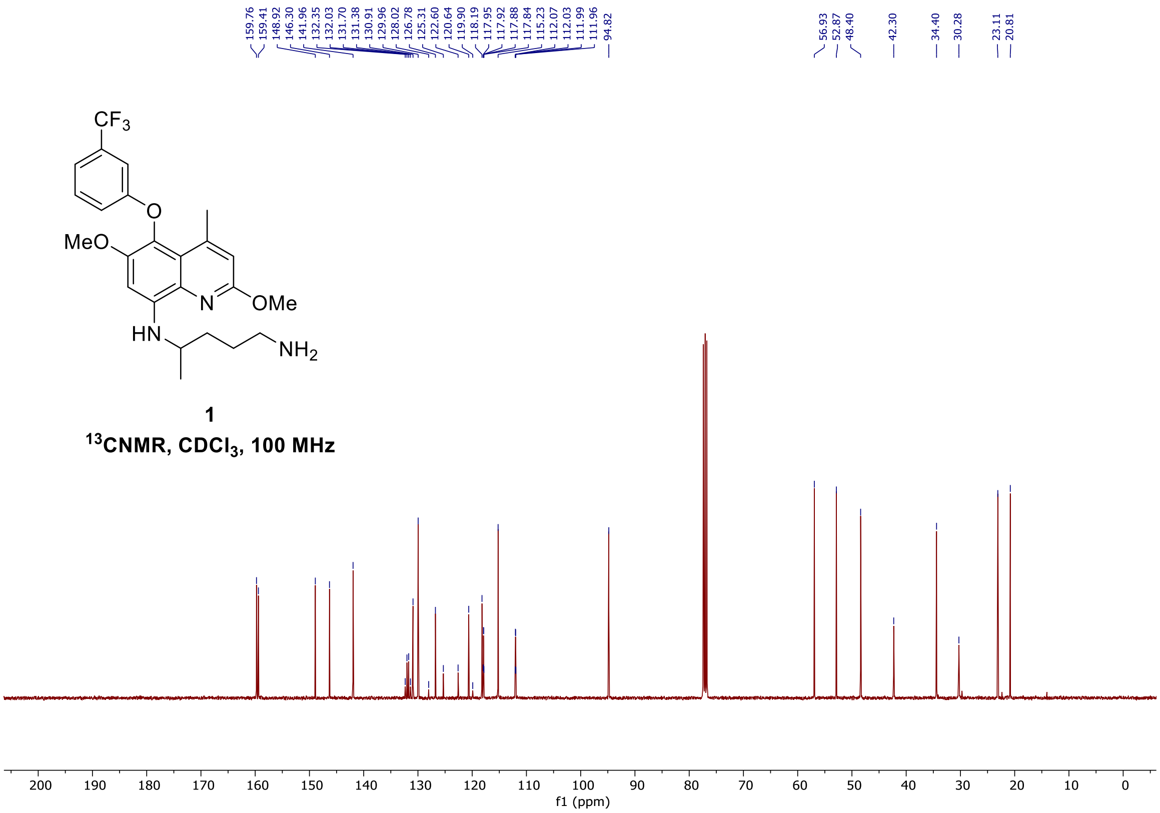

159.76 159.41 148.92 146.30 141.96 132.35 132.03 131.70 131.38 130.91 129.96 128.02 126.78 125.31 122.60 120.64 119.90 118.19 117.95 117.92 117.88 117.84 115.23 112.07 112.03 111.99 111.96 94.82
56.93 52.87 48.40 42.30 34.40 30.28 23.11 20.81
CF3
MeO
O
N
OMe
HN
NH2
1
13CNMR, CDCl3, 100 MHz
f1 (ppm)

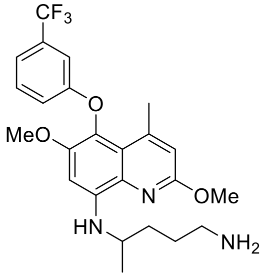

1

^{19}F NMR, $CDCl_3$, 100 MHz

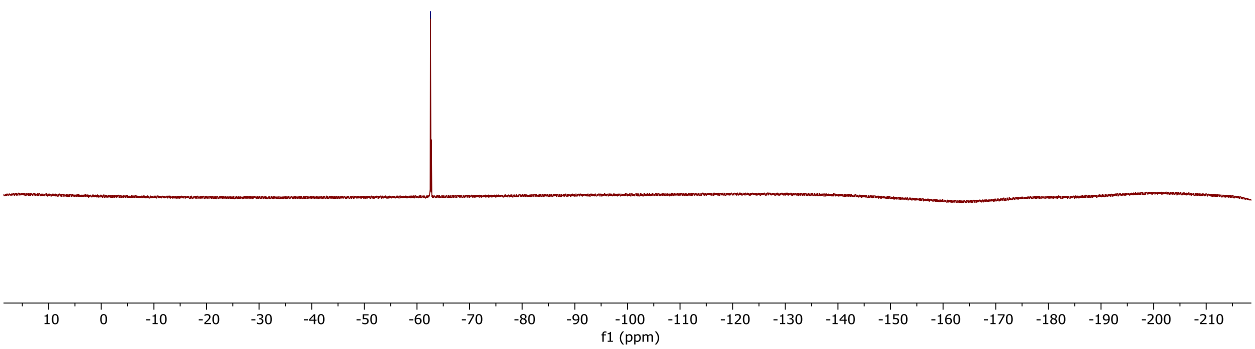

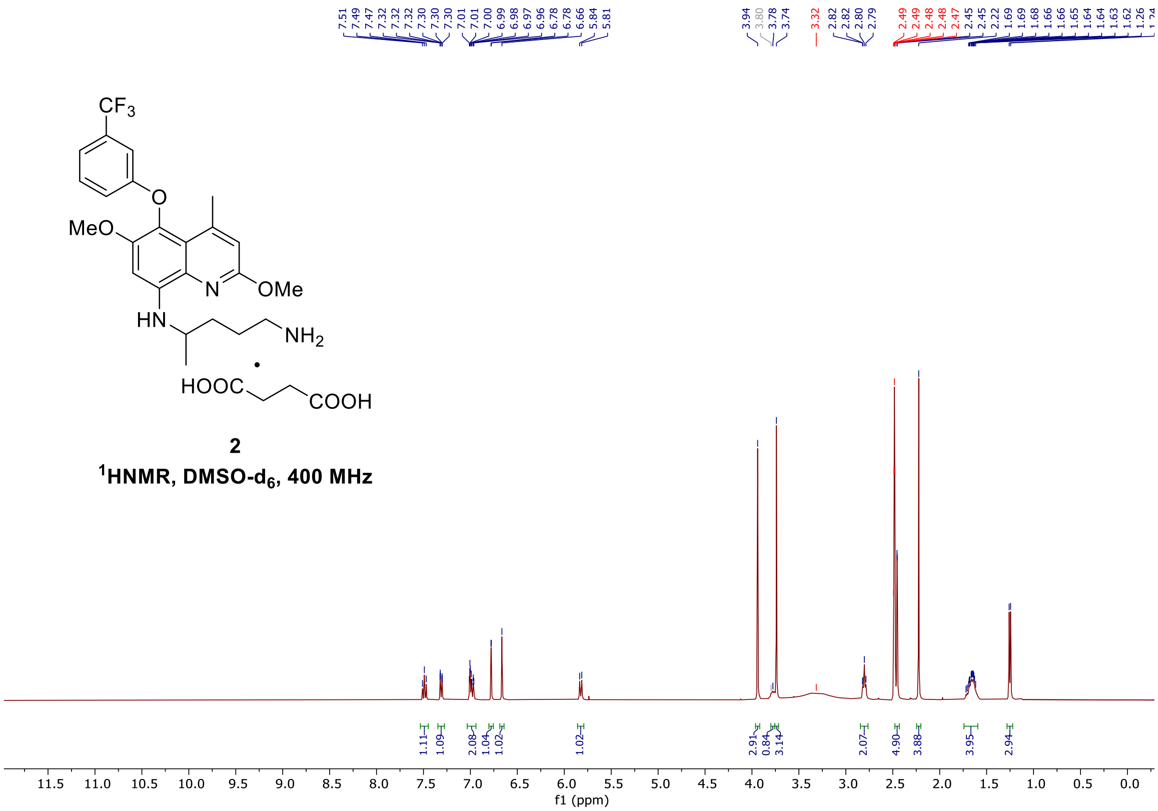

7.51 7.49 7.47 7.32 7.32 7.32 7.30 7.30 7.30 7.01 7.01 7.00 6.99 6.98 6.97 6.96 6.78 6.78 6.66 5.84 5.81
3.94 3.80 3.78 3.74 3.32 2.82 2.82 2.80 2.79 2.49 2.49 2.48 2.48 2.47 2.45 2.45 2.22 1.69 1.68 1.66 1.66 1.65 1.64 1.64 1.63 1.62 1.26
CF3
O
MeO
N
OMe
HN
NH2
HOOC
COOH
2
1HNMR, DMSO-d6, 400 MHz
1.11 1.09 2.08 1.04 1.02 1.02 2.91 0.84 3.14 2.07 4.90 3.88 3.95 2.94
11.5 11.0 10.5 10.0 9.5 9.0 8.5 8.0 7.5 7.0 6.5 6.0 5.5 5.0 4.5 4.0 3.5 3.0 2.5 2.0 1.5 1.0 0.5 0.0
f1 (ppm)

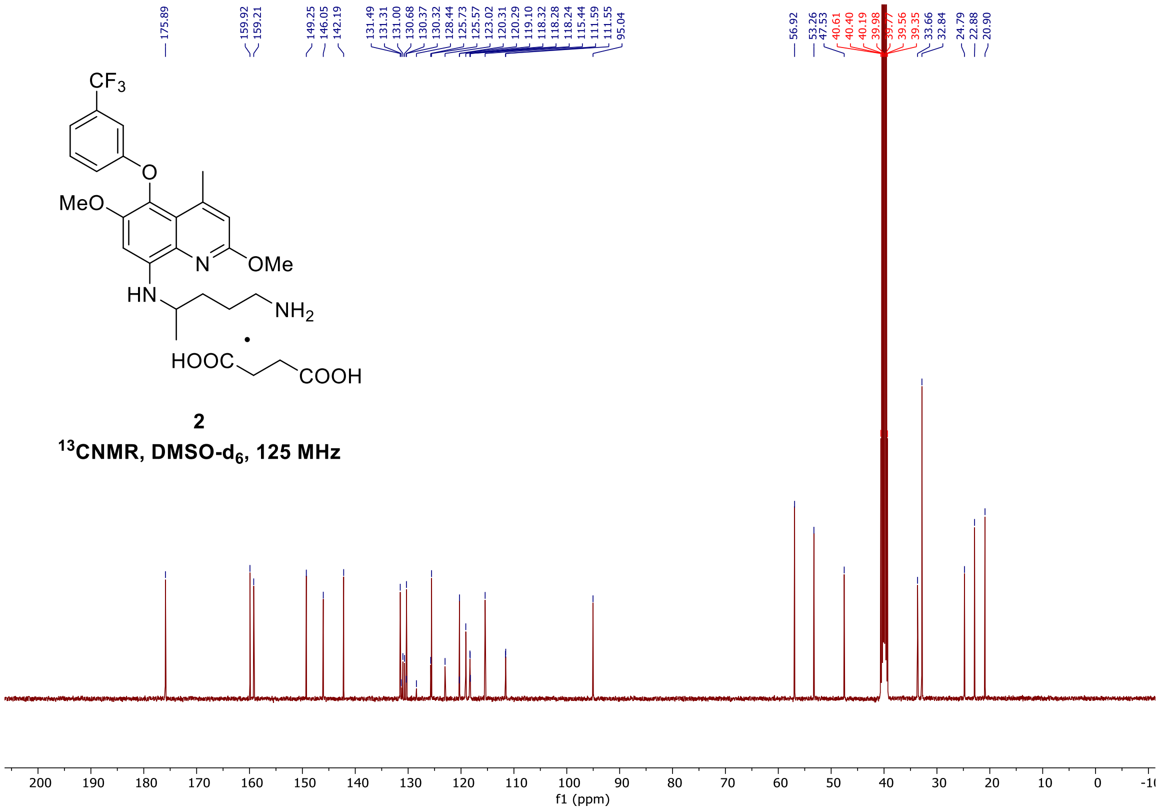

175.89 159.92 159.21 149.25 146.05 142.19 131.49 131.31 131.00 130.68 130.37 130.32 128.44 125.73 125.57 123.02 120.31 120.29 119.10 118.32 118.28 118.24 115.44 111.59 111.55 95.04
56.92 53.26 47.53 40.61 40.40 40.19 39.98 39.77 39.56 39.35 33.66 32.84 24.79 22.88 20.90
CF3
O
MeO
N
OMe
HN
NH2
HOOC
COOH
2
13CNMR, DMSO-d6, 125 MHz
200 190 180 170 160 150 140 130 120 110 100 90 80 70 60 50 40 30 20 10 0
f1 (ppm)

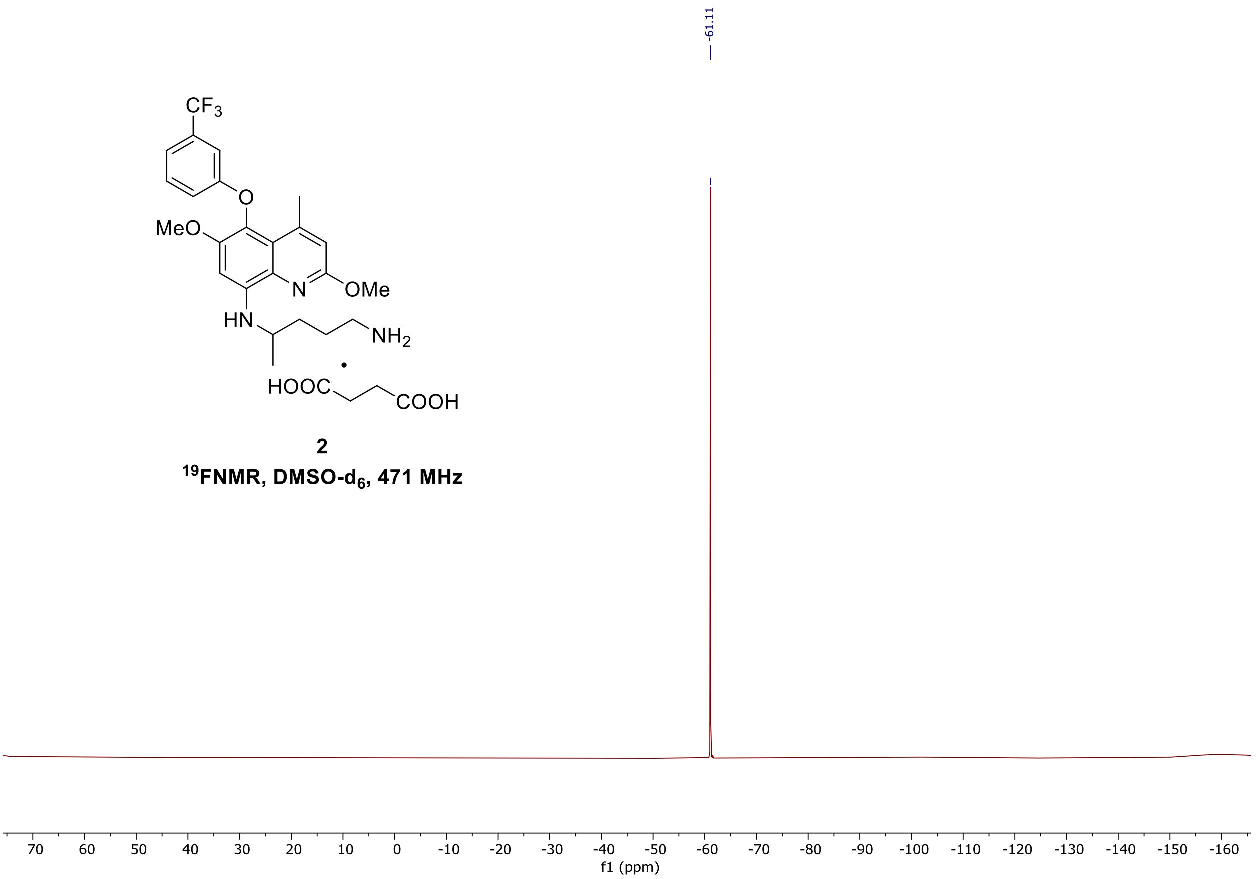

2

^{19}FNMR, DMSO-d_6, 471 MHz
